# Traditional herbal medicine for obesity-related polycystic ovary syndrome: a meta-analysis and data mining study

**DOI:** 10.3389/fphar.2025.1738172

**Published:** 2026-01-20

**Authors:** Lei Tang, Haijuan Liu, Ying Pang, Guohua Wang, Zheng Wang, Tianyao Lv

**Affiliations:** 1 Beijing University of Chinese Medicine, Beijing, China; 2 Beijing University of Chinese Medicine Third Affiliated Hospital, Beijing, China; 3 Peking University Third Hospital, Beijing, China; 4 Beijing Nuclear Industry Hospital, Beijing, China; 5 Sanlitun Community Health Service Center, Beijing, China

**Keywords:** clinical efficacy, evidence-basedmedicine, obesity, polycystic ovary syndrome, traditional herbal medicine

## Abstract

**Objective:**

To systematically evaluate the clinical efficacy of traditional herbal medicine (THM) as an adjunctive therapy for obesity-related polycystic ovary syndrome (PCOS) and to identify core botanical drug combinations using evidence synthesis and data mining approaches.

**Methods:**

We searched six databases from inception to March 2025 for randomized controlled trials Meta-analyses were performed using Stata 15.1. Association rule mining was performed with the *Apriori* algorithm. The Cochrane Risk of Bias Assessment Tool (ROB 2.0) and the Grading of Recommendations Assessment, Development, and Evaluation (GRADE) framework were used to evaluate risk of bias and evidence certainty, respectively.

**Results:**

Seventy-two RCTs involving 5,308 patients were included. Meta-analysis indicated that THM combined with conventional therapy significantly improved the clinical efficacy rate (OR = 3.73, 95% CI: 3.12 to 4.46, p < 0.001; low certainty) and clinical pregnancy rate (OR = 3.03, 95% CI: 2.05 to 4.48, p < 0.001; moderate certainty). Significant improvements were also observed in Homeostatic Model Assessment of Insulin Resistance (HOMA-IR) (SMD = −0.81, 95% CI: −1.02 to −0.60, p < 0.001; very low certainty), body mass index (BMI) (SMD = −0.95, 95% CI: −1.09 to −0.81, p < 0.001; low certainty), total testosterone (TT) (SMD = −0.90, 95% CI: −1.10 to −0.69, p < 0.001; very low certainty), and the luteinizing hormone/follicle-stimulating hormone (LH/FSH) ratio (SMD = −0.88, 95% CI: −1.05 to −0.70, p < 0.001; low certainty). Sensitivity analyses confirmed robustness, but substantial heterogeneity (I^2^ up to 89.3%) and publication bias (Egger’s test p < 0.05 for several outcomes) were noted. Association rule mining identified Poria cocos, Citrus reticulata, Atractylodes lancea, and Cyperus rotundus as the most strongly associated core botanical drug combination.

**Conclusion:**

THM demonstrated superiority over conventional treatment alone in improving key clinical, metabolic, and reproductive outcomes in obesity-related PCOS. Association rule analysis revealed a core botanical drug combination as promising candidates for future research. However, more rigorously designed, large-scale RCTs are required.

**Systematic Review Registration:**

https://www.crd.york.ac.uk/PROSPERO/view/CRD420251111078, identifier CRD420251111078.

## 1 Introduction

Polycystic ovary syndrome (PCOS) is a common endocrine and metabolic disorder affecting women of reproductive age, with rising global prevalence and increasingly younger age at diagnosis (Singh et al., 2023). Obesity and PCOS are closely intertwined, and their coexistence significantly increases the long-term risks of cardiometabolic diseases and infertility (Anagnostis et al., 2018; Zhang et al., 2020). While modern medicine offers various treatments for PCOS, such as metformin and combined oral contraceptives, these options are often associated with side effects during long-term use (Renato, 2015). Consequently, many patients seek gentler and more comprehensive therapeutic approaches.

Traditional herbal medicine (THM), comprising medicinal plants used across multiple traditional medical systems, has shown potential in modulating endocrine and metabolic functions (Chen et al., 2023). However, robust evidence specifically supporting its efficacy in obesity-related PCOS remains limited. A key challenge lies in identifying and validating core botanical drug combinations derived from clinical practice.

Therefore, this study aimed to rigorously and comprehensively evaluate the efficacy of THM as an adjunctive therapy for obesity-related PCOS in adults. We conducted a systematic review and meta-analysis of randomized controlled trials (RCTs) that met strict inclusion criteria, with a focus on synthesizing patient-important clinical outcomes to ensure the relevance of our findings. Beyond quantifying clinical effects, we applied association rule mining to analyze the composition of the THM formulas, to identify core botanical drug combinations associated with positive therapeutic outcomes. This integrated approach provides both a conclusive summary of clinical efficacy and data-driven insights into the characteristic combinations that define this treatment strategy, thereby offering a more nuanced understanding of the existing evidence base.

## 2 Materials and methods

This study was conducted in accordance with the Preferred Reporting Items for Systematic Reviews and Meta-Analyses (PRISMA 2020) statement (Page et al., 2021) and was registered in the Prospective Register of Systematic Review (PROSPERO) database (Registration number: CRD420251111078).

### 2.1 Data sources and search strategy

A systematic search was performed across six databases: China National Knowledge Infrastructure (CNKI), Wanfang Data, VIP Chinese Science and Technology Periodical Database (VIP), Chinese Biomedical Literature Service System (SinoMed), PubMed, and Web of Science. The search covered the period from database inception to 15 March 2025. Both computerized and manual search methods were used to ensure comprehensiveness. Search strategies incorporated Medical Subject Headings (MeSH) and free-text terms. Search terms were standardized using the Thesaurus of Chinese Traditional Medicine and MeSH terminology. Detailed search strategies are provided in Supplementary Table S1.

### 2.2 Study selection

Two investigators (LT and HjL) independently screened titles and abstracts of all retrieved records against pre-defined eligibility criteria. Full texts of potentially relevant studies were then reviewed for final inclusion. Discrepancies were resolved through consensus or consultation with a third researcher (YP).

#### 2.2.1 Inclusion criteria

(1) Study Types: RCTs. (2) Participants: Adult women (age ≥18 years) with a confirmed diagnosis of PCOS and obesity, as defined by study-specific criteria. (3) Interventions: The experimental group received oral THM as an add-on to conventional therapy, with a clearly documented botanical composition. The control group received conventional pharmacotherapy combined with the same background therapy. (4) Outcome Measures: Primary outcomes were clinical efficacy rate and clinical pregnancy rate. Secondary outcomes included Homeostatic Model Assessment of Insulin Resistance (HOMA-IR), body mass index (BMI), total testosterone (TT), and the luteinizing hormone/follicle-stimulating hormone (LH/FSH) ratio.

#### 2.2.2 Exclusion criteria

(1) Studies where the experimental group received any non-oral THM therapy; (2) Studies using patented THM formulas with undisclosed composition; (3) Ambiguous author details or data sources; (4) Non-randomized trials, reviews, case reports, or animal studies.

### 2.3 Data processing and methodological quality assessment

Two researchers independently extracted the following data from each included study: title, first author, publication year, sample size, diagnostic criteria for PCOS and obesity, interventions (including the specific botanical composition of the THM formulas), treatment duration, and all reported outcome measures. To ensure transparent reporting of THM formulas, the ConPhyMP guidelines were followed. (Heinrich et al., 2022). For accurate taxonomic identification, the scientific nomenclature of all botanical materials was verified and standardized using authoritative databases, including Medicinal Plant Names Services, Species Fungorum, the Global Biodiversity Information Facility (GBIF), and the Integrated Taxonomic Information System (ITIS). Additional specialized databases were consulted as needed to confirm the nomenclature of less common organismal groups. Corresponding authors were contacted for missing summary statistics, and any unrecoverable data were excluded from the synthesis.

The methodological quality of the included studies was assessed independently by two researchers using the revised Cochrane risk-of-bias tool (RoB 2.0) for randomized trials (Sterne et al., 2019). Any discrepancies arising during data extraction or quality assessment were resolved through consensus discussion between the reviewers or, when necessary, by arbitration from a senior researcher.

### 2.4 Statistical analysis

#### 2.4.1 Evidence synthesis

All meta-analyses were performed using Stata 15.1. Odds ratios (OR) were calculated for dichotomous outcomes and standardized mean differences (SMD) for continuous variables. Heterogeneity was assessed using Cochran’s Q test and I^2^ statistics. A fixed-effects model was applied when I^2^ ≤ 50% and P ≥ 0.10; otherwise, a random-effects model was used. For the random-effects model, the between-study variance (τ^2^) was estimated using the DerSimonian–Laird (DL) method. For outcomes with substantial heterogeneity and the impact of intervention characteristics, pre-specified subgroup analyses were conducted based on the formulation type of THM (decoction, granule, other), treatment duration (≤12 weeks, >12 weeks) and PCOS diagnostic criteria. Publication bias was evaluated using funnel plots, supplemented by Egger’s regression test and trim-and-fill adjustment with a maximum of 10 iterations (Lin and Chu, 2018; Rodgers and Pustejovsky, 2021).

#### 2.4.2 Association rule mining of botanical drug formulas

To systematically characterize the compositional profiles of the THM formulas included in the meta-analysis, we performed association rule mining to identify recurrent botanical drug combinations and patterns of co-occurrence. The analysis was implemented using the *Apriori* algorithm in IBM SPSS Modeler 18.0 (Wu et al., 2021; Zhang S. et al., 2023). In this analysis, each included RCT was treated as a distinct “transaction.” For multi-arm RCTs, each treatment arm receiving a distinct herbal intervention was considered an independent transaction. Key analysis thresholds were predefined to balance the discovery of robust, interpretable patterns against the exclusion of spurious associations, guided by common practices in herbal medicine data mining (Agrawal et al., 1993; Tan et al., 2019). The minimum support was set at 10% to focus on sufficiently frequent botanical drug combinations (Zhang S. et al., 2023). A minimum confidence of 80% was required to ensure high predictive reliability for the derived rules (Wu et al., 2021). A lift value greater than 1 was used to identify meaningful, non-random associations. The maximum antecedent count was limited to 2 herbs to prioritize concise and clinically interpretable botanical drug pairs or triplets (Tan et al., 2019). These parameter choices aimed to highlight core combinations without generating an excessive number of rules. Finally, a network diagram was generated using Cytoscape 3.10.1 to visualize co-occurrence patterns.

### 2.5 Assessment of evidence certainty

The certainty of evidence for each outcome was appraised using the Grading of Recommendations Assessment, Development and Evaluation (GRADE) framework via the GRADEpro GDT tool. Evidence was categorized as high, moderate, low, or very low based on study limitations, inconsistency, indirectness, imprecision, and publication bias.

## 3 Results

### 3.1 Results of literature retrieval

**FIGURE 1:**
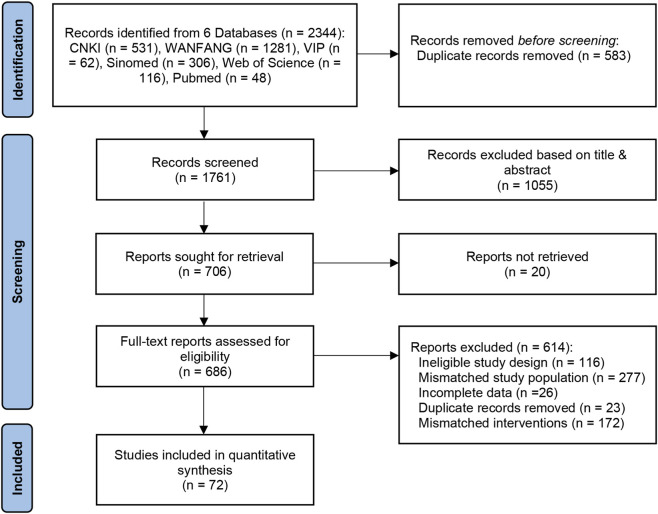
Literature screening flowchart.

A total of 2,344 records were initially retrieved from the six databases. After removing duplicates and applying the eligibility criteria, 72 RCTs were finally included. The detailed screening process is presented in Figure 1.

### 3.2 Study characteristics and quality assessment

The 72 included RCTs, published between 2009 and 2024, enrolled a total of 5,308 patients. Sample sizes of individual studies ranged from 36 to 126, and treatment durations varied from 2 to 24 weeks. Among the 72 included studies, BMI was reported in 57 studies (Feng, 2009; Jiao et al., 2013; Lu, 2013; Song et al., 2015; Wang et al., 2015; Yin et al., 2015; Huang, 2016; Huang, 2019; Huang et al., 2016; Song, 2016; Fu and Li, 2017; Lin et al., 2017; Liu, 2017; Liu, 2023b; Liu, 2023a; Xu et al., 2017; Ye et al., 2017; Zhou, 2017; Zhou, 2021; Xie, 2018; Yang and Yan, 2018; Chen, 2019b; Di, 2019; Li et al., 2019; Yang, 2019; Zhang et al., 2019; Zhang et al, 2022; Zhang H. et al., 2023; 2024; Duan, 2020; Fu, 2020; Fu, 2023; Fu, 2024; He, 2020; He, 2021; Hou, 2020; Shi, 2020; Ben, 2021; Fang, 2021; Ge, 2021; Li, 2021; Lin, 2021; Ren, 2021; Tang, 2021; Tang, 2022; Wang, 2021; Xu, 2021; Cheng et al., 2022; Cui, 2022; Shan and Gao, 2022; Wei, 2022; Shen, 2023; Shen et al., 2023; Zhao, 2023; Zhong et al., 2023; Chen et al., 2024; Fang and Zhu, 2024; Yu, 2024), TT in 56 studies (Feng, 2009; Jiao et al., 2013; Lu, 2013; Song et al., 2015; Huang et al., 2016; Song, 2016; Fu and Li, 2017; Guo, 2017; Lin et al., 2017; Liu, 2017; Liu, 2023b; Liu, 2023a; Xu et al., 2017; Ye et al., 2017; Zhou, 2017; Zhou, 2021; Bai et al., 2018; Liu and Zhu, 2018; Xie, 2018; Zhang, 2018; Chen, 2019b; Chen, 2019a; Deng, 2019; Di, 2019; Huang, 2019; Li et al., 2019; Li et al, 2020; Yang, 2019; Zhang et al., 2019; Zhang et al, 2022; Zhang H. et al., 2023; Zhang et al, 2024; Duan, 2020; Fu, 2020; Fu, 2024; He, 2020; He, 2021; Hou, 2020; Lin, 2020; 2021; Wang and Tian, 2020; Ben, 2021; Fan and Lin, 2021; Fang, 2021; Ge, 2021; Ren, 2021; Xu, 2021; Cheng et al., 2022; Sun, 2022; Tang, 2022; Wei, 2022; Shen, 2023; Shen et al., 2023; Zhao, 2023; Chen et al., 2024; Fang and Zhu, 2024), and the clinical efficacy rate in 55 studies (Jiao et al., 2013; Lu, 2013; Song et al., 2015; Wang et al., 2015; Huang, 2016; 2019; Huang et al., 2016; Song, 2016; Guo, 2017; Liu, 2017; 2023b; 2023a; Xu et al., 2017; Ye et al., 2017; Bai et al., 2018; Xie, 2018; Yang and Yan, 2018; Zhang, 2018; Chen, 2019a; Deng, 2019; Di, 2019; Li et al., 2019; Li et al, 2020; Yang, 2019; Zhang et al., 2019; Zhang et al, Zhang et al, 2022; Zhang et al, 2024; Duan, 2020; Fu, 2020; Fu, 2023; He, 2020; He, 2021; Hou, 2020; Lin, 2020; 2021; Wang and Tian, 2020; Ben, 2021; Fang, 2021; Ge, 2021; Li, 2021; Ren, 2021; Tang, 2021; Tang, 2022; Wang, 2021; Xu, 2021; Zhou, 2021; Zhou et al., 2021; Cheng et al., 2022; Shan and Gao, 2022; Wei, 2022; Zeng et al., 2022; Shen, 2023; Zhao, 2023; Zhong et al., 2023; Fang and Zhu, 2024), which were the most frequently assessed outcomes. Furthermore, the LH/FSH ratio was documented in 33 studies (Jiao et al., 2013; Lu, 2013; Huang et al., 2016; Song, 2016; Lin et al., 2017; Liu, 2017; 2023b; 2023a; Xu et al., 2017; Zhou, 2017; 2021; Chen, 2019b; Deng, 2019; Di, 2019; Yang, 2019; Duan, 2020; Fu, 2020; Fu, 2023; Fu, 2024; Hou, 2020; Wang and Tian, 2020; Ben, 2021; Fang, 2021; Ge, 2021; Lin, 2021; Tang, 2021; Tang, 2022; Wang, 2021; Xu, 2021; Cheng et al., 2022; Zhang et al., 2022; Shen, 2023; Chen et al., 2024), and the HOMA-IR in 36 studies (Feng, 2009; Jiao et al., 2013; Song et al., 2015; Huang et al., 2016; Lin et al., 2017; Liu, 2017; Liu, 2023b; Xu et al., 2017; Ye et al., 2017; Zhou, 2017; Zhou, 2021; Bai et al., 2018; Liu and Zhu, 2018; Deng, 2019; Huang, 2019; Yang, 2019; Duan, 2020; Fu, 2020; Fu, 2024; He, 2020; 2021; Hou, 2020; Li et al., 2020; Fang, 2021; Ge, 2021; Lin, 2021; Ren, 2021; Cui, 2022; Jiang et al., 2022; Tang, 2022; Wei, 2022; Shen, 2023; Zhang H. et al., 2023; Zhong et al., 2023; Chen et al., 2024; Yu, 2024). For reproductive outcomes, the clinical pregnancy rate was reported in 9 studies (Yin et al., 2015; Liu, 2017; Zhang, 2018; Chen, 2019b; Hou, 2020; Lin, 2020; Shi, 2020; Fan and Lin, 2021; Cui, 2022). The basic characteristics of the included studies are summarized in Table 1. The ConPhyMP checklist assessing the reporting completeness of each THM formula is provided in the Supplementary Data Sheet.

**TABLE 1:** Characteristics of the included studies.

Study	Region	Sample size (E/C)	Diagnostic criteria	Experimental group	Control group	Duration	Formulation type	Outcomes
Fu, Z (2024)	China	34/34	Chinese diagnostic criteria	Erxian qiling decoction + ethinylestradiol and cyproterone acetate tablets	Ethinylestradiol and cyproterone acetate tablets	12 weeks	Decoction	(3) (4) (5) (6)
Fang and Zhu (2024)	China	44/44	Chinese diagnostic criteria	Modified cangfu daotan decoction + ethinylestradiol and cyproterone acetate tablets	Ethinylestradiol and cyproterone acetate tablets	12 weeks	Decoction	(1) (4) (5)
Yu, T (2024)	China	40/41	Rotterdam criteria	Gexia zhuyu decoction + metformin	Metformin	12 weeks	Decoction	(3) (4)
Chen et al. (2024)	China	48/48	Chinese diagnostic criteria	Huazhuo jiedu decoction + drospirenone and ethinylestradiol tablets	Drospirenone and ethinylestradiol tablets	12 weeks	Decoction	(3) (4) (5) (6)
Zhang et al. (2024)	China	40/40	Chinese diagnostic criteria	Self-formulated prescription + ethinylestradiol and cyproterone acetate Tablets + Metformin	Ethinylestradiol and cyproterone acetate tablets + metformin	24 weeks	Other	(1) (4) (5)
Shen, J (2023)	China	30/30	Chinese diagnostic criteria	Dachaihu decoction combined with fangji huangqi decoction + metformin	Metformin	12 weeks	Decoction	(4) (5)
Zhong et al. (2023)	China	63/63	Chinese diagnostic criteria	Fangfeng tongsheng decoction + metformin	Metformin	12 weeks	Decoction	(1) (3) (4)
Shen, Y (2023)	China	36/36	Chinese diagnostic criteria	Qutan lishi decoction + metformin	Metformin	12 weeks	Decoction	(1) (3) (4) (5) (6)
Liu, J (2023b)	China	40/40	Chinese diagnostic criteria	Self-formulated prescription + metformin	Metformin	12 weeks	Decoction	(1) (3) (4) (5) (6)
Fu, C (2023)	China	32/32	Chinese diagnostic criteria	Yinang jianzhi decoction + dydrogesterone	Dydrogesterone	12 weeks	Decoction	(1) (6) (4)
Liu, J (2023a)	China	44/44	Rotterdam criteria	Modified danxi zhishitan decoction + ethinylestradiol and cyproterone acetate tablets	Ethinylestradiol and cyproterone acetate tablets	12 weeks	Decoction	(1) (4) (5) (6)
Zhao, C (2023)	China	35/35	Rotterdam criteria	Bushen huatan decoction + metformin	Metformin	12 weeks	Decoction	(1) (4) (5)
Zhang S. et al. (2023)	China	30/30	Rotterdam criteria	Qigong decoction + metformin	Metformin	12 weeks	Decoction	(3) (4) (5)
Shan and Gao (2022)	China	60/60	Chinese diagnostic criteria	Cangfu daotan decoction + metformin	Metformin	12 weeks	Decoction	(1) (2) (4)
Zhang et al. (2022)	China	60/59	Rotterdam criteria	Cangfu daotan decoction + ethinylestradiol and cyproterone acetate tablets	Metformin + ethinylestradiol and cyproterone acetate tablets	12 weeks	Decoction	(1) (4) (5) (6)
Tang, J (2022)	China	43/43	Chinese diagnostic criteria	Ditan zhuyu decoction + metformin	Metformin	12 weeks	Decoction	(1) (3) (4) (5) (6)
Jiang et al. (2022)	China	58/60	Rotterdam criteria	Gexia zhuyu decoction + metformin	Metformin	12 weeks	Decoction	(3)
Cui, M (2022)	China	30/30	Rotterdam criteria	Bushen huatan decoction + orlistat capsules	Orlistat capsules	12 weeks	Decoction	(3) (4) (7)
Wei, X (2022)	China	51/51	Chinese diagnostic criteria	Bushen huoxue decoction + metformin + ethinylestradiol and cyproterone acetate tablets	Metformin + ethinylestradiol and cyproterone acetate tablets	12 weeks	Decoction	(1) (3) (4) (5)
Sun, M (2022)	China	47/47	Chinese diagnostic criteria	Huoxue qushi bushen decoction + ethinylestradiol and cyproterone acetate tablets	Ethinylestradiol and cyproterone acetate tablets	12 weeks	Decoction	(5)
Zeng et al. (2022)	China	36/36	Chinese diagnostic criteria	Cupailuan decoction + clomifene citrate capsules	Clomifene citrate capsules	12 weeks	Decoction	(1)
Cheng et al. (2022)	China	30/30	Chinese diagnostic criteria	Fenxiao huoxue decoction + ethinylestradiol and cyproterone acetate tablets	Ethinylestradiol and cyproterone acetate tablets	12 weeks	Decoction	(1) (4) (5) (6)
Zhou, T (2021)	China	30/30	Chinese diagnostic criteria	Jianpi huatan decoction + metformin	Metformin	12 weeks	Decoction	(1)
Xu, Y (2021)	China	28/29	Rotterdam criteria	Modified huanglian wendan decoction + drospirenone and ethinylestradiol tablets	Drospirenone and ethinylestradiol tablets	12 weeks	Decoction	(1) (4) (5) (6)
Zhou, G (2021)	China	31/32	Rotterdam criteria	Qigong pills modified decotion + drospirenone and ethinylestradiol tablets (Ⅱ)	Drospirenone and ethinylestradiol tablets (Ⅱ)	12 weeks	Granula	(1) (3) (4) (5) (6)
Ren, Y (2021)	China	35/33	Chinese diagnostic criteria	Shoushen tiaojing decoction + metformin	Metformin	12 weeks	Granula	(1) (3) (4) (5)
Lin, Z (2021)	China	28/27	Chinese diagnostic criteria	Modified pingwei san + metformin	Metformin	12 weeks	Granula	(1) (3) (4) (5) (6)
Li, J (2021)	China	30/30	Chinese diagnostic criteria	Jianpi huatan decoction + metformin	Metformin	12 weeks	Decoction	(1) (4)
Ge, R (2021)	China	40/40	Chinese diagnostic criteria	Modified erchen decoction combined with gegen decoction + ethinylestradiol and cyproterone acetate tablets	Ethinylestradiol and cyproterone acetate tablets	12 weeks	Decoction	(1) (3) (4) (5) (6)
Tang, Y (2021)	China	37/37	Chinese diagnostic criteria	Yinang zhuyun decoction + ethinylestradiol and cyproterone acetate tablets	Ethinylestradiol and cyproterone acetate tablets	12 weeks	Decoction	(1) (4) (6)
Fang, S (2021)	China	30/30	Rotterdam criteria	Shenling baizhu powder + beinaglutide	Beinaglutide	12 weeks	Granula	(1) (3) (4) (5) (6)
Ben, Q (2021)	China	33/33	Chinese diagnostic criteria	Yishen xiaotan decoction + ethinylestradiol and cyproterone acetate tablets	Ethinylestradiol and cyproterone acetate tablets	12 weeks	Decoction	(1) (4) (5) (6)
Fan and Lin (2021)	China	30/30	Chinese diagnostic criteria	Modified cangfu daotan decoction + progesterone + letrozole	Progesterone + letrozole	12 weeks	Other	(5) (7)
Wang, Z (2021)	China	44/44	Chinese diagnostic criteria	Self-formulated prescription + liraglutide	Liraglutide	12 weeks	Decoction	(1) (4) (6)
He, X (2021)	China	40/40	Chinese diagnostic criteria	Jianpi bushen huoxue decoction + ethinylestradiol and cyproterone acetate tablets	Ethinylestradiol and cyproterone acetate tablets	12 weeks	Decoction	(1) (3) (4) (5)
Duan, X (2020)	China	30/30	Rotterdam criteria	Shiying yulin decoction + ethinylestradiol and cyproterone acetate tablets	Ethinylestradiol and cyproterone acetate tablets	12 weeks	Decoction	(1) (3) (4) (5) (6)
Fu, R (2020)	China	30/30	Chinese diagnostic criteria	Jianpi huatan decoction + metformin	Metformin	12 weeks	Granula	(1) (3) (4) (5) (6)
He, J (2020)	China	40/40	Rotterdam criteria	Jianpi bushen decoction + metformin	Metformin	12 weeks	Decoction	(1) (3) (4) (5)
Wang and Tian (2020)	China	40/40	Rotterdam criteria	Bushen quyu huatan decoction + ethinylestradiol and cyproterone acetate tablets	Ethinylestradiol and cyproterone acetate tablets	12 weeks	Other	(1) (5) (6)
Lin, H (2020)	China	40/40	Rotterdam criteria	Cangfu daotan decoction + ethinylestradiol and cyproterone acetate tablets	Ethinylestradiol and cyproterone acetate tablets	4 weeks	Decoction	(1) (5) (7)
Hou, X (2020)	China	32/32	Chinese diagnostic criteria	Bushen qutan Decoction + Metformin	Metformin	12 weeks	Decoction	(1) (3) (4) (5) (6) (7)
Shi, Q (2020)	China	38/38	Chinese diagnostic criteria	Bushen huatan decoction + clomifene citrate capsules	Clomifene citrate capsules	24 weeks	Decoction	(4) (7)
Li et al. (2020)	China	35/35	Chinese diagnostic criteria	Self-formulated prescription + drospirenone and ethinylestradiol tablets (Ⅱ)	Drospirenone and ethinylestradiol tablets (Ⅱ)	12 weeks	Decoction	(1) (3) (5)
Deng, X (2019)	China	30/30	Rotterdam criteria	Danggui dihuang decoction combined with taoren siwu decoction + metformin	Metformin	12 weeks	Granula	(1) (3) (5) (6)
Li et al. (2019)	China	52/52	Rotterdam criteria	Yishen huatan decoction + clomifene citrate capsules	Clomifene citrate capsules	12 weeks	Decoction	(1) (4) (5)
Chen, Y (2019b)	China	30/30	Chinese diagnostic criteria	Self-formulated prescription + clomifene citrate capsules	Clomifene citrate capsules	12 weeks	Granula	(4) (5) (6) (7)
Yang, Y (2019)	China	35/31	Rotterdam criteria	Xiaozhi decoction + metformin	Metformin	12 weeks	Decoction	(1) (3) (4) (5) (6)
Di, X (2019)	China	22/22/22	Rotterdam criteria	Cangfu daotan decoction + ethinylestradiol and cyproterone acetate tablets	Ethinylestradiol and cyproterone acetate tablets	12 weeks	Decoction	(1) (4) (5) (6)
Huang, C (2019)	China	30/30	Rotterdam criteria	Modified cangfu daotan decoction + drospirenone and ethinylestradiol tablets	Drospirenone and ethinylestradiol tablets	12 weeks	Decoction	(1) (2) (3) (4) (5)
Chen, Y (2019a)	China	48/48	Rotterdam criteria	Cangfu daotan decoction + ethinylestradiol and cyproterone acetate tablets	Ethinylestradiol and cyproterone acetate tablets	12 weeks	Decoction	(1) (5)
Zhang et al. (2019)	China	38/38	Rotterdam criteria	Modified cangfu daotan decoction + letrozole	Letrozole	24 weeks	Decoction	(1) (4) (5)
Bai et al. (2018)	China	38/37	Rotterdam criteria	Qihuang zengmin decoction + metformin	Metformin	12 weeks	Granula	(1) (3) (5)
Zhang, H (2018)	China	56/56	Chinese diagnostic criteria	Modified cangfu daotan decoction + clomifene citrate capsules	Clomifene citrate capsules	12 weeks	Decoction	(1) (2) (5) (7)
Yang and Yan (2018)	China	60/60	Chinese diagnostic criteria	Modified guizhi fuling pill combined with danggui shaoyao decoction + metformin	Metformin	24 weeks	Granula	(1) (4)
Xie (2018)	China	40/40	Rotterdam criteria	Bushen shugan huayu qutan decoction + metformin	Metformin	12 weeks	Decoction	(1) (4) (5)
Liu and Zhu (2018)	China	49/49	Rotterdam criteria	Self-formulated prescription + metformin	Metformin	12 weeks	Decoction	(3) (5)
Xu et al. (2017)	China	29/25	Rotterdam criteria	Tanzhixiao granula + metformin	Metformin	12 weeks	Granula	(1) (3) (4) (5) (6)
Fu and Li (2017)	China	22/20	Rotterdam criteria	Modified cangfu daotan decoction + ethinylestradiol and cyproterone acetate tablets	Ethinylestradiol and cyproterone acetate tablets	12 weeks	Decoction	(4) (5)
Zhou, D (2017)	China	30/30	Rotterdam criteria	Heqi powder + metformin	Metformin	12 weeks	Granula	(3) (4) (5) (6)
Liu, M (2017)	China	20/20	Chinese diagnostic criteria	Bushen huatan decoction + metformin	Metformin	12 weeks	Decoction	(1) (3) (4) (5) (6) (7)
Ye et al. (2017)	China	32/30	Rotterdam criteria	Sanhuang decoction + metformin	Metformin	12 weeks	Granula	(1) (3) (4) (5)
Guo, R (2017)	China	40/40	Rotterdam criteria	Cangfu daotan decoction + clomifene citrate capsules	Clomifene citrate capsules	12 weeks	Decoction	(1) (5)
Lin et al. (2017)	China	24/24	Chinese diagnostic criteria	Huatan tongmai decoction + metformin	Metformin	12 weeks	Decoction	(3) (4) (5) (6)
Song, C (2016)	China	30/30	Rotterdam criteria	Jianpi lishi yiqi yangyin decoction + metformin	Metformin	12 weeks	Granula	(1) (4) (5) (6)
Huang, C (2016)	China	40/40	Rotterdam criteria	Buqi huatan xingqi decoction + metformin	Metformin	12 weeks	Decoction	(1) (3) (4) (5) (6)
Huang, J (2016)	China	30/30	Rotterdam criteria	Self-formulated prescription + progesterone injection	Progesterone injection	2 weeks	Granula	(1)
Song et al. (2015)	China	30/30	Rotterdam criteria	Yishen huatan decoction + metformin	Metformin	12 weeks	Decoction	(1) (3) (4) (5)
Wang et al. (2015)	China	22/24	Rotterdam criteria	Self-formulated prescription + letrozole	Letrozole	12 weeks	Decoction	(1) (4)
Yin et al. (2015)	China	32/32	Rotterdam criteria	Bushen huatan decoction + ethinylestradiol and cyproterone acetate tablets	Ethinylestradiol and cyproterone acetate tablets + letrozole	16 weeks	Decoction	(4) (7)
Lu, L (2013)	China	30/30	Rotterdam criteria	Chushi huatan decoction + metformin	Metformin	12 weeks	Granula	(1) (4) (5) (6)
Jiao et al. (2013)	China	26/10	Rotterdam criteria	Zaoshi huatan bushen decoction + metformin	Metformin	12 weeks	Granula	(1) (3) (4) (5) (6)
Feng, C (2009)	China	50/50	Rotterdam criteria	Modified erchen decoction + ethinylestradiol and cyproterone acetate tablets + metformin	Ethinylestradiol and cyproterone acetate tablets + metformin	12 weeks	Decoction	(3) (4) (5)

Outcomes are coded numerically as follows: (1) Clinical Efficacy Rate; (2) HOMA-IR; (3) BMI; (4) TT; (5) LH/FSH, ratio; (6) Clinical Pregnancy Rate.

### 3.3 Risk of bias

**FIGURE 2:**
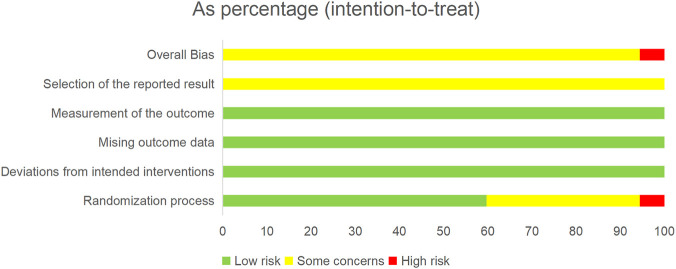
Risk of bias summary.

Assessment using the Cochrane RoB 2.0 tool revealed significant concerns regarding the reporting of methodological rigor in the included studies. While all studies claimed randomization, the reporting was generally inadequate: only one study was double-blinded, five employed inappropriate randomization methods, and thirty-two mentioned “randomization” without providing details. The absence of prospective protocol registration for all studies led to a judgment of “some concerns” or “high risk” of bias in the randomization process (Domain 1) and in the selection of the reported result (Domain 5). A summary of the risk of bias assessment is presented in Figure 2, with the full evaluation available in Supplementary Table S2.

### 3.4 Meta-analysis

#### 3.4.1 Clinical efficacy rate

**FIGURE 3:**
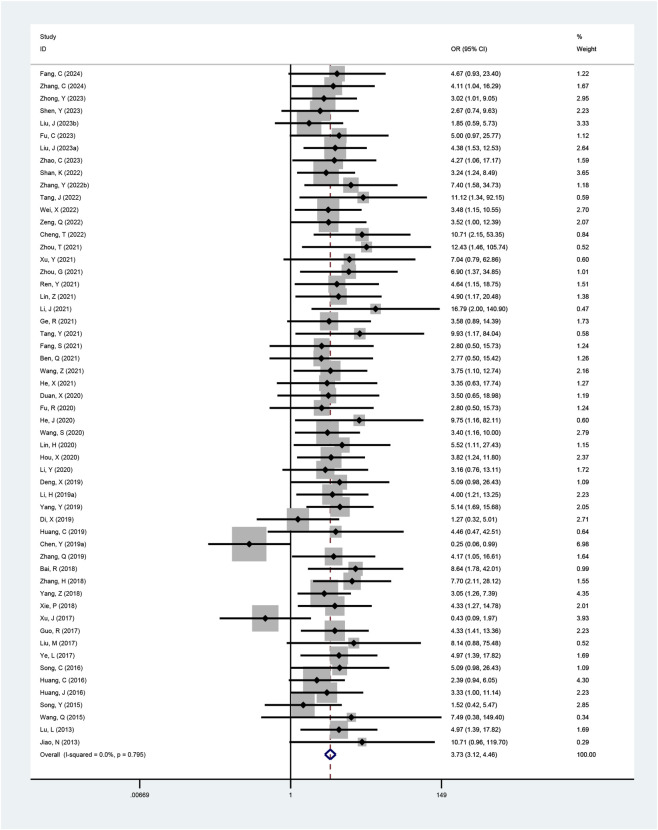
Meta-analysis results of clinical efficacy rate.

Fifty-five studies reported the clinical efficacy rate. Due to low heterogeneity among the studies (I^2^ = 0%, p = 0.795), a fixed-effects model was applied for the meta-analysis. The results demonstrated that the combined therapy significantly improved clinical efficacy rates compared to conventional pharmacotherapy alone (OR = 3.73, 95% CI: 3.12 to 4.46, p < 0.001; Figure 3).

#### 3.4.2 Clinical pregnancy rate

**FIGURE 4:**
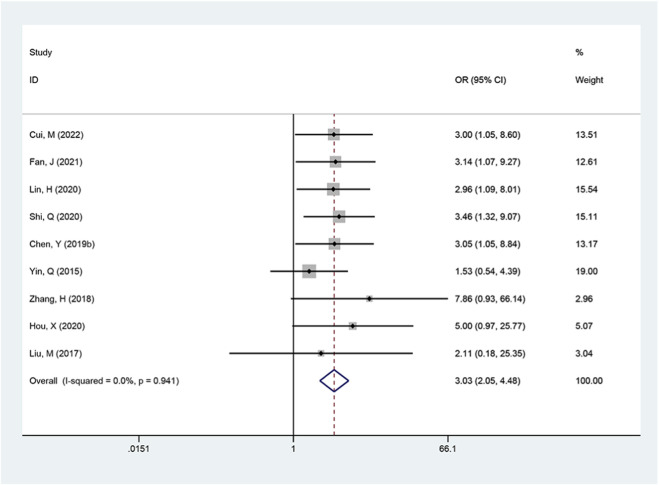
Meta-analysis results of clinical pregnancy rate.

Nine studies provided data on clinical pregnancy rate. Heterogeneity testing indicated no significant heterogeneity across studies (I^2^ = 0%, p = 0.941), and the fixed-effect model was applied. Meta-analysis showed that the combined therapy markedly increased the clinical pregnancy rate in obese women with PCOS (OR = 3.03, 95% CI: 2.05 to 4.48, p < 0.001; Figure 4).

#### 3.4.3 HOMA-IR

**FIGURE 5:**
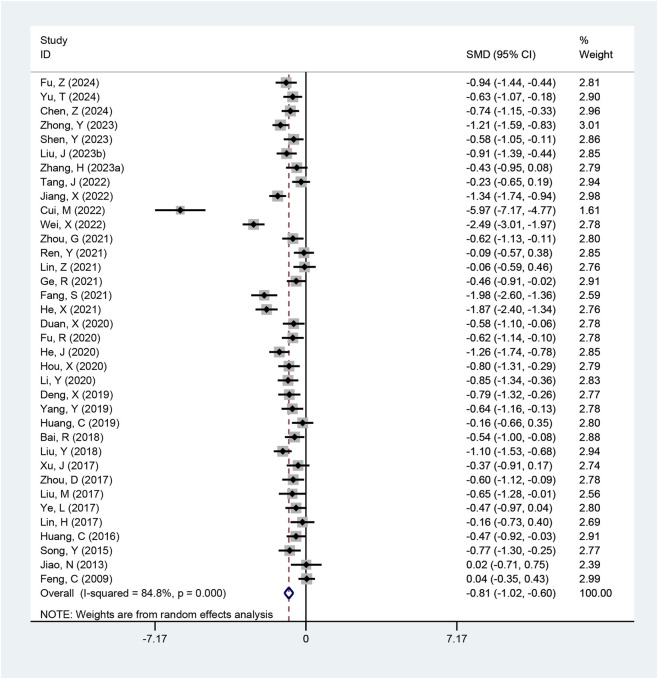
Meta-analysis results of HOMA-IR.

Thirty-six studies reported HOMA-IR values. Substantial heterogeneity was observed among studies (I^2^ = 84.8%, p < 0.001; τ^2^ = 0.35), leading to the use of a random-effects model for pooling. The analysis indicated that the combined therapy was superior to conventional medication in reducing HOMA-IR (SMD = −0.81, 95% CI: −1.02 to −0.60, p < 0.001; Figure 5). Subgroup analyses were consistent (Table 2; Supplementary Figures S1, S2).

**TABLE 2:** Exploratory subgroup analyses to investigate sources of heterogeneity.

Outcome	Subgroup	Studies, n	SMD (95% CI)	P-value	Heterogeneity (I^2^, τ^2^, P-value)
LH/FSH ratio					
​	**Overall**	**33**	**−0.88 (-1.05, -0.70)**	**<0.001**	**73.1%, τ** ^**2**^ **= 0.18, <0.001**
​	By diagnostic criteria	​	​	​	​
​	Chinese diagnostic criteria	17	−0.89 (−1.08, −0.70)	<0.001	59.5%, τ^2^ = 0.10, = 0.001
​	Rotterdam criteria	16	−0.86 (−1.16, −0.56)	<0.001	81.0%, τ^2^ = 0.30, <0.001
​	By formulation type	​	​	​	​
​	Decoction	21	−0.90 (−1.11, −0.68)	<0.001	74.2%, τ^2^ = 0.18, <0.001
​	Granula	12	−0.82 (−1.15, −0.49)	<0.001	74.8%, τ^2^ = 0.24, <0.001
HOMA-IR					
​	**Overall**	**36**	**−0.81 (-1.02, -0.60)**	**<0.001**	**84.8%, τ** ^**2**^ **= 0.35, <0.001**
​	By diagnostic criteria	​	​	​	​
​	Chinese diagnostic criteria	15	−0.76 (−1.08, −0.44)	<0.001	84.0%, τ^2^ = 0.33, <0.001
​	Rotterdam criteria	21	−0.85 (−1.14, −0.56)	<0.001	85.9%, τ^2^ = 0.39, <0.001
​	By formulation type	​	​	​	​
​	Decoction	25	−0.93 (−1.20, −0.66)	<0.001	87.3%, τ^2^ = 0.41, <0.001
​	Granula	11	−0.55 (−0.84, −0.27)	<0.001	67.7%, τ^2^ = 0.15, = 0.001
BMI					
​	**Overall**	**55**	**−0.95 (-1.09, -0.81)**	**<0.001**	**77.1%, τ** ^**2**^ **= 0.22, <0.001**
​	By diagnostic criteria	​	​	​	​
​	Chinese diagnostic criteria	25	−0.94 (−1.14, −0.75)	<0.001	76.2%, τ^2^ = 0.19, <0.001
​	Rotterdam criteria	30	−0.96 (−1.16, −0.76)	<0.001	78.5%, τ^2^ = 0.25, <0.001
​	By treatment duration	​	​	​	​
​	≤12 weeks	50	−1.00 (−1.15, −0.85)	<0.001	77.5%, τ^2^ = 0.22, <0.001
​	>12 weeks	5	−0.53 (−0.85, −0.21)	= 0.001	46.2%, τ^2^ = 0.06, = 0.115
​	By formulation type	​	​	​	​
​	Decoction	42	−0.98 (−1.15, −0.81)	<0.001	79.9%, τ^2^ = 0.25, <0.001
​	Granula	12	−0.90 (−1.14, −0.66)	<0.001	61.9%, τ^2^ = 0.11, = 0.002
​	Other	1	−0.59 (−1.04, −0.15)	= 0.009	NA
TT					
​	**Overall**	**56**	**−0.90 (-1.10, -0.69)**	**<0.001**	**89.3%, τ** ^**2**^ **= 0.54, <0.001**
​	By diagnostic criteria	​	​	​	​
​	Chinese diagnostic criteria	24	−1.11 (−1.46, −0.76)	<0.001	91.3%, τ^2^ = 0.69, <0.001
​	Rotterdam criteria	32	−0.74 (−0.98, −0.50)	<0.001	86.7%, τ^2^ = 0.42, <0.001
​	By treatment duration	​	​	​	​
​	≤12 weeks	54	−0.88 (−1.09, −0.68)	<0.001	89.2%, τ^2^ = 0.54, <0.001
​	>12 weeks	2	−1.26 (−2.62, −0.10)	= 0.068	93.2%, τ^2^ = 0.89, <0.001
​	By formulation type	​	​	​	​
​	Decoction	39	−0.84 (−1.05, −0.63)	<0.001	85.6%, τ^2^ = 0.38, <0.001
​	Granula	14	−0.72 (−1.16, −0.28)	= 0.004	90.7%, τ^2^ = 0.64, <0.001
​	Other	3	−2.59 (−4.34, −0.84)	= 0.001	96.1%, τ^2^ = 0.29, <0.001

Bold values indicate summary effect estimates (SMD) with statistically significant P-values (< 0.05).

#### 3.4.4 BMI

**FIGURE 6:**
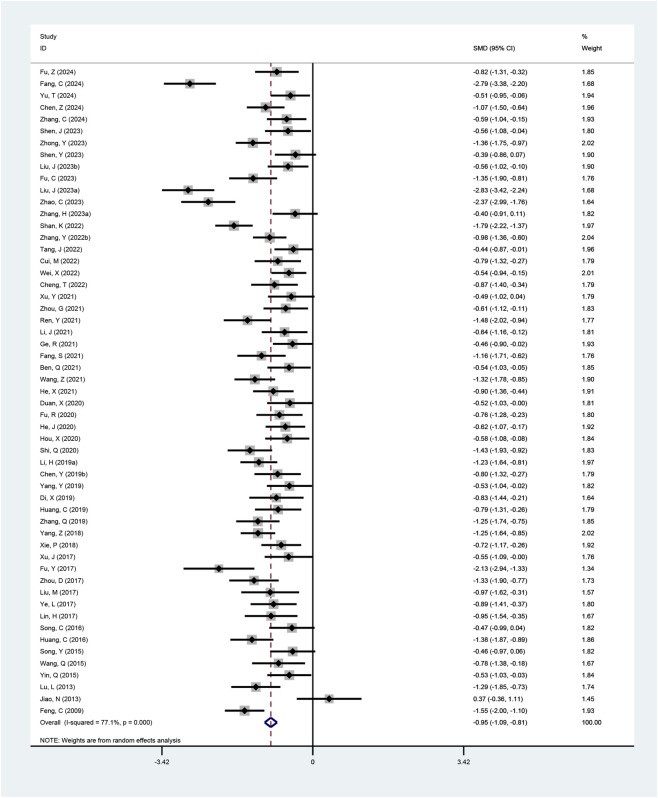
Meta-analysis results of BMI.

Fifty-seven studies reported BMI outcomes, with two excluded from the pooled analysis due to incompatible data formats (Feng, 2009; Jiao et al., 2013). Given the high heterogeneity among studies (I^2^ = 77.1%, p < 0.001; τ^2^ = 0.22), the random-effects model was selected. The results showed that the combined therapy achieved a greater reduction in BMI than conventional treatment (SMD = −0.95, 95% CI: −1.09 to −0.81, p < 0.001; Figure 6), with subgroup analyses corroborating this result (Table 2; Supplementary Figures S3–S5).

#### 3.4.5 TT

**FIGURE 7:**
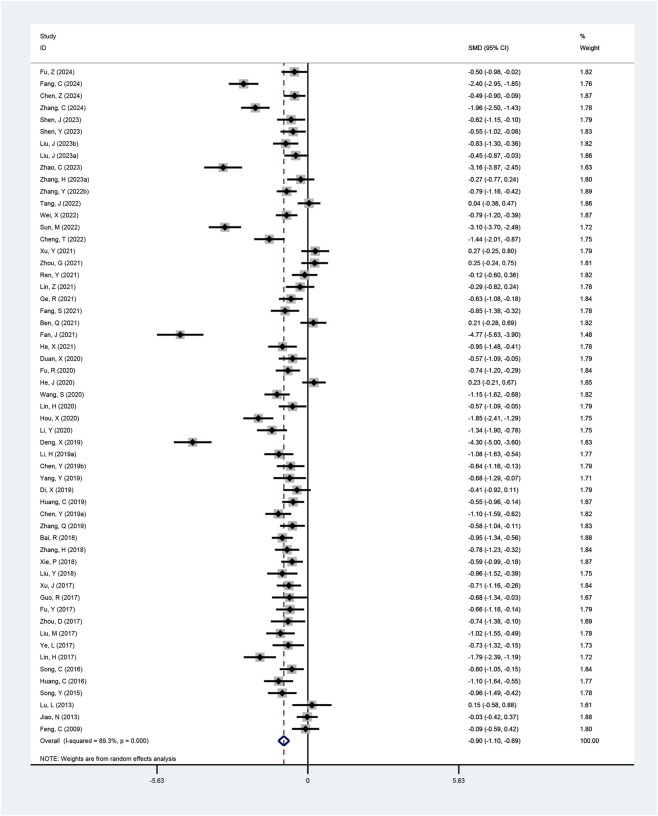
Meta-analysis results of TT.

Fifty-six studies reported total testosterone levels. Significant heterogeneity was detected (I^2^ = 89.3%, p < 0.001; τ^2^ = 0.54), prompting the use of a random-effects model. The pooled results indicated a more pronounced reduction in total testosterone with the combined therapy (SMD = −0.90, 95% CI: −1.10 to −0.69, p < 0.001; Figure 7), and subgroup analyses confirmed this finding (Table 2; Supplementary Figures S6–S8).

#### 3.4.6 LH/FSH ratio

**FIGURE 8:**
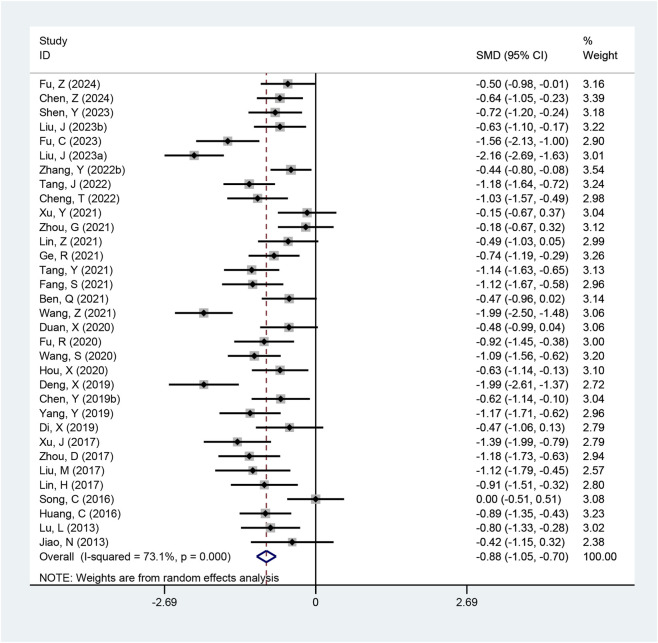
Meta-analysis results of LH/FSH ratio.

Thirty-three studies evaluated the LH/FSH ratio. Marked heterogeneity was present (I^2^ = 73.1%, p < 0.001; τ^2^ = 0.18), so a random-effects model was applied. The meta-analysis suggested that the combined therapy was more effective in improving the LH/FSH ratio (SMD = −0.88, 95% CI: −1.05 to −0.70, p < 0.001; Figure 8). Subgroup analysis based on diagnostic criteria produced similar conclusions (Table 2; Supplementary Figures S9, S10).

#### 3.4.7 Sensitivity analysis

Leave-one-out sensitivity analyses were conducted for all outcome indicators. The results confirmed that the pooled effect sizes remained stable after sequentially excluding individual study, indicating robust findings across all meta-analyses (Supplementary Figure S11).

#### 3.4.8 Publication bias

For outcomes with at least 10 included studies, publication bias was assessed using funnel plots (Supplementary Figure S12) and Egger’s linear regression test (Supplementary Figure S13). Significant bias was detected for clinical efficacy rate (Egger’s test P = 0.007), total testosterone (P < 0.001), and HOMA-IR (P = 0.035). Trim-and-fill analysis was subsequently applied to these outcomes (Supplementary Figure S14), which showed that the adjusted effect estimates remained statistically significant for clinical efficacy rate (OR = 3.05, 95% CI 2.57–3.61, P < 0.001), total testosterone (SMD = −1.20, 95% CI −1.42 to −0.98, P < 0.001), and HOMA-IR (SMD = −1.06, 95% CI −1.27 to −0.84, P < 0.001). In contrast, no significant publication bias was observed for BMI (P = 0.332) or LH/FSH ratio (P = 0.097). Overall, these findings suggest that the main conclusions are unlikely to be substantially influenced by publication bias.

#### 3.4.9 Certainty of the evidence

According to the GRADE assessment, the certainty of evidence for the reported outcomes varied from moderate to low (Supplementary Table S3). The evidence for clinical pregnancy rate was rated as moderate, while the evidence for other outcomes, including clinical efficacy rate, BMI, TT, LH/FSH ratio, and HOMA-IR, was rated as low. Downgrading was primarily due to concerns regarding risk of bias in the included studies, inconsistency among results for certain outcomes, and suspected publication bias.

### 3.5 Data mining analysis

#### 3.5.1 Frequency of botanical drug use

Across the included studies, a total of 137 distinct botanical drugs were utilized, with a cumulative frequency of 891 applications. The complete list of constituent botanical drugs is detailed in Supplementary Table S4. Table 3 presents the ten most frequently used botanical drugs, with usage frequencies ranging from 36.11% to 69.44%. Among these, five botanical drugs were employed in over 50% of the prescriptions: *Poria cocos* (Schw.) Wolf [Polyporaceae, *Poria*] (69.44%), *Citrus reticulata* Blanco [Rutaceae, *Citri Reticulatae Pericarpium*] (62.50%), *Atractylodes lancea* (Thunb.) DC. [Asteraceae, *Atractylodis Rhizoma*] (56.94%), *Angelica sinensis* (Oliv.) Diels [Apiaceae, *Angelicae Sinensis Radix*] (55.56%), and *Cyperus rotundus* L. [Cyperaceae, *Cyperi Rhizoma*] (52.78%).

**TABLE 3:** Top 10 most frequently used botanical drugs.

Rank	Botanical drug (scientific name)	Frequency of utilization	Relative frequency (%)
1	*Poria cocos* (schw.) Wolf [Polyporaceae, *Poria*]	50	69.44%
2	*Citrus reticulata* Blanco [Rutaceae, *Citri Reticulatae Pericarpium*]	45	62.50%
3	*Atractylodes lancea* (Thunb.) DC. [asteraceae, *Atractylodis Rhizoma*]	41	56.94%
4	*Angelica sinensis* (oliv.) Diels [Apiaceae, *Angelicae Sinensis Radix*]	40	55.56%
5	*Cyperus rotundus* L. [Cyperaceae, *Cyperi Rhizoma*]	38	52.78%
6	*Glycyrrhiza uralensis* fisch. [Fabaceae, *Glycyrrhizae Radix et Rhizoma*]	35	48.61%
7	*Epimedium brevicornu* Maxim. [Berberidaceae, *Epimedii Folium*]	30	41.67%
8	*Conioselinum anthriscoides* (H.Boissieu) pimenov and kljuykov [apiaceae, *Ligustici Rhizoma et Radix*]	29	40.28%
9	*Cuscuta australis* R.Br. [convolvulaceae, *Cuscutae Semen*]	27	37.50%
10	*Citrus × aurantium *L. [Rutaceae, *Aurantii Fructus*]	26	36.11%

#### 3.5.2 Association rule mining

Association rule mining on the included formulas generated a total of 231 rules. The top 10 rules, ranked in descending order by support, are listed in Table 4. The rule with the highest support (62.50%) was {*C. reticulata* Blanco [Rutaceae, *Citri Reticulatae Pericarpium*]} => {*P. cocos* (Schw.) Wolf [Polyporaceae, *Poria*]}, with a confidence of 82.22%. Furthermore, the rule {*A. lancea* (Thunb.) DC. [Asteraceae, *Atractylodis Rhizoma*], *C. rotundus* L. [Cyperaceae, *Cyperi Rhizoma*]} => {*C. reticulata* Blanco [Rutaceae, *Citri Reticulatae Pericarpium*]} achieved both the highest confidence (96.30%) and the highest lift value (1.54) among all rules.

**TABLE 4:** **Top 10 association rules for botanical drug combinations**.

Rank	Consequent	Antecedent	Support (%)	Confidence (%)	Lift
1	*Poria cocos* (schw.) Wolf [Polyporaceae, *Poria*]	*Citrus reticulata* Blanco [Rutaceae, *Citri Reticulatae Pericarpium*]	62.50	82.22	1.18
2	*Citrus reticulata Blanco [*Rutaceae*, Citri Reticulatae Pericarpium]*	*Atractylodes lancea (Thunb.) DC. [*Asteraceae*, Atractylodis Rhizoma]*	56.94	80.49	1.29
3	*Poria cocos* (schw.) Wolf [Polyporaceae, *Poria*]	*Atractylodes lancea (Thunb.) DC. [*Asteraceae*, Atractylodis Rhizoma] and Citrus reticulata Blanco [*Rutaceae*, Citri Reticulatae Pericarpium]*	45.83	81.82	1.18
4	*Citrus reticulata* Blanco [Rutaceae, *Citri Reticulatae Pericarpium*]	*Atractylodes lancea (Thunb.) DC. [*Asteraceae*, Atractylodis Rhizoma] and Poria cocos (Schw.) Wolf [*Polyporaceae*, Poria]*	43.06	87.10	1.39
5	*Atractylodes lancea (Thunb.) DC. [*Asteraceae*, Atractylodis Rhizoma]*	*Cyperus rotundus L. [*Cyperaceae*, Cyperi Rhizoma] and Citrus reticulata Blanco [*Rutaceae*, Citri Reticulatae Pericarpium]*	41.67	86.67	1.52
6	*Atractylodes lancea (Thunb.) DC. [*Asteraceae*, Atractylodis Rhizoma]*	*Cyperus rotundus L. [*Cyperaceae*, Cyperi Rhizoma] and Poria cocos (Schw.) Wolf [*Polyporaceae*, Poria]*	41.67	80.00	1.40
7	*Poria cocos* (schw.) Wolf [Polyporaceae, *Poria*]	*Cyperus rotundus L. [*Cyperaceae*, Cyperi Rhizoma] and Citrus reticulata Blanco [*Rutaceae*, Citri Reticulatae Pericarpium]*	41.67	90.00	1.30
8	*Citrus reticulata* Blanco [Rutaceae, *Citri Reticulatae Pericarpium*]	*Cyperus rotundus L. [*Cyperaceae*, Cyperi Rhizoma] and Poria cocos (Schw.) Wolf [*Polyporaceae*, Poria]*	41.67	90.00	1.44
9	*Citrus reticulata Blanco [*Rutaceae*, Citri Reticulatae Pericarpium]*	*Atractylodes lancea (Thunb.) DC. [*Asteraceae*, Atractylodis Rhizoma] and Cyperus rotundus L. [*Cyperaceae*, Cyperi Rhizoma]*	37.50	96.30	1.54
10	*Poria cocos* (schw.) Wolf [Polyporaceae, *Poria*]	*Atractylodes lancea (Thunb.) DC. [*Asteraceae*, Atractylodis Rhizoma] and Cyperus rotundus L. [*Cyperaceae*, Cyperi Rhizoma]*	37.50	88.89	1.28

**FIGURE 9:**
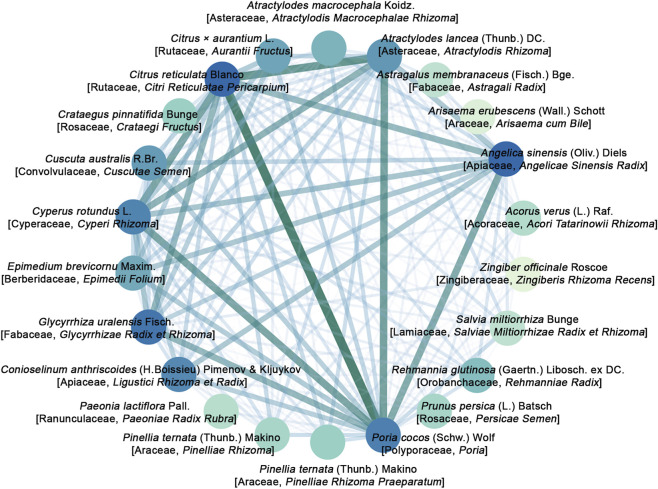
Network visualization of botanical drug co-occurrence patterns.

A network diagram was constructed to visualize the pairwise co-occurrence relationships among botanical drugs (Figure 9), where the thickness of connecting lines represents the association strength. The network analysis identified five botanical drugs as the most central and densely interconnected nodes: *P. cocos* (Schw.) Wolf [Polyporaceae, *Poria*], *C. reticulata* Blanco [Rutaceae, *Citri Reticulatae Pericarpium*], *A. lancea* (Thunb.) DC. [Asteraceae, *Atractylodis Rhizoma*], *C. rotundus* L. [Cyperaceae, *Cyperi Rhizoma*], and *A. sinensis* (Oliv.) Diels [Apiaceae, *Angelicae Sinensis Radix*].

## 4 Discussion

### 4.1 Summary of main findings

This systematic review and meta-analysis evaluated the adjunctive use of THM with conventional therapy for obesity-related PCOS. The pooled results indicate that the combined regimen was significantly superior to conventional therapy alone in improving key clinical, reproductive, and metabolic outcomes, including clinical efficacy rate, clinical pregnancy rate, HOMA-IR, BMI, total testosterone, and the LH/FSH ratio. However, the certainty of this evidence, as assessed by the GRADE framework, was predominantly low to very low. This was largely attributable to methodological shortcomings in the included studies, such as inadequate reporting of randomization and blinding procedures, alongside considerable heterogeneity among studies, which may reflect the distinct metabolic and reproductive subtypes of the PCOS population (Gao et al., 2025).

A central challenge in interpreting these meta-analytic results lies in the substantial heterogeneity, which primarily stems from the diverse and non-standardized compositions of the THM formulas. We therefore employed data mining to systematically explore the formulation data, aiming to identify recurrent patterns that characterize the heterogeneous interventions. Frequency analysis identified the most commonly used botanical drugs. Association rule mining, which prioritizes the strength and reliability of pairwise relationships, revealed that the most robust statistical associations centered on a specific combination of *P. cocos* (Schw.) Wolf [Polyporaceae, *Poria*], *C. reticulata* Blanco [Rutaceae, *Citri Reticulatae Pericarpium*], *A. lancea* (Thunb.) DC. [Asteraceae, *Atractylodis Rhizoma*], and *C. rotundus* L. [Cyperaceae, *Cyperi Rhizoma*]. Network visualization of botanical drug co-occurrence further highlighted these four substances, along with *A. sinensis* (Oliv.) Diels [Apiaceae, *Angelicae Sinensis Radix*], as the most central and interconnected nodes. A notable observation emerged from comparing these results: while *A. sinensis* (Oliv.) Diels [Apiaceae, *Angelicae Sinensis Radix*] was a high-frequency botanical drug and a central network node, it was not part of the strongest pairwise associations defined by the rule mining. This pattern suggests that *A. sinensis* (Oliv.) Diels [Apiaceae, *Angelicae Sinensis Radix*] may have a broader, more generalized pattern of co-use across various formula contexts in the studied dataset, whereas the core four-botanical drug combination exhibits a more distinct and strongly correlated co-occurrence profile. This empirically derived combination offers a data-driven candidate for future research aimed at developing targeted phytotherapeutic strategies.

### 4.2 Limitations

The interpretation of our findings is subject to several important limitations. First, the methodological quality of the included studies substantially constrains the reliability of the evidence. Most studies failed to report allocation concealment or blinding, and none were prospectively registered. These shortcomings introduce a high risk of performance and detection bias, as well as concerns regarding selective outcome reporting, which may collectively inflate the observed effect sizes. Second, the high statistical heterogeneity underscores a fundamental issue in the field: the lack of intervention standardization. Although our data mining identified a recurrent core combination, it does not validate its efficacy nor does it statistically control for the heterogeneity in the meta-analysis. The pooled estimates thus represent an average effect across a wide spectrum of different herbal interventions, limiting their precision and direct clinical interpretability. Third, the exclusive reliance on studies from Chinese databases may affect the generalizability of our findings to other populations and settings. Finally, while association rule mining effectively revealed frequently co-occurring species, it identifies correlation rather than causation. Therefore, the presumed efficacy and any synergistic effects of the identified core combination remain hypothetical and require validation through rigorously controlled experimental and clinical studies.

### 4.3 Implications for practice and future research

The findings of this review, tempered by its significant methodological limitations, inform both cautious clinical consideration and a clear direction for future research. Given the predominantly low-certainty evidence, no definitive recommendations for practice can be made. However, for patients and clinicians interested in an evidence-informed integrative approach, the use of quality-controlled THM formulas, particularly those incorporating the core botanical drug combination identified here, may be considered an option for adjunctive use under professional supervision.

Future clinical research must be designed to address the flaws that limited the primary studies in this meta-analysis. This necessitates conducting large-scale, multi-center, randomized controlled trials that are prospectively registered and adhere strictly to CONSORT reporting guidelines. These trials should employ rigorous methodology including allocation concealment and double-blinding. Where ethically feasible, the use of placebo controls is essential to isolate the specific effects of the botanical intervention. Crucially, to enhance the interpretability and reproducibility of future evidence, such trials could utilize standardized, chemically characterized preparations based on the core botanical drug combination identified here.

### 4.4 Implications of mechanism research

If the preliminary meta-analytic signal that THM may improve outcomes is validated in future rigorous trials, the core botanical drug combination of *P. cocos* (Schw.) Wolf [Polyporaceae, *Poria*], *C. reticulata* Blanco [Rutaceae, *Citri Reticulatae Pericarpium*], *A. lancea* (Thunb.) DC. [Asteraceae, *Atractylodis Rhizoma*], and *C. rotundus* L. [Cyperaceae, *Cyperi Rhizoma*] emerges as a prime candidate for investigating multi-targeted botanical drug therapy. This prompts a shift from traditional descriptive frameworks toward a testable hypothesis grounded in contemporary systems pathophysiology of PCOS.

We propose that the therapeutic potential of this combination likely resides in its capacity to concurrently modulate several interconnected pathological axes that sustain PCOS. Modern conceptualizations highlight a self-perpetuating cycle involving insulin resistance, chronic low-grade inflammation, hyperandrogenism, and dysregulated tissue microenvironments (Ren et al., 2019; Mizgier et al., 2024; Zhang et al., 2025). In this cycle, hyperinsulinemia exacerbates androgen excess, which in turn promotes visceral adiposity and immune activation, fueling a state of chronic inflammation (Sanchez-Garrido and Tena-Sempere, 2020; Rudnicka et al., 2021). The resulting inflammatory milieu then impairs insulin signaling, thereby reinforcing insulin resistance (Lonardo et al., 2024). This entire process is embedded within and amplifies dysfunction in key tissue microenvironments, such as adipose tissue, the ovary, and the endometrium, contributing to the characteristic metabolic and reproductive dysfunction of PCOS (Siddiqui et al., 2022; Xiang et al., 2023).

This pathological framework provides a basis for hypothesizing the combination’s mechanism of action. The constituent botanical drugs possess distinct yet complementary pharmacological profiles that may target these interconnected pathways. For instance, metabolites from *C. reticulata* Blanco [Rutaceae, *Citri Reticulatae Pericarpium*] and *A. lancea* (Thunb.) DC. [Asteraceae, *Atractylodis Rhizoma*] have been linked to improved insulin sensitivity and glucose metabolism in preclinical studies (Castro et al., 2020; Wang et al., 2025). *Poria cocos* (Schw.) Wolf [Polyporaceae, *Poria*] is recognized for its anti-inflammatory and gut microbiota-modulating properties, which may address systemic inflammation (Zhu et al., 2022; Liu et al., 2025). *Cyperus rotundus* L. [Cyperaceae, *Cyperi Rhizoma*] contains metabolites with reported potential influences on steroidogenic activity and hormonal balance (Pirzada et al., 2015; Huang et al., 2025). Crucially, we hypothesize that the clinical benefit arises not merely from the sum of these isolated actions, but from their synergistic interaction. The combination might disrupt the core pathologic network more effectively than any single botanical drug by simultaneously attenuating inflammation, enhancing metabolic function, and fine-tuning endocrine signaling, thereby breaking the self-perpetuating cycle of PCOS.

Validating this integrative hypothesis requires a stepwise translational approach. Initial work should employ computational methods like network pharmacology to predict the combination’s multi-target interactions within established PCOS molecular networks (Wu et al., 2023). Consistent with best practice guidelines in ethnopharmacology, these *in silico* predictions must be followed by experimental confirmation. Subsequent empirical validation should utilize suitable preclinical models to compare the effects of the full combination against its individual constituents. Key readouts must extend beyond single biomarkers to encompass interconnected pathways relevant to immunometabolic crosstalk, ovarian follicle development, and hormonal regulation. This approach will help elucidate the synergistic mechanisms and key bioactive metabolites underlying the observed clinical effects.

## 5 Conclusion

This systematic review indicates that adjunctive therapy with THM may improve key clinical, metabolic, and reproductive outcomes in women with obesity-related PCOS, compared to conventional therapy alone. Data mining of prescription patterns identified a core combination of *P. cocos* (Schw.) Wolf [Polyporaceae, *Poria*], *C. reticulata* Blanco [Rutaceae, *Citri Reticulatae Pericarpium*], *A. lancea* (Thunb.) DC. [Asteraceae, *Atractylodis Rhizoma*], and *C. rotundus* L. [Cyperaceae, *Cyperi Rhizoma*] as a candidate for further phytotherapeutic research. The overall evidence is, however, limited by the methodological shortcomings and substantial heterogeneity observed among the included studies. To definitively establish efficacy and safety, future research should prioritize rigorously designed, large-scale clinical trials utilizing standardized preparations based on this core combination. In parallel, experimental studies are warranted to elucidate the underlying synergistic mechanisms of action.

## Data Availability

The raw data supporting the conclusions of this article will be made available by the authors, without undue reservation.
