# Supplementary material for: Traditional herbal medicine for obesity-related polycystic ovary syndrome: a meta-analysis and data mining study
Source: Front Pharmacol. 2026 Jan 20;16:1738172. doi: 10.3389/fphar.2025.1738172 (PMC12864513; doi:10.3389/fphar.2025.1738172)
Supplement: Supplementary file 1 [file DataSheet1.zip › Data Sheet/Supplementary_Material.docx]

**Supplementary Material**

Contents

[**Supplementary Figure S1.** Subgroup Analysis for HOMA-IR by Diagnostic Criteria 1](#_Toc217639341)

[**Supplementary Figure S2.** Subgroup Analysis for HOMA-IR by Formulation Type 2](#_Toc217639342)

[**Supplementary Figure S3.** Subgroup Analysis for BMI by Diagnostic Criteria 3](#_Toc217639343)

[**Supplementary Figure S4.** Subgroup analysis for BMI by Treatment Duration 4](#_Toc217639344)

[**Supplementary Figure S5.** Subgroup analysis for BMI by Formulation Type 5](#_Toc217639345)

[**Supplementary Figure S6.** Subgroup Analysis for TT by Diagnostic Criteria 6](#_Toc217639346)

[**Supplementary Figure S7.** Subgroup analysis for TT by Treatment Duration 7](#_Toc217639347)

[**Supplementary Figure S8.** Subgroup analysis for TT by Formulation Type 8](#_Toc217639348)

[**Supplementary Figure S9.** Subgroup Analysis for LH/FSH Ratio by Diagnostic Criteria 9](#_Toc217639349)

[**Supplementary Figure S10.** Subgroup Analysis for LH/FSH Ratio by Formulation Type 10](#_Toc217639350)

[**Supplementary Figure S11.** Results of Sensitivity Analysis 11](#_Toc217639351)

[**Supplementary Figure S12.** Funnel Plots of Primary Outcomes 12](#_Toc217639352)

[**Supplementary Figure S13.** Egger's Test for Primary Outcomes 13](#_Toc217639353)

[**Supplementary Figure S14.** Trim-and-Fill Analysis for Primary Outcomes 14](#_Toc217639354)

[**Supplementary Table S1.** Search Strategy 15](#_Toc217639355)

[**Supplementary Table S2.** Methodological quality of the included studies 16](#_Toc217639356)

[**Supplementary Table S3.** GRADE evidence profile 18](#_Toc217639357)

[**Supplementary Table S4.** Herbal Formulations Used in the Included Studies 19](#_Toc217639358)

Supplementary Figure S1. Subgroup Analysis for HOMA-IR by Diagnostic Criteria


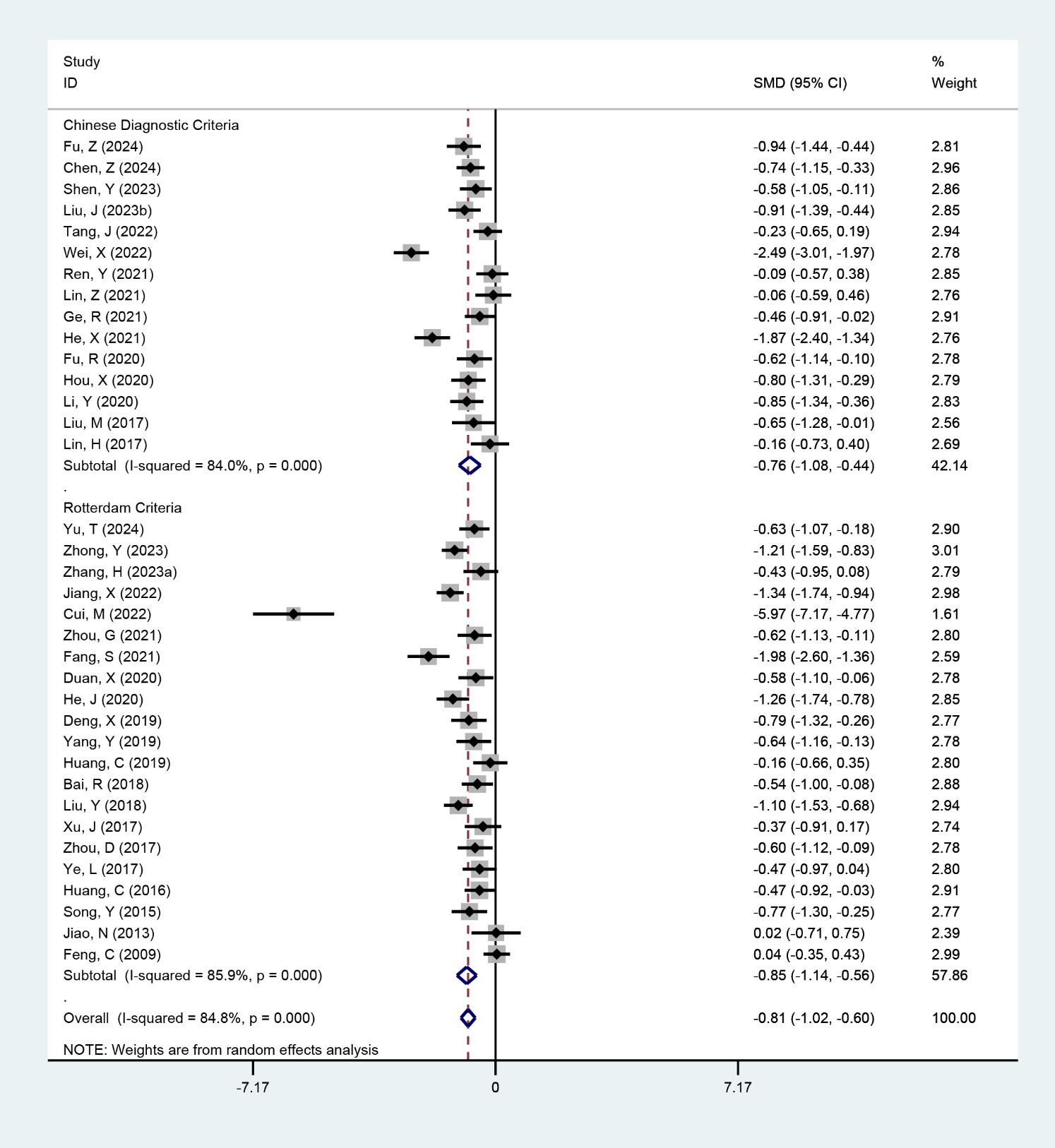


Supplementary Figure S2. Subgroup Analysis for HOMA-IR by Formulation Type


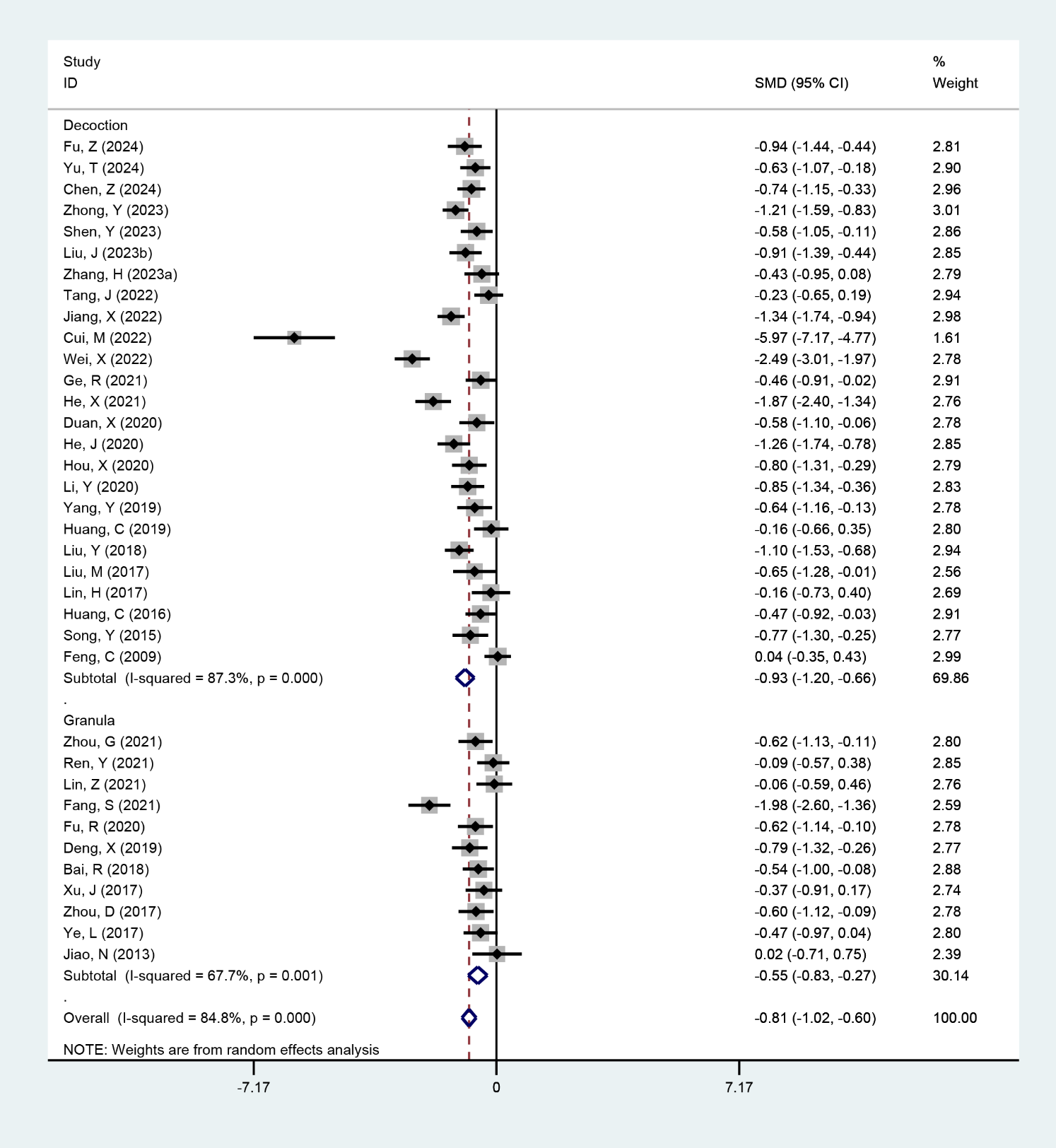


Supplementary Figure S3. Subgroup Analysis for BMI by Diagnostic Criteria


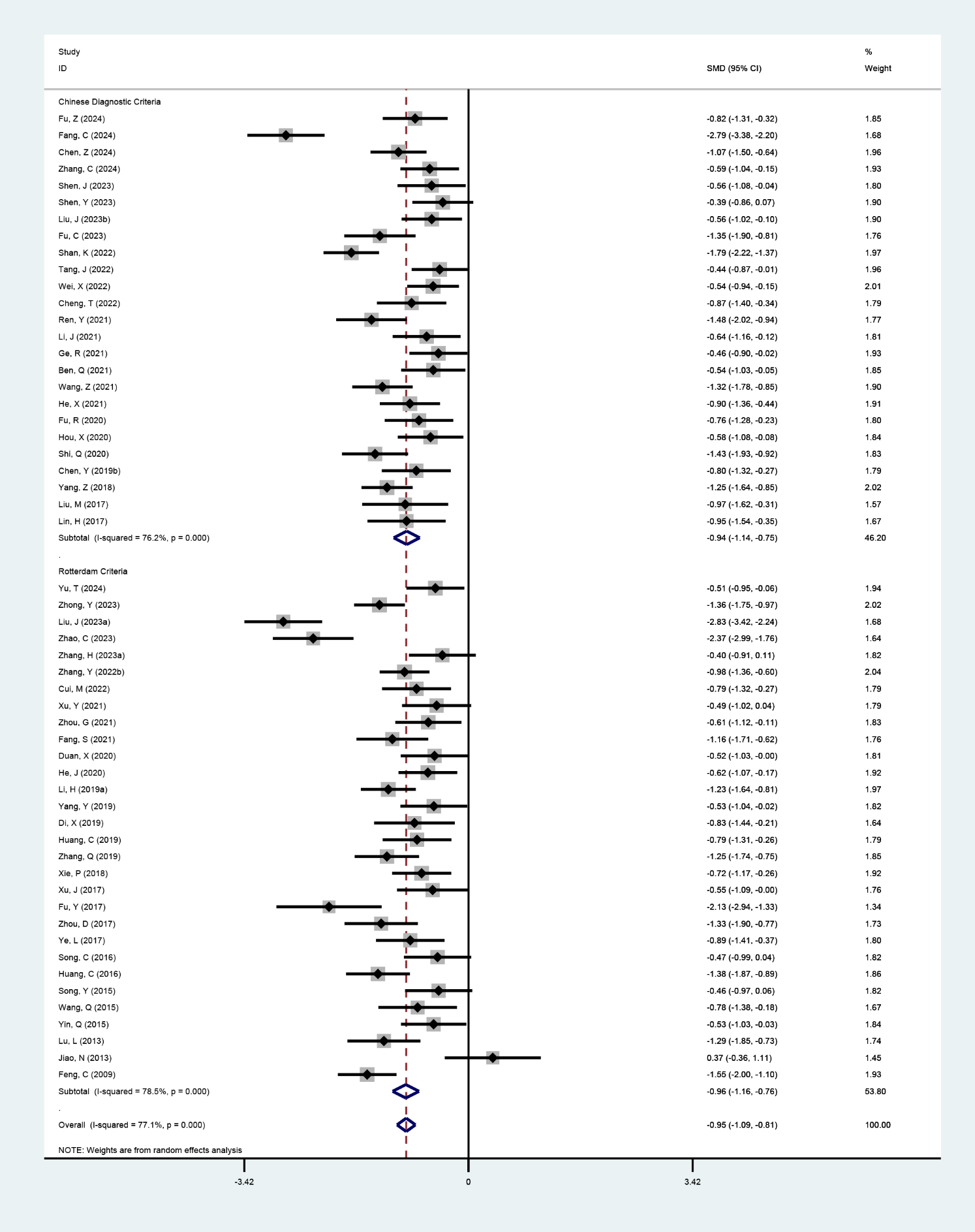


Supplementary Figure S4. Subgroup analysis for BMI by Treatment Duration


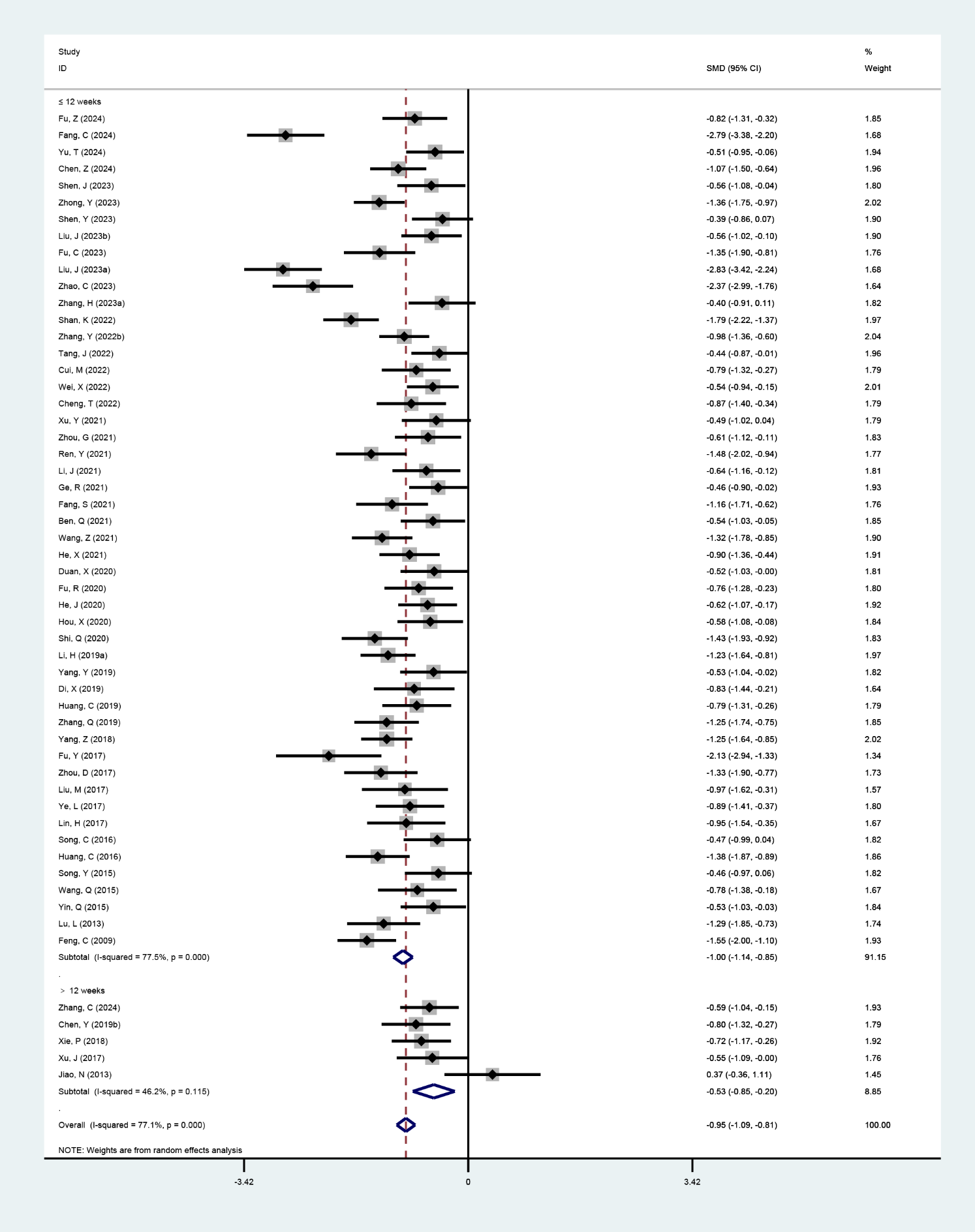


Supplementary Figure S5. Subgroup analysis for BMI by Formulation Type


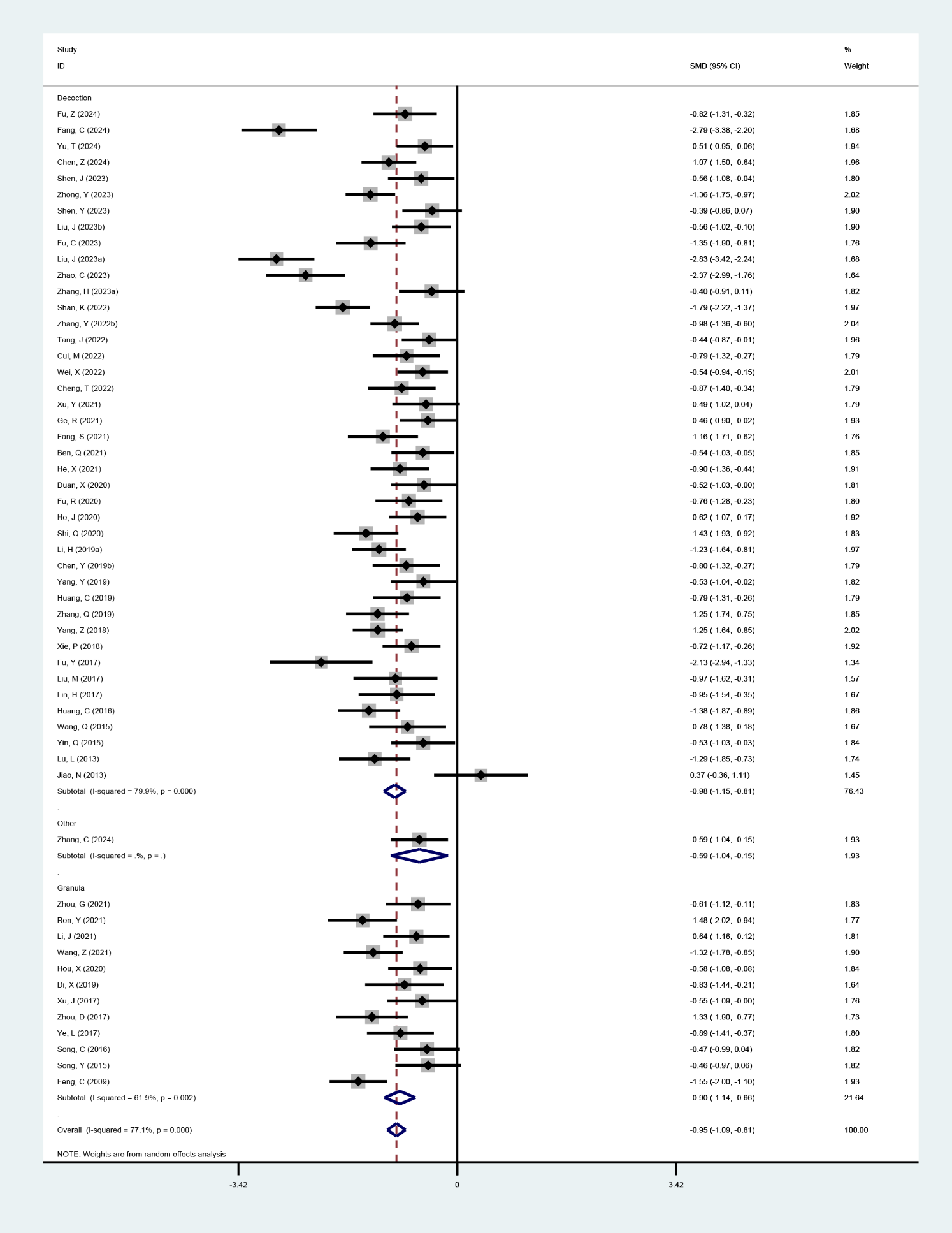


Supplementary Figure S6. Subgroup Analysis for TT by Diagnostic Criteria


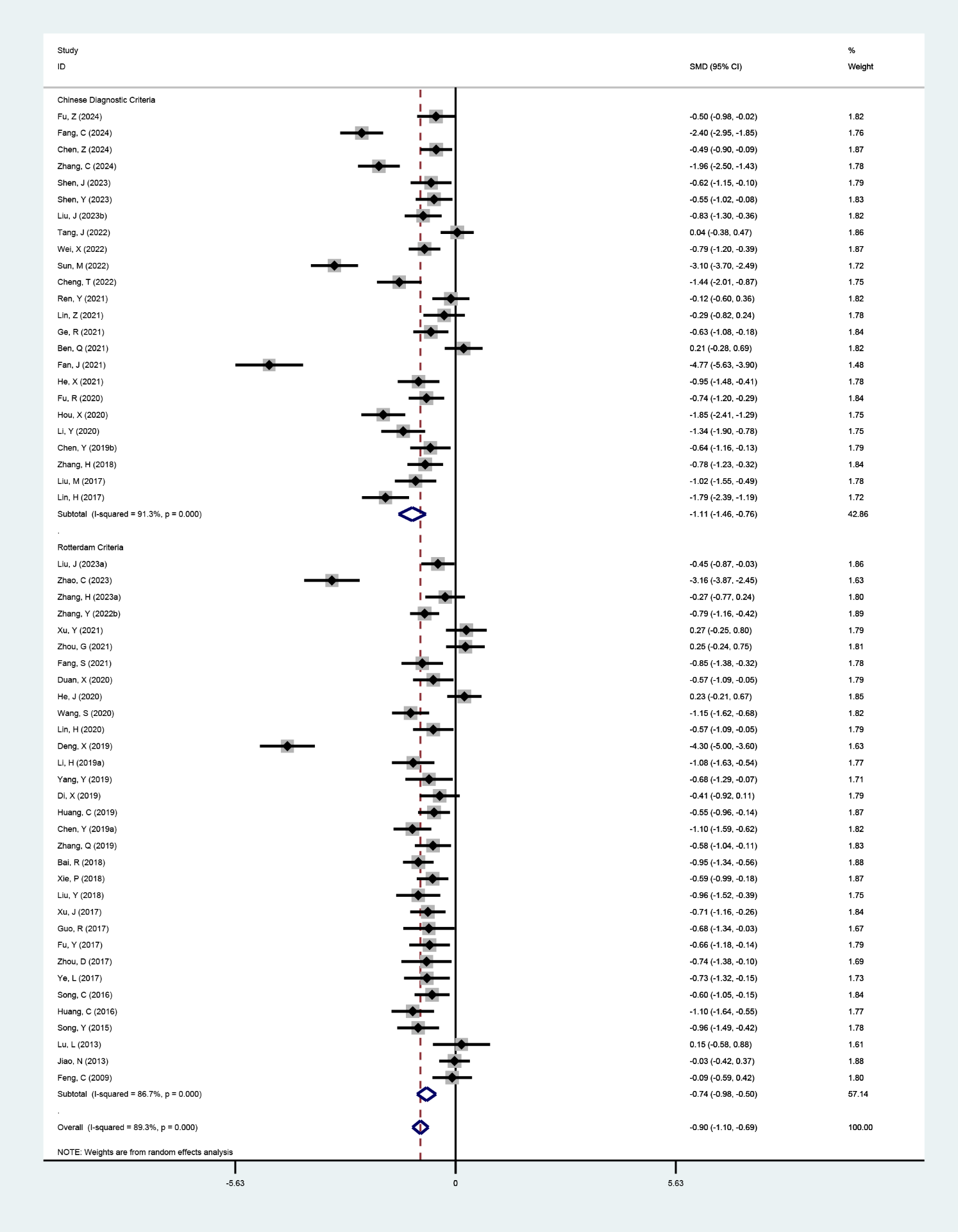


Supplementary Figure S7. Subgroup analysis for TT by Treatment Duration


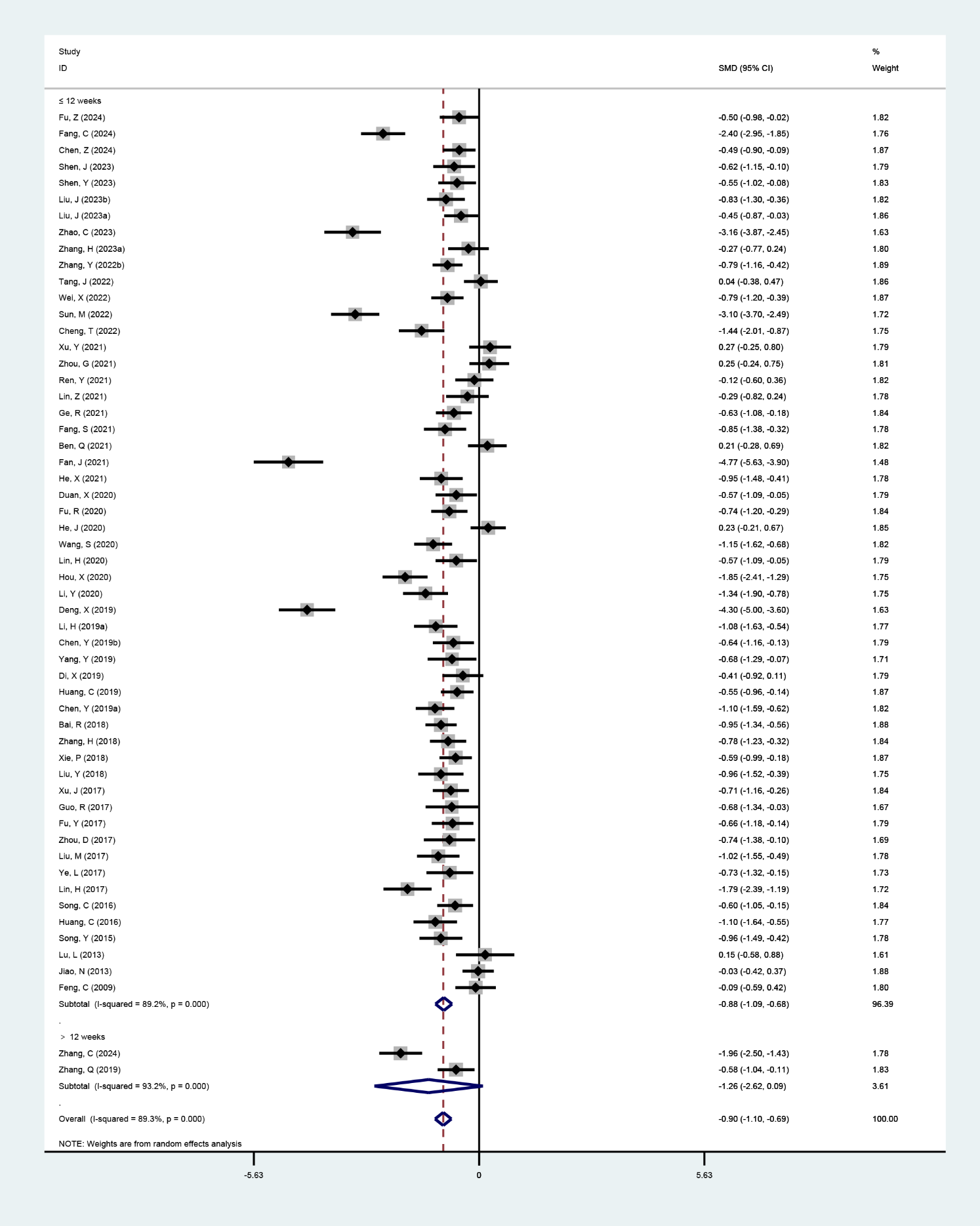


Supplementary Figure S8. Subgroup analysis for TT by Formulation Type


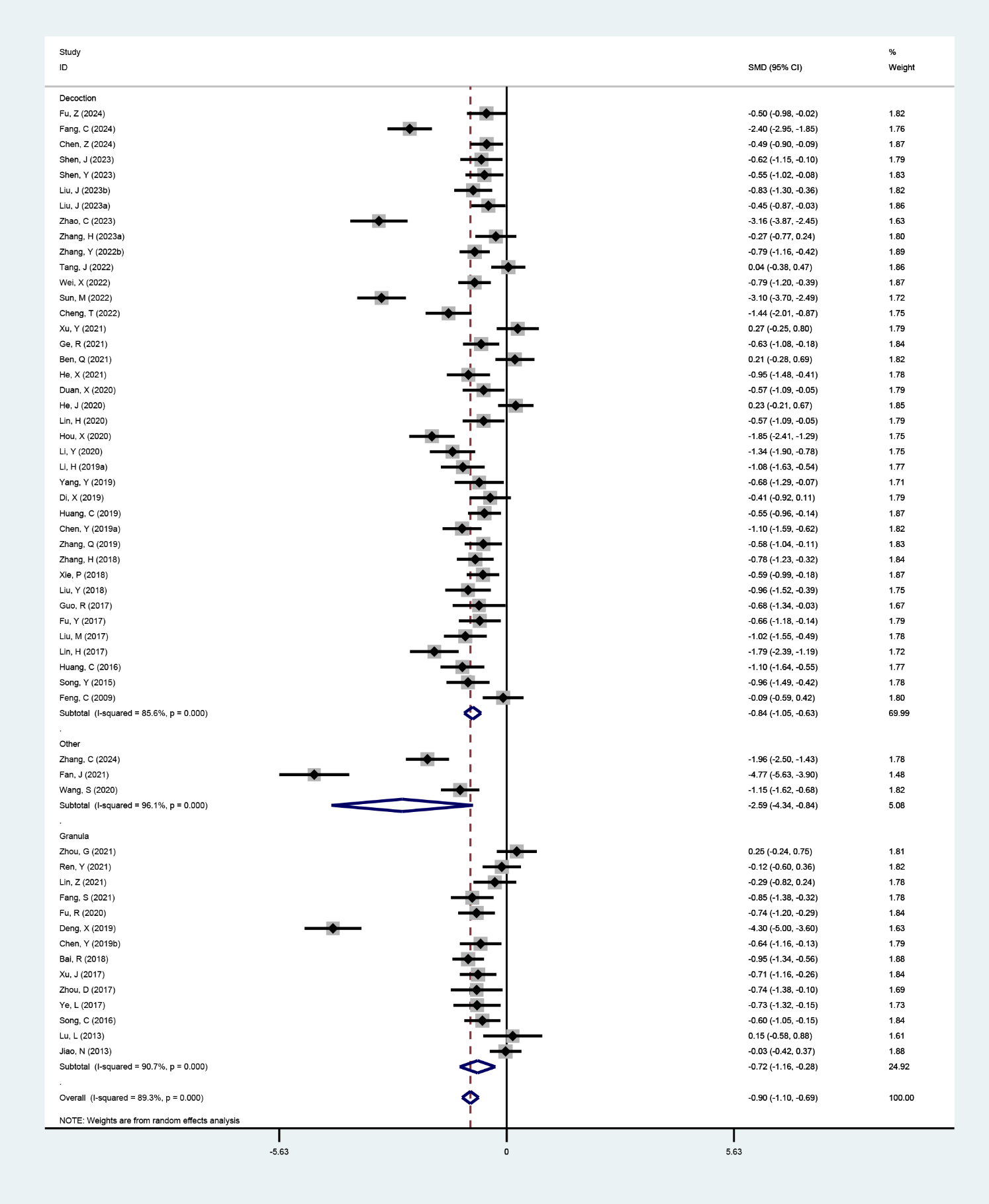


Supplementary Figure S9. Subgroup Analysis for LH/FSH Ratio by Diagnostic Criteria


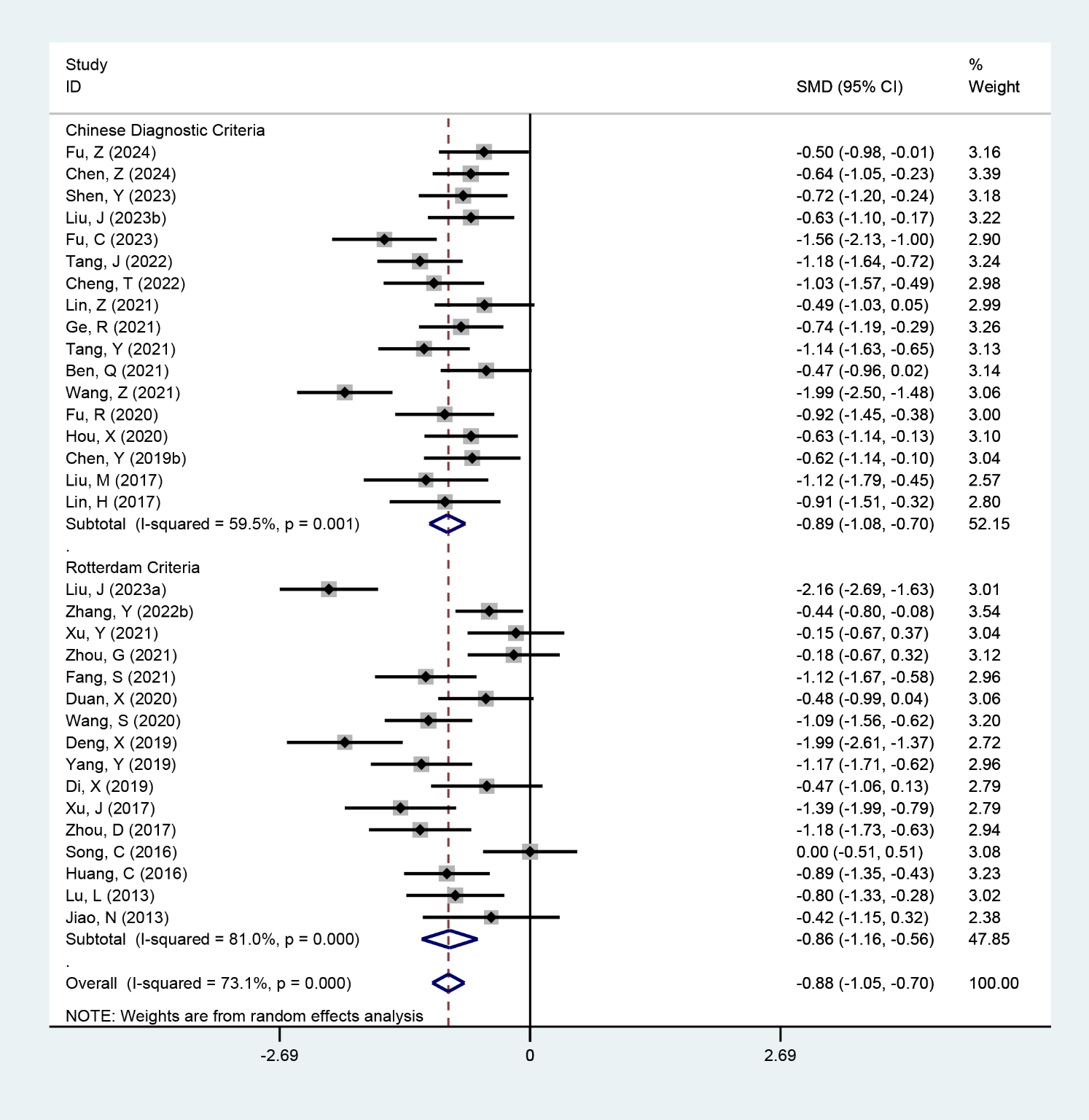


Supplementary Figure S10. Subgroup Analysis for LH/FSH Ratio by Formulation Type

**
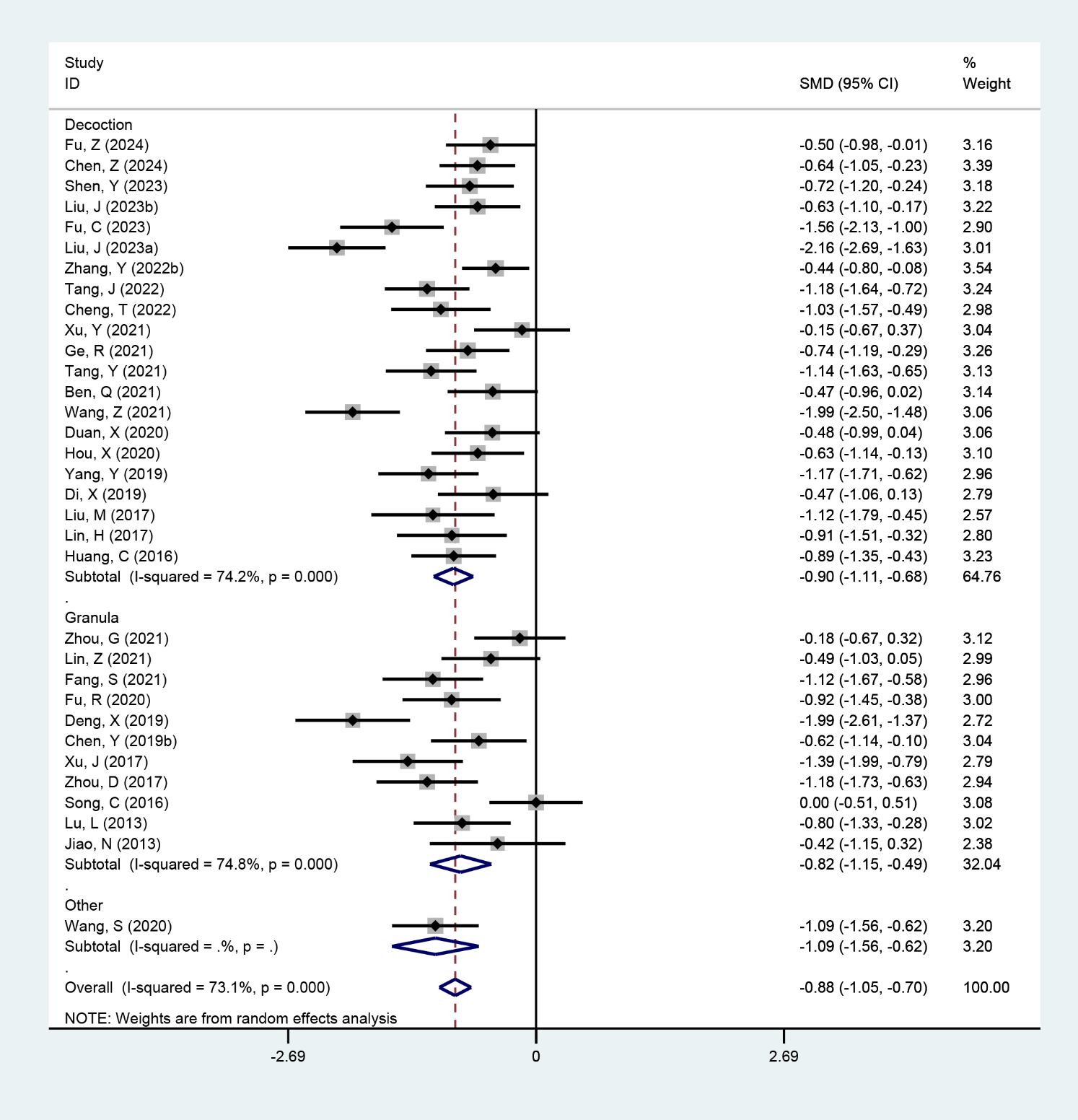
**

Supplementary Figure S11. Results of Sensitivity Analysis


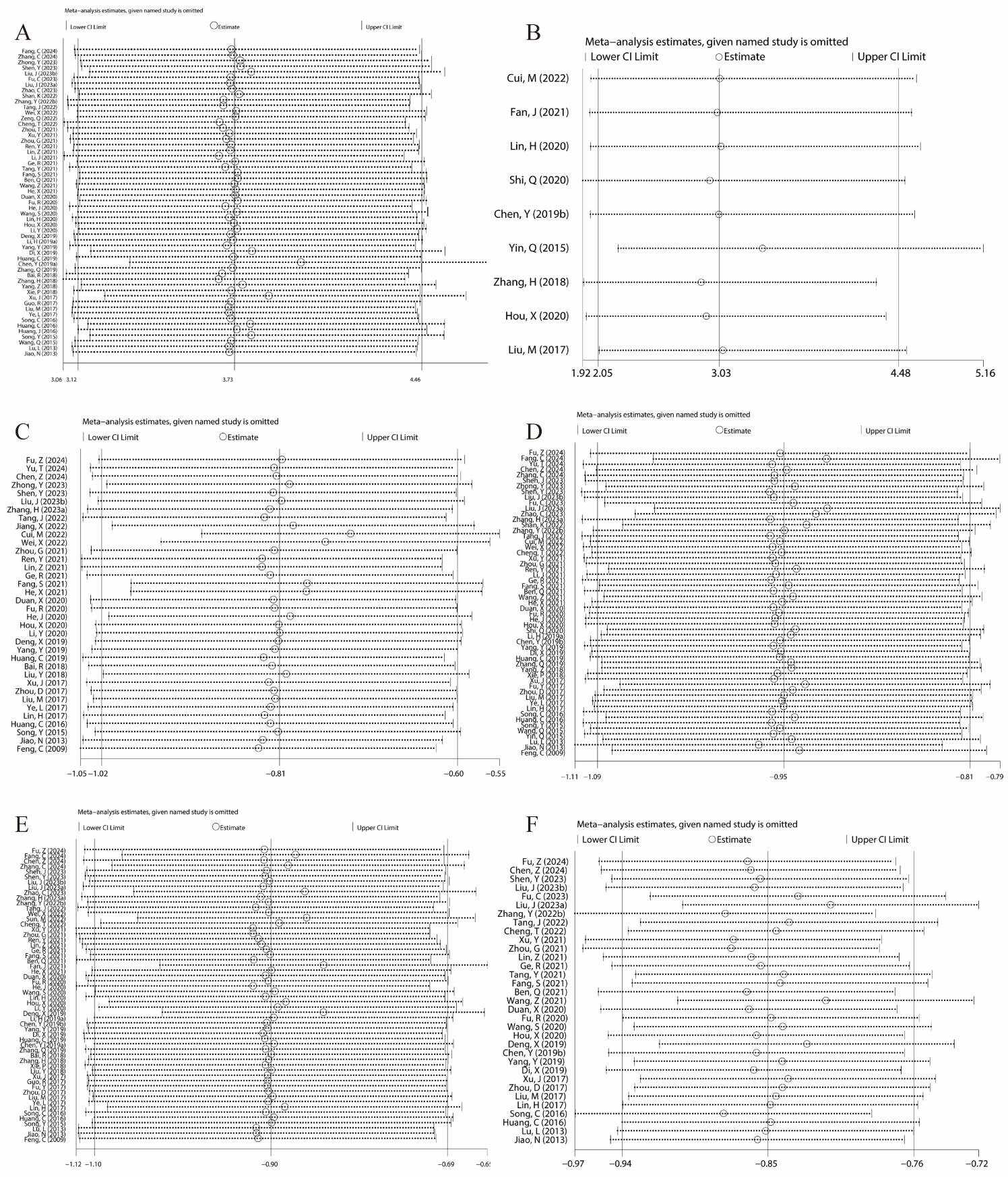


Note**:** (A) Clinical efficiency rate; (B) Clinical pregnancy rate; (C) HOMA-IR; (D) BMI; (E) TT; (F) LH/FSH ratio.

Supplementary Figure S12. Funnel Plots of Primary Outcomes


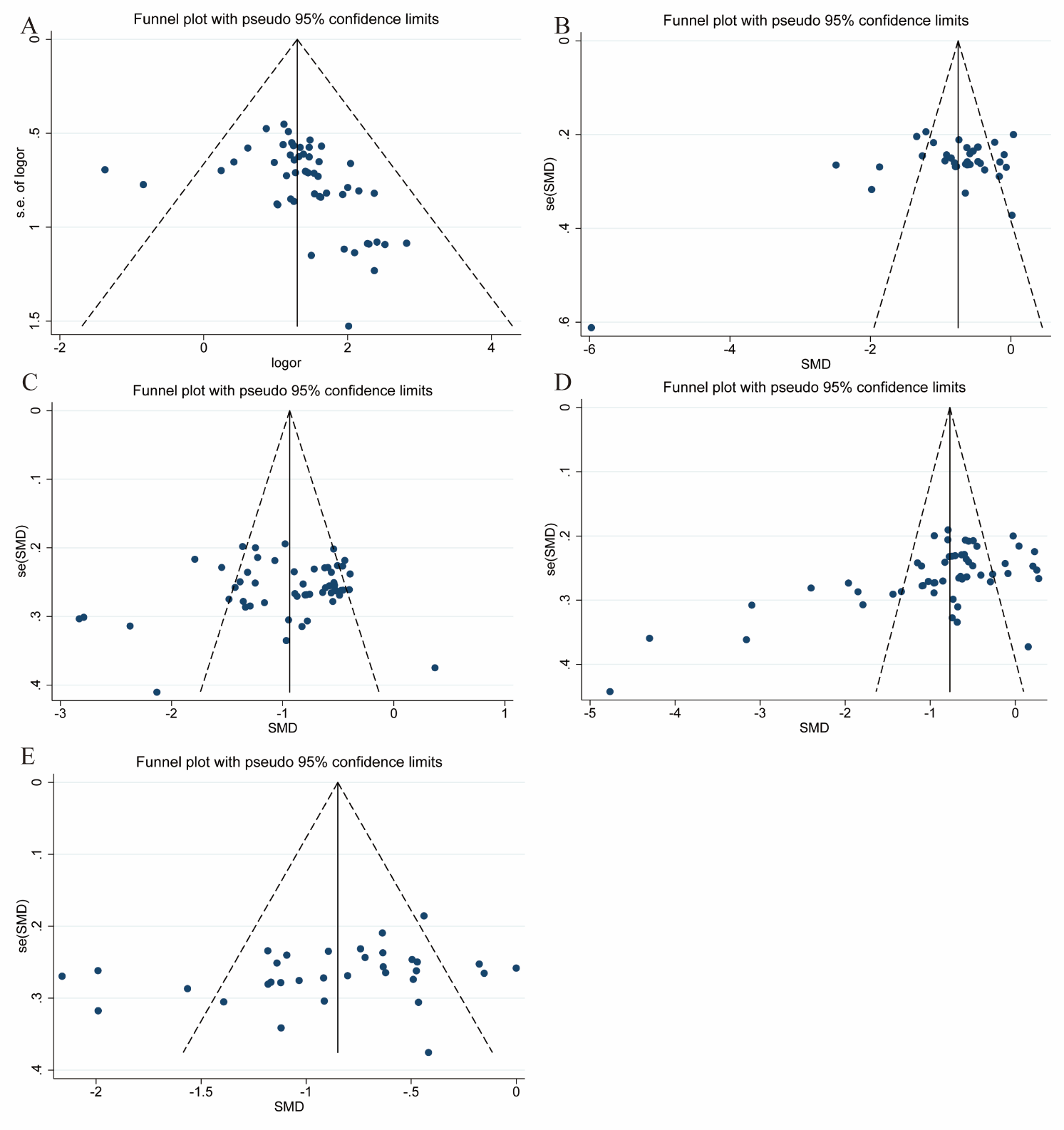


Note: (A) Clinical efficiency rate; (B) HOMA-IR; (C) BMI; (D) TT; (E) LH/FSH ratio.

Supplementary Figure S13. Egger's Test for Primary Outcomes


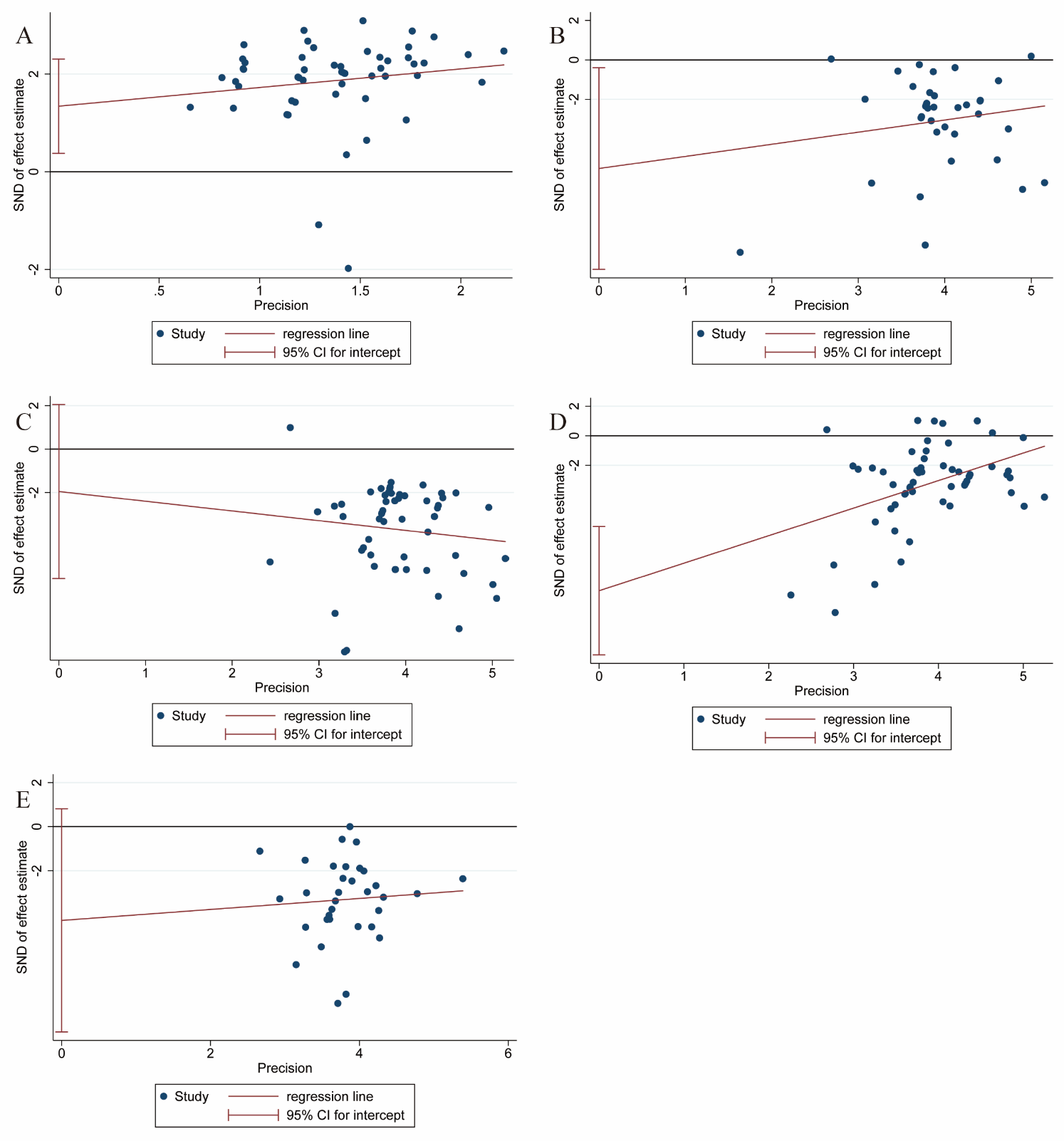


Note: (A) Clinical efficiency rate; (B) HOMA-IR; (C) BMI; (D) TT; (E) LH/FSH ratio.

Supplementary Figure S14. Trim-and-Fill Analysis for Primary Outcomes


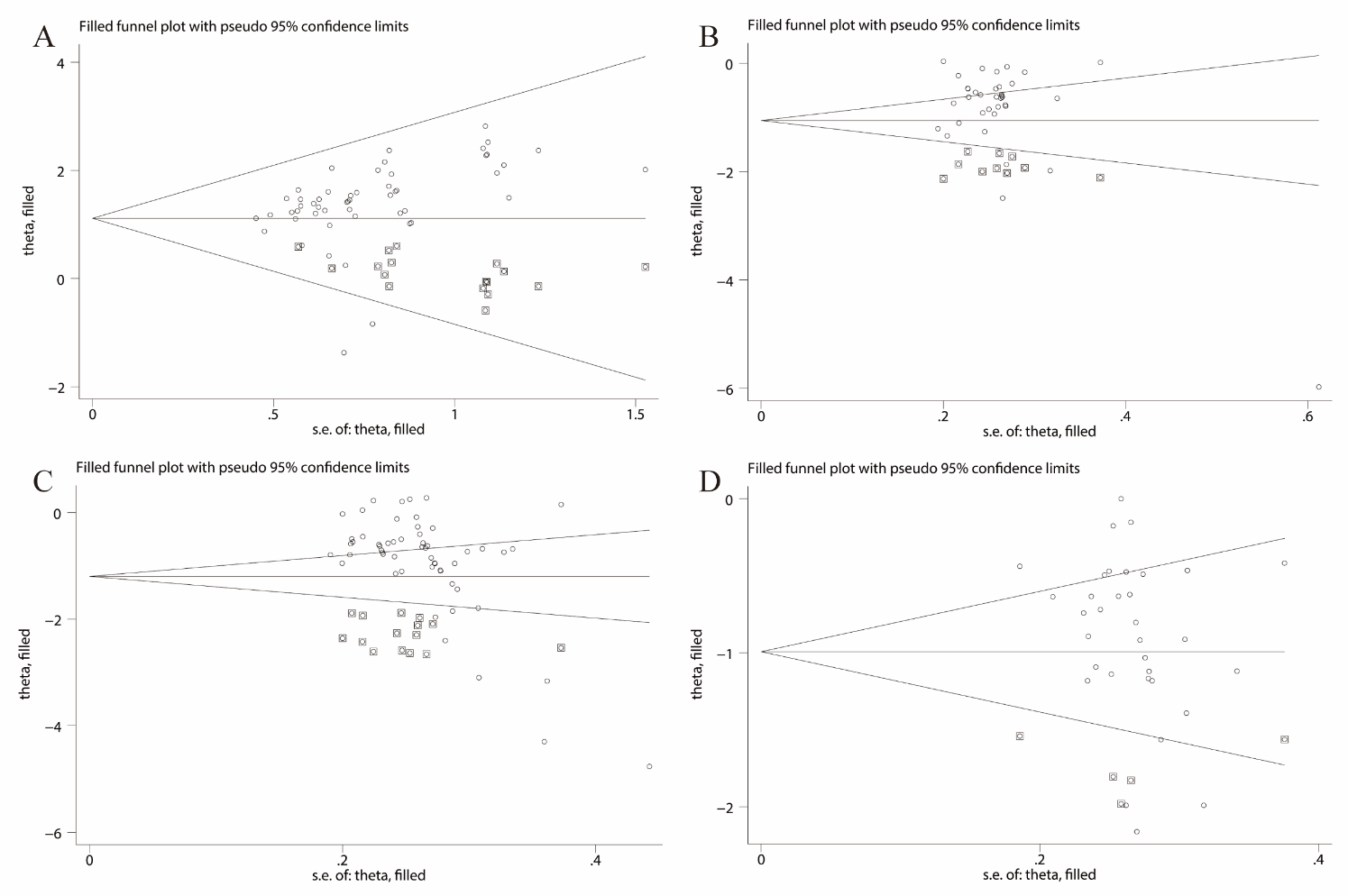


Note: (A) Clinical efficiency rate; (B) HOMA-IR; (C) TT; (D) LH/FSH ratio.

Supplementary Table S1. Search Strategy

| **Database** | **Search Strategies** |
| --- | --- |
| Pubmed | ("traditional chinese medicine"[Title/Abstract] OR "chinese herb"[Title/Abstract] OR "traditional chinese medicine compound"[Title/Abstract] OR "chinese patent medicine"[Title/Abstract] OR "Decoction"[Title/Abstract] OR "capsule"[Title/Abstract] OR "powder"[Title/Abstract] OR "pill"[Title/Abstract] OR "granula"[Title/Abstract]) AND ("polycystic ovary syndrome"[Title/Abstract] OR "PCOS"[Title/Abstract]) AND ("obesity"[Title/Abstract] OR "weight loss"[Title/Abstract]) |
| Web of science | Query #1=((((((((TS=(traditional chinese medicine)) OR TS=(chinese herb)) OR TS=(traditional chinese medicine compound)) OR TS=(chinese patent medicine)) OR TS=(Decoction)) OR TS=(capsule)) OR TS=(powder)) OR TS=(pill)) OR TS=(granula)  Query #2=(TS=(polycystic ovary syndrome)) OR TS=(PCOS)  Query #3=(TS=（obesity）) OR TS=(weight loss)  Query #4=#1 AND #2 AND #3 |
| Sinomed | ( "中医药"[常用字段:智能] OR "中草药"[常用字段:智能] OR "中药"[常用字段:智能] OR "中药复方"[常用字段:智能] OR "中成药"[常用字段:智能] OR "方"[常用字段:智能] OR "汤"[常用字段:智能] OR "散"[常用字段:智能] OR "颗粒"[常用字段:智能] OR "膏"[常用字段:智能]) AND "多囊卵巢综合征"[常用字段:智能] AND( "肥胖"[常用字段:智能] OR "减重"[常用字段:智能]) AND( "临床研究"[常用字段:智能] OR "临床观察"[常用字段:智能]) |
| CNKI | #1 SU=中医药 + 中草药 + 中药 + 中药复方 + 方 + 汤 + 散 + 颗粒 + 膏  #2 SU=多囊卵巢综合征  #3 SU=肥胖 + 减重  #4 SU=临床研究 + 临床观察  #5 #1 AND #2 AND #3 AND #4 |
| Wanfang | 主题:(中医药 OR 中草药 OR 中药 OR 中药复方 OR 中成药 OR 方 OR 汤 OR 散 OR 颗粒 OR 胶囊 OR 膏) and 主题:(肥胖) and 主题:(多囊卵巢综合征) and 主题:(临床研究 OR 临床观察) |
| VIP | (((((((((((((题名或关键词=中医药 OR 题名或关键词=中草药) OR 题名或关键词=中药) OR 题名或关键词=中药复方) OR 题名或关键词=中成药) OR 题名或关键词=方) OR 题名或关键词=汤) OR 题名或关键词=散) OR 题名或关键词=颗粒) OR 题名或关键词=胶囊) OR 题名或关键词=膏) AND 题名或关键词=肥胖) AND 题名或关键词=多囊卵巢综合征) AND (题名或关键词=临床研究 OR 题名或关键词=临床观察)) |

Supplementary Table S2. Methodological quality of the included studies

| Study ID | D1 | D2 | D3 | D4 | D5 | Overall |
| --- | --- | --- | --- | --- | --- | --- |
| Fu, Z (2024) | Low | Low | Low | Low | Some concerns | Some concerns |
| Fang, C (2024) | Low | Low | Low | Low | Some concerns | Some concerns |
| Yu, T (2024) | Low | Low | Low | Low | Some concerns | Some concerns |
| Chen, Z (2024) | Low | Low | Low | Low | Some concerns | Some concerns |
| Zhang, C (2024) | High | Low | Low | Low | Some concerns | High |
| Shen, J (2023) | Low | Low | Low | Low | Some concerns | Some concerns |
| Zhong, Y (2023) | Low | Low | Low | Low | Some concerns | Some concerns |
| Shen, Y (2023) | Low | Low | Low | Low | Some concerns | Some concerns |
| Liu, J (2023b) | Some concerns | Low | Low | Low | Some concerns | Some concerns |
| Fu, C (2023) | Some concerns | Low | Low | Low | Some concerns | Some concerns |
| Liu, J (2023a) | Low | Low | Low | Low | Some concerns | Some concerns |
| Zhao, C (2023) | Low | Low | Low | Low | Some concerns | Some concerns |
| Zhang, H (2023a) | Low | Low | Low | Low | Some concerns | Some concerns |
| Shan, K (2022) | Low | Low | Low | Low | Some concerns | Some concerns |
| Zhang, Y (2022b) | Low | Low | Low | Low | Some concerns | Some concerns |
| Tang, J (2022) | Low | Low | Low | Low | Some concerns | Some concerns |
| Jiang, X (2022) | Some concerns | Low | Low | Low | Some concerns | Some concerns |
| Cui, M (2022) | Some concerns | Low | Low | Low | Some concerns | Some concerns |
| Wei, X (2022) | Low | Low | Low | Low | Some concerns | Some concerns |
| Sun, M (2022) | Low | Low | Low | Low | Some concerns | Some concerns |
| Zeng, Q (2022) | Some concerns | Low | Low | Low | Some concerns | Some concerns |
| Cheng, T (2022) | Some concerns | Low | Low | Low | Some concerns | Some concerns |
| Zhou, T (2021) | Low | Low | Low | Low | Some concerns | Some concerns |
| Xu, Y (2021) | Low | Low | Low | Low | Some concerns | Some concerns |
| Zhou, G (2021) | Low | Low | Low | Low | Some concerns | Some concerns |
| Ren, Y (2021) | Some concerns | Low | Low | Low | Some concerns | Some concerns |
| Lin, Z (2021) | Low | Low | Low | Low | Some concerns | Some concerns |
| Li, J (2021) | Low | Low | Low | Low | Some concerns | Some concerns |
| Ge, R (2021) | Low | Low | Low | Low | Some concerns | Some concerns |
| Tang, Y (2021) | Low | Low | Low | Low | Some concerns | Some concerns |
| Fang, S (2021) | Low | Low | Low | Low | Some concerns | Some concerns |
| Ben, Q (2021) | Some concerns | Low | Low | Low | Some concerns | Some concerns |
| Fan, J (2021) | Some concerns | Low | Low | Low | Some concerns | Some concerns |
| Wang, Z (2021) | High | Low | Low | Low | Some concerns | High |
| He, X (2021) | Low | Low | Low | Low | Some concerns | Some concerns |
| Duan, X (2020) | Some concerns | Low | Low | Low | Some concerns | Some concerns |
| Fu, R (2020) | Low | Low | Low | Low | Some concerns | Some concerns |
| He, J (2020) | Some concerns | Low | Low | Low | Some concerns | Some concerns |
| Wang, S (2020) | Some concerns | Low | Low | Low | Some concerns | Some concerns |
| Lin, H (2020) | Some concerns | Low | Low | Low | Some concerns | Some concerns |
| Hou, X (2020) | Some concerns | Low | Low | Low | Some concerns | Some concerns |
| Shi, Q (2020) | Low | Low | Low | Low | Some concerns | Some concerns |
| Li, Y (2020) | Low | Low | Low | Low | Some concerns | Some concerns |
| Deng, X (2019) | Some concerns | Low | Low | Low | Some concerns | Some concerns |
| Li, H (2019a) | Low | Low | Low | Low | Some concerns | Some concerns |
| Chen, Y (2019b) | Some concerns | Low | Low | Low | Some concerns | Some concerns |
| Yang, Y (2019) | Low | Low | Low | Low | Some concerns | Some concerns |
| Di, X (2019) | High | Low | Low | Low | Some concerns | High |
| Huang, C (2019) | Some concerns | Low | Low | Low | Some concerns | Some concerns |
| Chen, Y (2019a) | Low | Low | Low | Low | Some concerns | Some concerns |
| Zhang, Q (2019) | Some concerns | Low | Low | Low | Some concerns | Some concerns |
| Bai, R (2018) | Low | Low | Low | Low | Some concerns | Some concerns |
| Zhang, H (2018) | Low | Low | Low | Low | Some concerns | Some concerns |
| Yang, Z (2018) | High | Low | Low | Low | Some concerns | High |
| Xie, P (2018) | Low | Low | Low | Low | Some concerns | Some concerns |
| Liu, Y (2018) | Low | Low | Low | Low | Some concerns | Some concerns |
| Xu, J (2017) | Low | Low | Low | Low | Some concerns | Some concerns |
| Guo, R (2017) | Some concerns | Low | Low | Low | Some concerns | Some concerns |
| Fu, Y (2017) | Some concerns | Low | Low | Low | Some concerns | Some concerns |
| Zhou, D (2017) | Low | Low | Low | Low | Some concerns | Some concerns |
| Liu, M (2017) | Low | Low | Low | Low | Some concerns | Some concerns |
| Ye, L (2017) | Low | Low | Low | Low | Some concerns | Some concerns |
| Lin, H (2017) | Low | Low | Low | Low | Some concerns | Some concerns |
| Song, C (2016) | Low | Low | Low | Low | Some concerns | Some concerns |
| Huang, C (2016) | Low | Low | Low | Low | Some concerns | Some concerns |
| Huang, J (2016) | Some concerns | Low | Low | Low | Some concerns | Some concerns |
| Song, Y (2015) | Low | Low | Low | Low | Some concerns | Some concerns |
| Wang, Q (2015) | Some concerns | Low | Low | Low | Some concerns | Some concerns |
| Yin, Q (2015) | Some concerns | Low | Low | Low | Some concerns | Some concerns |
| Lu, L (2013) | Some concerns | Low | Low | Low | Some concerns | Some concerns |
| Jiao, N (2013) | Some concerns | Low | Low | Low | Some concerns | Some concerns |
| Feng, C (2009) | Low | Low | Low | Low | Some concerns | Some concerns |

Note: Domains of bias assessment: D1, Randomization process; D2, Deviations from intended interventions; D3, Missing outcome data; D4, Measurement of the outcome; D5, Selection of the reported result.

Supplementary Table S3. GRADE evidence profile

| **Certainty assessment** | | | | | | | **№ of patients** | | **Effect** | | **Certainty** | **Importance** |
| --- | --- | --- | --- | --- | --- | --- | --- | --- | --- | --- | --- | --- |
| **№ of studies** | **Study design** | **Risk of bias** | **Inconsistency** | **Indirectness** | **Imprecision** | **Other considerations** | **[Chinese herbal formula+conventional pharmacotherapy]** | **[conventional pharmacotherapy]** | **Relative (95% CI)** | **Absolute (95% CI)** |  |  |
| **Clinical Efficacy Rate** | | | | | | | | | | | | |
| 55 | randomised trials | serious^a^ | not serious | not serious | not serious | publication bias strongly suspected^b^ | 1853/2047 (90.5%) | 1469/2027 (72.5%) | **OR 3.73** (3.12 to 4.46) | **183 more per 1,000** (from 167 more to 197 more) | ⨁⨁◯◯ Low^a,b^ | CRITICAL |
| **Clinical Pregnancy Rate** | | | | | | | | | | | | |
| 9 | randomised trials | serious^a^ | not serious | not serious | not serious | none | 118/288 (41.0%) | 58/288 (20.1%) | **OR 3.03** (2.05 to 4.48) | **232 more per 1,000** (from 139 more to 329 more) | ⨁⨁⨁◯ Moderate^a^ | CRITICAL |
| **BMI** | | | | | | | | | | | | |
| 55 | randomised trials | serious^a^ | serious^c^ | not serious | not serious | none | 1984 | 1965 | - | SMD **0.95 SD lower** (1.09 lower to 0.81 lower) | ⨁⨁◯◯ Low^a,c^ | IMPORTANT |
| **TT** | | | | | | | | | | | | |
| 56 | randomised trials | serious^a^ | serious^c^ | not serious | not serious | publication bias strongly suspected^b^ | 2012 | 1988 | - | SMD **0.9 SD lower** (1.1 lower to 0.69 lower) | ⨁◯◯◯ Very low^a,b,c^ | NOT IMPORTANT |
| **LH/FSH ratio** | | | | | | | | | | | | |
| 33 | randomised trials | serious^a^ | serious^c^ | not serious | not serious | none | 1109 | 1089 | - | SMD **0.88 SD lower** (1.05 lower to 0.7 lower) | ⨁⨁◯◯ Low^a,c^ | NOT IMPORTANT |
| **HOMA-IR** | | | | | | | | | | | | |
| 36 | randomised trials | serious^a^ | serious^c^ | not serious | not serious | publication bias strongly suspected^b^ | 1300 | 1278 | - | SMD **0.81 SD lower** (1.02 lower to 0.6 lower) | ⨁◯◯◯ Very low^a,b,c^ | IMPORTANT |

Note: Reasons for downgrading: a, The included trials were at some risk of bias due to inadequacies in randomization or blinding; b, Considerable heterogeneity was observed, and confidence intervals showed poor overlap; C, Publication bias was suspected based on observable funnel plot asymmetry, which was confirmed by a significant Egger's test.

Supplementary Table S4. Herbal Formulations Used in the Included Studies

| Study | Formulation Name | Herbal Composition | Formulation Type |
| --- | --- | --- | --- |
| Fu, Z (2024) | Erxian Qiling Decoction | *Epimedium brevicornu* Maxim. [Berberidaceae, *Epimedii Folium*] 12g, *Curculigo orchioides* Gaertn. [Hypoxidaceae, *Curculiginis Rhizoma*] 9g, *Astragalus membranaceus* (Fisch.) Bge. [Fabaceae, *Astragali Radix*] 30g, *Poria cocos* (Schw.) Wolf [Polyporaceae, *Poriae Cortex*] 30g, *Angelica sinensis* (Oliv.) Diels [Apiaceae, *Angelicae Sinensis Radix*] 12g, *Gynochthodes officinalis* (F.C.How) Razafim. & B.Bremer [Rubiaceae, *Morindae Officinalis Radix*] 12g, *Phellodendron chinense* C.K.Schneid. [Rutaceae, *Phellodendri Chinensis Cortex*] 30g, *Anemarrhena asphodeloides* Bunge [Asparagaceae, *Anemarrhenae Rhizoma*] 30g, *Rehmannia glutinosa* (Gaertn.) Libosch. ex DC. [Orobanchaceae, *Rehmanniae Radix*] 12g, *Ligustrum lucidum* W.T.Aiton [Oleaceae, *Ligustri Lucidi Fructus*] 15g, *Dipsacus asper* Wall. ex DC. [Caprifoliaceae, *Dipsaci Radix*] 15g, *Citrus × aurantium* L. [Rutaceae, *Aurantii Fructus*] 12g, *Cyperus rotundus* L. [Cyperaceae, *Cyperi Rhizoma*] 12g, *Sparganium stoloniferum* (Buch.-Ham. ex Graebn.) Buch.-Ham. ex Juz. [Typhaceae, *Sparganii Rhizoma*] 12g, *Curcuma phaeocaulis* Valeton [Zingiberaceae, *Curcumae Rhizoma*] 12g, *Ephedra sinica* Stapf [Ephedraceae, *Ephedrae Herba*] 12g, *Paeonia lactiflora* Pall. [Ranunculaceae, *Paeoniae Radix Alba*] 30g, *Glycyrrhiza uralensis* Fisch. [Fabaceae, *Glycyrrhizae Radix et Rhizoma*] 12g | Decoction |
| Fang, C (2024) | Modified Cangfu Daotan Decoction | *Atractylodes lancea* (Thunb.) DC. [Asteraceae, *Atractylodis Rhizoma*] 15g, *Cyperus rotundus*L. [Cyperaceae, *Cyperi Rhizoma*] 15g, *Poria cocos* (Schw.) Wolf [Polyporaceae, *Poria*] 10g, *Citrus reticulata* Blanco [Rutaceae, *Citri Reticulatae Pericarpium*] 10g, *Prunus persica* (L.) Batsch [Rosaceae, *Persicae Semen*] 10g, *Pinellia ternata* (Thunb.) Makino [Araceae, *Pinelliae Rhizoma*] 6g, *Citrus × aurantium* L. [Rutaceae, *Aurantii Fructus*] 6g, *Arisaema erubescens* (Wall.) Schott [Araceae, *Arisaematis Rhizoma*] 6g, Massa Medicata Fermentata 6g, *Angelica sinensis* (Oliv.) Diels [Apiaceae, *Angelicae Sinensis Radix*] 6g, *Prunella vulgaris* L. [Lamiaceae, *Prunellae Spica*] 6g, *Conioselinum anthriscoides* (H.Boissieu) Pimenov & Kljuykov [Apiaceae, *Ligustici Rhizoma et Radix*] 6g, *Glycyrrhiza uralensis* Fisch. [Fabaceae, *Glycyrrhizae Radix et Rhizoma*] 6g, *Zingiber officinale* Roscoe [Zingiberaceae, *Zingiberis Rhizoma Recens*] 3g | Decoction |
| Yu, T (2024) | Gexia Zhuyu Decoction | *Prunus persica* (L.) Batsch [Rosaceae, *Persicae Semen*] 9g, *Carthamus tinctorius* L. [Asteraceae, *Carthami Flos*] 9g, *Angelica sinensis* (Oliv.) Diels [Apiaceae, *Angelicae Sinensis Radix*] 9g, *Conioselinum anthriscoides* (H.Boissieu) Pimenov & Kljuykov [Apiaceae, *Ligustici Rhizoma et Radix*] 6g, *Paeonia lactiflora* Pall. [Ranunculaceae, *Paeoniae Radix Rubra*] 6g, *Cyperus rotundus* L. [Cyperaceae, *Cyperi Rhizoma*] 4.5g, *Citrus × aurantium* L. [Rutaceae, *Aurantii Fructus*] 4.5g, *Lindera aggregata* (Sims) Kosterm. [Lauraceae, *Linderae Radix*] 9g, *Trogopterus xanthipes* (Milne-Edwards, 1867) [Sciuridae, *Faeces Trogopterori*] 6g, *Paeonia × suffruticosa* Andrews [Paeoniaceae, *Moutan Cortex*] 6g, *Corydalis yanhusuo* (Y.H.Chou & Chun C.Hsu) W.T.Wang ex Z.Y.Su & C.Y.Wu [Papaveraceae, *Corydalis Rhizoma*] 3g, *Glycyrrhiza uralensis* Fisch. [Fabaceae, *Glycyrrhizae Radix et Rhizoma*] 9g | Decoction |
| Chen, Z (2024) | Huazhuo Jiedu Decoction | *Eupatorium japonicum* Thunb. [Asteraceae, *Eupatorii Herba*] 15g, *Salvia miltiorrhiza* Bunge [Lamiaceae, *Salviae Miltiorrhizae Radix et Rhizoma*] 15g, *Paeonia lactiflora* Pall. [Ranunculaceae, *Paeoniae Radix Rubra*] 15g, *Coix lacryma-jobi* var. *ma-yuen* (Rom.Caill.) Stapf [Poaceae, *Coicis Semen*] 15g, *Atractylodes lancea*(Thunb.) DC. [Asteraceae, *Atractylodis Rhizoma*] 12g, *Smilax glabra* Roxb. [Smilacaceae, *Smilacis Glabrae Rhizoma*] 20g, *Astragalus membranaceus* (Fisch.) Bge. [Fabaceae, *Astragali Radix*] 30g, *Bombyx mori* Linnaeus, 1758 [Bombycidae, *Bombycis Faeces*] 10g | Decoction |
| Zhang, C (2024) | Self-formulated Prescription | *Codonopsis pilosula* (Franch.) Nannf. [Campanulaceae, *Codonopsis Radix*] 12g, *Atractylodes macrocephala* Koidz. [Asteraceae, *Atractylodis Macrocephalae Rhizoma*] 15g, *Poria cocos* (Schw.) Wolf [Polyporaceae, *Poria*] 12g, *Psoralea corylifolia* L. [Fabaceae, *Psoraleae Fructus*] 10g, *Rehmannia glutinosa* (Gaertn.) Libosch. ex DC. [Orobanchaceae, *Rehmanniae Radix*] 18g, *Dioscorea oppositifolia* L. [Dioscoreaceae, *Dioscoreae Rhizoma*] 18g, *Dipsacus asper* Wall. ex DC. [Caprifoliaceae, *Dipsaci Radix*] 10g, *Epimedium brevicornu* Maxim. [Berberidaceae, *Epimedii Folium*] 10g, *Cuscuta australis* R.Br. [Convolvulaceae, *Cuscutae Semen*] 10g, *Leonurus japonicus* Houtt. [Lamiaceae, *Leonuri Herba*] 15g, *Paeonia lactiflora* Pall. [Ranunculaceae, *Paeoniae Radix Rubra*] 10g, *Pinellia ternata* (Thunb.) Makino [Araceae, *Pinelliae Rhizoma Praeparatum*] 8g, *Citrus reticulata* Blanco [Rutaceae, *Citri Reticulatae Pericarpium*] 12g, *Monascus purpureus*Went [Monascaceae, *Monascus Fermentum*] 8g, *Acorus verus* (L.) Raf. [Acoraceae, *Acori Tatarinowii Rhizoma*] 10g, *Glycyrrhiza uralensis* Fisch. [Fabaceae, *Glycyrrhizae Radix et Rhizoma*] 6g, | Other |
| Shen, J (2023) | Dachaihu Decoction combined with Fangji Huangqi Decoction | *Bupleurum chinense* DC. [Apiaceae, *Bupleuri Radix*] 18g, *Scutellaria baicalensis* Georgi [Lamiaceae, *Scutellariae Radix*] 10g, *Pinellia ternata* (Thunb.) Makino [Araceae, *Pinelliae Rhizoma*] 12g, *Citrus × aurantium* L. [Rutaceae, *Aurantii Fructus*] 12g, *Paeonia lactiflora* Pall. [Ranunculaceae, *Paeoniae Radix Alba*] 15g, *Rheum palmatum* L. [Polygonaceae, *Rhei Radix et Rhizoma Praeparata*] 10g, *Zingiber officinale* Roscoe [Zingiberaceae, *Zingiberis Rhizoma*] 5g, *Ziziphus jujuba* Mill. [Rhamnaceae, *Jujubae Fructus*] 15g, *Stephania tetrandra* S.Moore [Menispermaceae, *Stephaniae Radix*] 12g, *Astragalus membranaceus* (Fisch.) Bge. [Fabaceae, *Astragali Radix*] 30g, *Atractylodes macrocephala* Koidz. [Asteraceae, *Atractylodis Macrocephalae Rhizoma*] 15g, *Glycyrrhiza uralensis* Fisch. [Fabaceae, *Glycyrrhizae Radix et Rhizoma*] 5g | Decoction |
| Zhong, Y (2023) | Fangfeng Tongsheng Decoction | *Platycodon grandiflorus* (Jacq.) A.DC. [Campanulaceae, *Platycodonis Radix*] 20g, Talcum [Mg₃Si₄O₁₀(OH)₂, talc] 20g, *Scutellaria baicalensis* Georgi [Lamiaceae, *Scutellariae Radix*] 20g, Gypsum Fibrosum [CaSO₄·2H₂O, gypsum] 20g, *Conioselinum anthriscoides* (H.Boissieu) Pimenov & Kljuykov [Apiaceae, *Ligustici Rhizoma et Radix*] 15g, *Angelica sinensis* (Oliv.) Diels [Apiaceae, *Angelicae Sinensis Radix*] 15g, *Mentha canadensis* L. [Lamiaceae, *Menthae Herba*] 15g, Mirabilitte Purificata [Purified Mineral, Natrii Sulfas] 15g, *Paeonia lactiflora* Pall. [Ranunculaceae, *Paeoniae Radix Alba*] 15g, *Forsythia suspensa* (Thunb.) Vahl [Oleaceae, *Forsythiae Fructus*] 15g, *Saposhnikovia divaricata* (Turcz. ex Ledeb.) Schischk. [Apiaceae, *Saposhnikoviae Radix*] 15g, *Rheum palmatum* L. [Polygonaceae, *Rhei Radix et Rhizoma Praeparata*] 10g, *Glycyrrhiza uralensis* Fisch. [Fabaceae, *Glycyrrhizae Radix et Rhizoma*] 10g, *Atractylodes macrocephala* Koidz. [Asteraceae, *Atractylodis Macrocephalae Rhizoma*] 6g, *Gardenia jasminoides* J.Ellis [Rubiaceae, *Gardeniae Fructus*] 6g, *Actaea heracleifolia* (Kom.) J.Compton [Ranunculaceae, *Cimicifugae Rhizoma*] 6g, *Nepeta tenuifolia* Benth. [Lamiaceae, *Schizonepetae Herba*] 6g | Decoction |
| Shen, Y (2023) | Qutan Lishi Decoction | *Atractylodes lancea* (Thunb.) DC. [Asteraceae, *Atractylodis Rhizoma*] 9g, *Cyperus rotundus* L. [Cyperaceae, *Cyperi Rhizoma*] 9g, *Citrus × aurantium* L. [Rutaceae, *Aurantii Fructus*] 9g, *Citrus reticulata* Blanco [Rutaceae, *Citri Reticulatae Pericarpium*] 9g, *Poria cocos* (Schw.) Wolf [Polyporaceae, *Poria*] 9g, *Pinellia ternata* (Thunb.) Makino [Araceae, *Pinelliae Rhizoma*] 9g, *Atractylodes macrocephala* Koidz. [Asteraceae, *Atractylodis Macrocephalae Rhizoma*] 9g, *Zingiber officinale* Roscoe [Zingiberaceae, *Zingiberis Rhizoma Recens*] 3g, *Taxillus chinensis* (DC.) Danser [Loranthaceae, *Taxilli Herba*] 9g, *Crataegus pinnatifida* Bunge [Rosaceae, *Crataegi Fructus*] 15g, *Prunus persica* (L.) Batsch [Rosaceae, *Persicae Semen*] 9g, *Carthamus tinctorius* L. [Asteraceae, *Carthami Flos*] 9g, *Glycyrrhiza uralensis* Fisch. [Fabaceae, *Glycyrrhizae Radix et Rhizoma*] 9g | Decoction |
| Liu, J (2023b) | Self-formulated Prescription | *Atractylodes lancea* (Thunb.) DC. [Asteraceae, *Atractylodis Rhizoma*] 20g, *Pinellia ternata* (Thunb.) Makino [Araceae, *Pinelliae Rhizoma Praeparatum cum Alumine*] 15g, Citrus reticulata Blanco [Rutaceae, Citri Reticulatae Pericarpium] 15g, Poria cocos (Schw.) Wolf [Polyporaceae, Poria] 15g, *Coix lacryma-jobi* var. *ma-yuen* (Rom.Caill.) Stapf [Poaceae, *Coicis Semen*] 20g, Atractylodes macrocephala Koidz. [Asteraceae, Atractylodis Macrocephalae Rhizoma] 15g, Fritillaria thunbergii Miq. [Liliaceae, Fritillariae Thunbergii Bulbus] 20g, Crataegus pinnatifida Bunge [Rosaceae, Crataegi Fructus] 15g, Cyathula officinalis K.C.Kuan [Amaranthaceae, Cyathulae Radix] 20g, Salvia miltiorrhiza Bunge [Lamiaceae, Salviae Miltiorrhizae Radix et Rhizoma] 20g, Angelica sinensis (Oliv.) Diels [Apiaceae, Angelicae Sinensis Radix] 15g, Conioselinum anthriscoides (H.Boissieu) Pimenov & Kljuykov [Apiaceae, Ligustici Rhizoma et Radix] 15g | Decoction |
| Fu, C (2023) | Yinang Jianzhi Decoction | *Atractylodes lancea* (Thunb.) DC. [Asteraceae, *Atractylodis Rhizoma*] 15g, *Cyperus rotundus* L. [Cyperaceae, *Cyperi Rhizoma*] 15g, *Prunus persica* (L.) Batsch [Rosaceae, *Persicae Semen*] 10g, *Citrus reticulata* Blanco [Rutaceae, *Citri Reticulatae Pericarpium*] 10g, *Citrus × aurantium* L. [Rutaceae, *Aurantii Fructus*] 10g, *Magnolia officinalis* Rehder & E.H.Wilson [Magnoliaceae, *Magnoliae Officinalis Cortex*] 10g, *Dioscorea oppositifolia* L. [Dioscoreaceae, *Dioscoreae Rhizoma*] 10g, Fluoritum (Calcium Fluoride, CaF₂) 10g, *Cervus nippon* Temminck, 1838 [Cervidae, *Cervi Cornu Praeparatum*] 15g, *Pinellia ternata* (Thunb.) Makino [Araceae, *Pinelliae Rhizoma Praeparatum*] 15g, *Arisaema erubescens* (Wall.) Schott [Araceae, *Arisaema cum Bile*] 12g, *Crataegus pinnatifida* Bunge [Rosaceae, *Crataegi Fructus*] 10g, *Monascus purpureus* Went [Monascaceae, *Monascus Fermentum*] 6g | Decoction |
| Liu, J (2023a) | Modified Danxi Zhishitan Decoction | *Atractylodes lancea* (Thunb.) DC. [Asteraceae, *Atractylodis Rhizoma*] 15g, *Pinellia ternata* (Thunb.) Makino [Araceae, *Pinelliae Rhizoma Praeparatum*] 15g, *Atractylodes macrocephala* Koidz. [Asteraceae, *Atractylodis Macrocephalae Rhizoma*] 10g, *Poria cocos* (Schw.) Wolf [Polyporaceae, *Poria*] 10g, Talcum [Mg₃Si₄O₁₀(OH)₂, talc] 6g, *Cyperus rotundus* L. [Cyperaceae, *Cyperi Rhizoma*] 6g, *Conioselinum anthriscoides* (H.Boissieu) Pimenov & Kljuykov [Apiaceae, *Ligustici Rhizoma et Radix*] 6g, *Angelica sinensis* (Oliv.) Diels [Apiaceae, *Angelicae Sinensis Radix*] 6g | Decoction |
| Zhao, C (2023) | Bushen Huatan Decoction | *Rehmannia glutinosa* (Gaertn.) Libosch. ex DC. [Orobanchaceae, *Rehmanniae Radix*] 25g, *Poria cocos* (Schw.) Wolf [Polyporaceae, *Poria*] 15g, *Dioscorea oppositifolia* L. [Dioscoreaceae, *Dioscoreae Rhizoma*] 15g, *Coix lacryma-jobi* var. *ma-yuen* (Rom.Caill.) Stapf [Poaceae, *Coicis Semen*] 25g, *Euryale ferox* Salisb. [Nymphaeaceae, Euryales Semen] 25g, *Cornus officinalis* Siebold & Zucc. [Cornaceae, *Corni Fructus*] 15g, *Schisandra chinensis* (Turcz.) Baill. [Schisandraceae, *Schisandrae Fructus*] 5g, *Ophiopogon japonicus* (L.f) Ker-Gawl. [Liliaceae, *Ophiopogonis Radix*] 15g, *Plantago asiatica* L. [Plantaginaceae, *Plantago Semen*] 5g, *Alpinia oxyphylla* Miq. [Zingiberaceae, *Alpiniae Oxyphyllae Fructus*] 5g | Decoction |
| Zhang, H (2023a) | Qigong Decoction | *Citrus reticulata* Blanco [Rutaceae, *Citri Reticulatae* Pericarpium], *Pinellia ternata* (Thunb.) Makino [Araceae, *Pinelliae Rhizoma*], *Poria cocos* (Schw.) Wolf [Polyporaceae, *Poria*], *Atractylodes macrocephala* Koidz. [Asteraceae, *Atractylodis Macrocephalae Rhizoma*], *Cyperus rotundus* L. [Cyperaceae, *Cyperi Rhizoma*], *Conioselinum anthriscoides* (H.Boissieu) Pimenov & Kljuykov [Apiaceae, *Ligustici Rhizoma et Radix*], Massa Medicata Fermentata, *Glycyrrhiza uralensis* Fisch. [Fabaceae, *Glycyrrhizae Radix et Rhizoma*] | Decoction |
| Shan, K (2022) | Cangfu Daotan Decoction | *Astragalus membranaceus* (Fisch.) Bge. [Fabaceae, *Astragali Radix*] 30g, *Dioscorea oppositifolia* L. [Dioscoreaceae, *Dioscoreae Rhizoma*] 30g, *Poria cocos* (Schw.) Wolf [Polyporaceae, *Poria*] 30g, *Salvia miltiorrhiza* Bunge [Lamiaceae, *Salviae Miltiorrhizae Radix et Rhizoma*] 15g, *Epimedium brevicornu*Maxim. [Berberidaceae, *Epimedii Folium*] 15g, *Gleditsia sinensis* Lam. [Fabaceae, *Gleditsiae Spina*] 10g, *Cyperus rotundus*L. [Cyperaceae, *Cyperi Rhizoma*] 10g, *Pinellia ternata* (Thunb.) Makino [Araceae, *Pinelliae Rhizoma Praeparatum*] 10g, *Acorus verus* (L.) Raf. [Acoraceae, *Acori Tatarinowii Rhizoma*] 10g, *Atractylodes lancea*(Thunb.) DC. [Asteraceae, *Atractylodis Rhizoma*] 10g, *Angelica sinensis* (Oliv.) Diels [Apiaceae, *Angelicae Sinensis Radix*] 10g, *Citrus reticulata* Blanco [Rutaceae, *Citri Reticulatae Pericarpium*] 6g | Decoction |
| Zhang, Y (2022b) | Cangfu Daotan Decoction | *Atractylodes lancea* (Thunb.) DC. [Asteraceae, *Atractylodis Rhizoma*] 20g, *Cyperus rotundus*L. [Cyperaceae, *Cyperi Rhizoma*] 20g, *Citrus reticulata* Blanco [Rutaceae, *Citri Reticulatae Pericarpium*] 15g, *Poria cocos* (Schw.) Wolf [Polyporaceae, *Poria*] 15g, *Arisaema erubescens*(Wall.) Schott [Araceae, *Arisaematis Rhizoma*] 10g, *Citrus × aurantium* L. [Rutaceae, *Aurantii Fructus*] 10g, *Pinellia ternata* (Thunb.) Makino [Araceae, *Pinelliae Rhizoma*] 10g, *Conioselinum anthriscoides* (H.Boissieu) Pimenov & Kljuykov [Apiaceae, *Ligustici Rhizoma et Radix*] 10g, Massa Medicata Fermentata 10g | Decoction |
| Tang, J (2022) | Ditan Zhuyu Decoction | *Citrus reticulata* Blanco [Rutaceae, *Citri Reticulatae Pericarpium*], *Poria cocos* (Schw.) Wolf [Polyporaceae, *Poria*], *Pinellia ternata* (Thunb.) Makino [Araceae, *Pinelliae Rhizoma Praeparatum*], *Crataegus pinnatifida* Bunge [Rosaceae, *Crataegi Fructus*], *Acorus verus* (L.) Raf. [Acoraceae, *Acori Tatarinowii Rhizoma*], *Arisaema erubescens* (Wall.) Schott [Araceae, *Arisaema cum Bile*], *Sparganium stoloniferum* (Buch.-Ham. ex Graebn.) Buch.-Ham. ex Juz. [Typhaceae, *Sparganii Rhizoma*], *Curcuma phaeocaulis* Valeton [Zingiberaceae, *Curcumae Rhizoma*], *Epimedium brevicornu* Maxim. [Berberidaceae, *Epimedii Folium*], *Gynochthodes officinalis* (F.C.How) Razafim. & B.Bremer [Rubiaceae, *Morindae Officinalis Radix*], *Spatholobus suberectus* Dunn [Fabaceae, *Spatholobi Caulis*], *Neolitsea cassia* (L.) Kosterm. [Lauraceae, *Cinnamomi Cortex*] | Decoction |
| Jiang, X (2022) | Gexia Zhuyu Decoction | *Prunus persica* (L.) Batsch [Rosaceae, *Persicae Semen*] 9g, *Carthamus tinctorius* L. [Asteraceae, *Carthami Flos*] 9g, *Angelica sinensis* (Oliv.) Diels [Apiaceae, *Angelicae Sinensis Radix*] 9g, *Conioselinum anthriscoides* (H.Boissieu) Pimenov & Kljuykov [Apiaceae, *Ligustici Rhizoma et Radix*] 6g, *Paeonia lactiflora* Pall. [Ranunculaceae, *Paeoniae Radix Rubra*] 6g, *Cyperus rotundus* L. [Cyperaceae, *Cyperi Rhizoma*] 4.5g, *Citrus × aurantium* L. [Rutaceae, *Aurantii Fructus*] 4.5g, *Lindera aggregata* (Sims) Kosterm. [Lauraceae, *Linderae Radix*] 9g, *Trogopterus xanthipes* (Milne-Edwards, 1867) [Sciuridae, *Faeces Trogopterori*] 6g, *Corydalis yanhusuo* (Y.H.Chou & Chun C.Hsu) W.T.Wang ex Z.Y.Su & C.Y.Wu [Papaveraceae, *Corydalis Rhizoma*] 3g, *Paeonia × suffruticosa* Andrews [Paeoniaceae, *Moutan Cortex*] 6g, *Glycyrrhiza uralensis* Fisch. [Fabaceae, *Glycyrrhizae Radix et Rhizoma*] 9g | Decoction |
| Cui, M (2022) | Bushen Huatan Decoction | *Epimedium brevicornu* Maxim. [Berberidaceae, *Epimedii Folium*] 10g, *Curculigo orchioides* Gaertn. [Hypoxidaceae, *Curculiginis Rhizoma*] 10g, *Atractylodes lancea* (Thunb.) DC. [Asteraceae, *Atractylodis Rhizoma*] 10g, *Pinellia ternata* (Thunb.) Makino [Araceae, *Pinelliae Rhizoma*] 6g, *Citrus reticulata* Blanco [Rutaceae, *Citri Reticulatae Pericarpium*] 6g, *Anemone altaica* Fisch. ex C.A.Mey. [Ranunculaceae, *Anemones Rhizoma*] 10g, *Cyperus rotundus*L. [Cyperaceae, *Cyperi Rhizoma*] 10g, *Conioselinum anthriscoides* (H.Boissieu) Pimenov & Kljuykov [Apiaceae, *Ligustici Rhizoma et Radix*] 6g, *Alisma plantago-aquatica* subsp. *orientale* (Sam.) Sam. [Alismataceae, *Alismatis Rhizoma*] 10g, *Cervus nippon* Temminck, 1838 [Cervidae, *Cervi Cornu Praeparatum*] 10g, *Arisaema erubescens* (Wall.) Schott [Araceae, *Arisaema cum Bile*] 6g, *Wurfbainia villosa* (Lour.) Škorničk. & A.D.Poulsen [Zingiberaceae, *Amomi Fructus*] 3g, *Atractylodes macrocephala* Koidz. [Asteraceae, *Atractylodis Macrocephalae Rhizoma*] 6g, *Dioscorea oppositifolia* L. [Dioscoreaceae, *Dioscoreae Rhizoma*] 6g | Decoction |
| Wei, X (2022) | Bushen Huoxue Decoction | *Rehmannia glutinosa* (Gaertn.) Libosch. ex DC. [Orobanchaceae, *Rehmanniae Radix*] 20g, Fluoritum (Calcium Fluoride, CaF₂) 20g, *Codonopsis pilosula* (Franch.) Nannf. [Campanulaceae, *Codonopsis Radix*] 20g, *Cuscuta australis* R.Br. [Convolvulaceae, *Cuscutae Semen*] 15g, *Lycium barbarum* L. [Solanaceae, *Lycii Fructus*] 15g, *Epimedium brevicornu* Maxim. [Berberidaceae, *Epimedii Folium*] 15g, *Morus alba* L. [Moraceae, *Mori Fructus*] 15g, *Angelica sinensis* (Oliv.) Diels [Apiaceae, *Angelicae Sinensis Radix*] 10g, *Ligustrum lucidum* W.T.Aiton [Oleaceae, *Ligustri Lucidi Fructus*] 10g, *Cervus nippon* Temminck, 1838 [Cervidae, *Cervi Cornu*] 10g, *Scleromitrion diffusum* (Willd.) R.J.Wang [Rubiaceae, *Hedyotis Herba*] 10g, *Conioselinum anthriscoides* (H.Boissieu) Pimenov & Kljuykov [Apiaceae, *Ligustici Rhizoma et Radix*] 10g, *Cyperus rotundus* L. [Cyperaceae, *Cyperi Rhizoma*] 10g, *Glycyrrhiza uralensis* Fisch. [Fabaceae, *Glycyrrhizae Radix et Rhizoma*] 10g, *Carthamus tinctorius* L. [Asteraceae, *Carthami Flos*] 6g | Decoction |
| Sun, M (2022) | Huoxue Qushi Bushen Decoction | Fluoritum (Calcium Fluoride, CaF₂) 20g, *Crataegus pinnatifida* Bunge [Rosaceae, *Crataegi Fructus*] 20g, *Epimedium brevicornu* Maxim. [Berberidaceae, *Epimedii Folium*] 15g, *Gynochthodes officinalis* (F.C.How) Razafim. & B.Bremer [Rubiaceae, *Morindae Officinalis Radix*] 15g, *Cuscuta australis*R.Br. [Convolvulaceae, *Cuscutae Semen*] 15g, *Atractylodes lancea* (Thunb.) DC. [Asteraceae, *Atractylodis Rhizoma*] 15g, *Cyperus rotundus*L. [Cyperaceae, *Cyperi Rhizoma*] 15g, *Poria cocos* (Schw.) Wolf [Polyporaceae, *Poria*] 15g, *Cyathula officinalis* K.C.Kuan [Amaranthaceae, *Cyathulae Radix*] 15g, *Citrus reticulata* Blanco [Rutaceae, *Citri Reticulatae Pericarpium*] 12g, *Cervus nippon* Temminck, 1838 [Cervidae, *Cervi Cornu Praeparatum*] 12g, *Pinellia ternata* (Thunb.) Makino [Araceae, *Pinelliae Rhizoma Praeparatum cum Alumine*] 12g, *Gleditsia sinensis* Lam. [Fabaceae, *Gleditsiae Spina*] 12g, *Angelica sinensis* (Oliv.) Diels [Apiaceae, *Angelicae Sinensis Radix*] 12g, *Zanthoxylum bungeanum* Maxim. [Rutaceae, *Zanthoxyli Pericarpium*] 3g, *Glycyrrhiza uralensis* Fisch. [Fabaceae, *Glycyrrhizae Radix et Rhizoma*] 6g | Decoction |
| Zeng, Q (2022) | Cupailuan Decoction | *Angelica sinensis* (Oliv.) Diels [Apiaceae, *Angelicae Sinensis Radix*] 10g, *Cuscuta australis* R.Br. [Convolvulaceae, *Cuscutae Semen*] 10g, *Atractylodes macrocephala* Koidz. [Asteraceae, *Atractylodis Macrocephalae Rhizoma*] 10g, *Conioselinum anthriscoides* (H.Boissieu) Pimenov & Kljuykov [Apiaceae, *Ligustici Rhizoma et Radix*] 10g, *Paeonia × suffruticosa* Andrews [Paeoniaceae, *Moutan Cortex*] 10g, *Salvia miltiorrhiza* Bunge [Lamiaceae, *Salviae Miltiorrhizae Radix et Rhizoma*] 10g, *Coix lacryma-jobi* var. *ma-yuen* (Rom.Caill.) Stapf [Poaceae, *Coicis Semen*] 10g, *Dipsacus asper* Wall. ex DC. [Caprifoliaceae, *Dipsaci Radix*] 15g, *Paeonia lactiflora* Pall. [Ranunculaceae, *Paeoniae Radix Rubra*] 15g, *Atractylodes lancea* (Thunb.) DC. [Asteraceae, *Atractylodis Rhizoma*] 15g, *Poria cocos* (Schw.) Wolf [Polyporaceae, *Poria*] 15g | Decoction |
| Cheng, T (2022) | Fenxiao Huoxue Decoction | *Prunus armeniaca* var. *armeniaca* [Rosaceae, *Armeniacae Semen*] 15g, *Wurfbainia vera* (Blackw.) Škorničk. & A.D.Poulsen [Zingiberaceae, *Amomi Fructus*] 15g, *Citrus × aurantium* L. [Rutaceae, *Aurantii Fructus*] 15g, *Pinellia ternata* (Thunb.) Makino [Araceae, *Pinelliae Rhizoma Praeparatum*] 10g, *Plantago asiatica* L. [Plantaginaceae, *Plantago Semen*] 10g, *Coix lacryma-jobi* var. *ma-yuen* (Rom.Caill.) Stapf [Poaceae, *Coicis Semen*] 10g, *Citrus reticulata* Blanco [Rutaceae, *Citri Reticulatae Pericarpium*] 10g, *Magnolia officinalis* Rehder & E.H.Wilson [Magnoliaceae, *Magnoliae Officinalis Cortex*] 10g, *Coptis chinensis* Franch. [Ranunculaceae, *Coptidis Rhizoma*] 10g, *Poria cocos* (Schw.) Wolf [Polyporaceae, *Poria*] 10g, *Scutellaria baicalensis* Georgi [Lamiaceae, *Scutellariae Radix*] 15g, *Tetrapanax papyrifer* (Hook.) K.Koch [Araliaceae, *Tetrapanacis Medulla*] 10g, *Cyperus rotundus* L. [Cyperaceae, *Cyperi Rhizoma*] 15g, *Conioselinum anthriscoides* (H.Boissieu) Pimenov & Kljuykov [Apiaceae, *Ligustici Rhizoma et Radix*] 15g, *Achyranthes bidentata* Blume [Amaranthaceae, *Achyranthis Bidentatae Radix*] 15g, *Paeonia lactiflora* Pall. [Ranunculaceae, *Paeoniae Radix Rubra*] 15g | Decoction |
| Zhou, T (2021) | Jianpi Huatan Decoction | *Poria cocos* (Schw.) Wolf [Polyporaceae, *Poria*] 15g, *Fritillaria thunbergii* Miq. [Liliaceae, *Fritillariae Thunbergii Bulbus*] 15g, *Astragalus membranaceus* (Fisch.) Bge. [Fabaceae, *Astragali Radix*] 10g, *Bupleurum chinense* DC. [Apiaceae, *Bupleuri Radix*] 10g, *Angelica sinensis* (Oliv.) Diels [Apiaceae, *Angelicae Sinensis Radix*] 10g, *Atractylodes lancea* (Thunb.) DC. [Asteraceae, *Atractylodis Rhizoma*] 10g, *Citrus reticulata* Blanco [Rutaceae, *Citri Reticulatae Pericarpium*] 10g, *Pinellia ternata* (Thunb.) Makino [Araceae, *Pinelliae Rhizoma Praeparatum*] 10g, *Acorus verus* (L.) Raf. [Acoraceae, *Acori Tatarinowii Rhizoma*] 10g, *Citrus × aurantium* L. [Rutaceae, *Aurantii Fructus*] 10g, *Coptis chinensis* Franch. [Ranunculaceae, *Coptidis Rhizoma*] 5g, *Conioselinum anthriscoides* (H.Boissieu) Pimenov & Kljuykov [Apiaceae, *Ligustici Rhizoma et Radix*] 5g, *Glycyrrhiza uralensis* Fisch. [Fabaceae, *Glycyrrhizae Radix et Rhizoma*] 3g | Decoction |
| Xu, Y (2021) | Modified Huanglian Wendan Decoction | *Coptis chinensis* Franch. [Ranunculaceae, *Coptidis Rhizoma*] 6g, *Poria cocos* (Schw.) Wolf [Polyporaceae, *Poria*] 12g, *Pinellia ternata* (Thunb.) Makino [Araceae, *Pinelliae Rhizoma Praeparatum cum Zingibere et Alumine*] 9g, *Bambusa tuldoides* Munro [Poaceae, *Bambusae Caulis in Taenias*] 12g, *Citrus × aurantium* L. [Rutaceae, *Aurantii Fructus*] 12g, *Citrus reticulata* Blanco [Rutaceae, *Citri Reticulatae Pericarpium*] 12g, *Cuscuta australis* R.Br. [Convolvulaceae, *Cuscutae Semen*] 12g, *Arisaema erubescens* (Wall.) Schott [Araceae, *Arisaema cum Bile*] 9g, *Trichosanthes kirilowii* Maxim. [Cucurbitaceae, *Trichosanthis Fructus*] 12g, *Cyperus rotundus* L. [Cyperaceae, *Cyperi Rhizoma*] 12g, *Taxillus chinensis* (DC.) Danser [Loranthaceae, *Taxilli Herba*] 9g, *Glycyrrhiza uralensis* Fisch. [Fabaceae, *Glycyrrhizae Radix et Rhizoma*] 6g | Decoction |
| Zhou, G (2021) | Qigong Pills Modified Decotion | *Citrus reticulata* Blanco [Rutaceae, *Citri Reticulatae Pericarpium*] 12g, *Pinellia ternata* (Thunb.) Makino [Araceae, *Pinelliae Rhizoma*] 9g, *Poria cocos* (Schw.) Wolf [Polyporaceae, *Poria*] 20g, *Glycyrrhiza uralensis* Fisch. [Fabaceae, *Glycyrrhizae Radix et Rhizoma*] 6g, *Atractylodes macrocephala* Koidz. [Asteraceae, *Atractylodis Macrocephalae Rhizoma*] 15g, *Cyperus rotundus* L. [Cyperaceae, *Cyperi Rhizoma*] 12g, Massa Medicata Fermentata 15g, *Atractylodes lancea* (Thunb.) DC. [Asteraceae, *Atractylodis Rhizoma*] 15g, *Gallus gallus* subsp. *domesticus* (Linnaeus, 1758) [Phasianidae, *Endothelium Corneum Gigeriae Galli*] 30g, *Spatholobus suberectus* Dunn [Fabaceae, *Spatholobi Caulis*] 30g, *Conioselinum anthriscoides* (H.Boissieu) Pimenov & Kljuykov [Apiaceae, *Ligustici Rhizoma et Radix*] 9g, *Angelica sinensis* (Oliv.) Diels [Apiaceae, *Angelicae Sinensis Radix*] 12g, *Leonurus japonicus* Houtt. [Lamiaceae, *Leonuri Herba*] 15g, *Lycopus lucidus* Turcz. ex Benth. [Lamiaceae, *Lycopi Herba*] 15g, *Coix lacryma-jobi* var. *ma-yuen* (Rom.Caill.) Stapf [Poaceae, *Coicis Semen*] 30g, *Crataegus pinnatifida* Bunge [Rosaceae, *Crataegi Fructus*] 15g, *Tribulus terrestris* L. [Zygophyllaceae, *Tribuli Fructus*] 15g | Granula |
| Ren, Y (2021) | Shoushen Tiaojing Decoction | *Atractylodes lancea* (Thunb.) DC. [Asteraceae, *Atractylodis Rhizoma*] 10g, *Cyperus rotundus* L. [Cyperaceae, *Cyperi Rhizoma*] 10g, *Poria cocos* (Schw.) Wolf [Polyporaceae, *Poria*] 10g, *Citrus reticulata* Blanco [Rutaceae, *Citri Reticulatae Pericarpium*] 6g, *Pinellia ternata* (Thunb.) Makino [Araceae, *Pinelliae Rhizoma*] 6g, *Citrus × aurantium* L. [Rutaceae, *Aurantii Fructus*] 6g, *Leonurus japonicus* Houtt. [Lamiaceae, *Leonuri Fructus*] 10g, *Taxillus chinensis* (DC.) Danser [Loranthaceae, *Taxilli Herba*] 10g, *Crataegus pinnatifida* Bunge [Rosaceae, *Crataegi Fructus*] 10g, *Salvia miltiorrhiza* Bunge [Lamiaceae, *Salviae Miltiorrhizae Radix et Rhizoma*] 10g, *Plantago asiatica* L. [Plantaginaceae, *Plantago Semen*] 15g, *Achyranthes bidentata*Blume [Amaranthaceae, *Achyranthis Bidentatae Radix*] 10g, *Glycyrrhiza uralensis* Fisch. [Fabaceae, *Glycyrrhizae Radix et Rhizoma*] 3g | Granula |
| Lin, Z (2021) | Modified Pingwei San | *Atractylodes lancea* (Thunb.) DC. [Asteraceae, *Atractylodis Rhizoma*] 16g, *Magnolia officinalis* Rehder & E.H.Wilson [Magnoliaceae, *Magnoliae Officinalis Cortex*] 12g, *Citrus reticulata* Blanco [Rutaceae, *Citri Reticulatae Pericarpium*] 10g, *Zingiber officinale* Roscoe [Zingiberaceae, *Zingiberis Rhizoma Recens*] 10g, *Bupleurum chinense* DC. [Apiaceae, *Bupleuri Radix*] 10g, *Paeonia lactiflora* Pall. [Ranunculaceae, *Paeoniae Radix Alba*] 15g, *Citrus × aurantium* L. [Rutaceae, *Aurantii Fructus*] 10g, *Glycyrrhiza uralensis* Fisch. [Fabaceae, *Glycyrrhizae Radix et Rhizoma*] 6g, *Ziziphus jujuba* Mill. [Rhamnaceae, *Jujubae Fructus*] 15g, *Eupatorium japonicum* Thunb. [Asteraceae, *Eupatorii Herba*] 15g, *Sinapis alba* L. [Brassicaceae, *Sinapis Semen*] 15g, *Cuscuta australis* R.Br. [Convolvulaceae, *Cuscutae Semen*] 30g, *Euonymus alatus* (Thunb.) Siebold [Celastraceae, *Euonymi Ramulus*] 15g | Granula |
| Li, J (2021) | Jianpi Huatan Decoction | *Poria cocos* (Schw.) Wolf [Polyporaceae, *Poria*] 15g, *Fritillaria thunbergii* Miq. [Liliaceae, *Fritillariae Thunbergii Bulbus*] 15g, *Astragalus membranaceus* (Fisch.) Bge. [Fabaceae, *Astragali Radix*] 10g, *Bupleurum chinense* DC. [Apiaceae, *Bupleuri Radix*] 10g, *Angelica sinensis* (Oliv.) Diels [Apiaceae, *Angelicae Sinensis Radix*] 10g, *Atractylodes lancea* (Thunb.) DC. [Asteraceae, *Atractylodis Rhizoma*] 10g, *Citrus reticulata* Blanco [Rutaceae, *Citri Reticulatae Pericarpium*] 10g, *Pinellia ternata* (Thunb.) Makino [Araceae, *Pinelliae Rhizoma Praeparatum*] 10g, *Acorus verus* (L.) Raf. [Acoraceae, *Acori Tatarinowii Rhizoma*] 10g, *Citrus × aurantium* L. [Rutaceae, *Aurantii Fructus*] 10g, *Coptis chinensis* Franch. [Ranunculaceae, *Coptidis Rhizoma*] 5g, *Conioselinum anthriscoides* (H.Boissieu) Pimenov & Kljuykov [Apiaceae, *Ligustici Rhizoma et Radix*] 5g, *Glycyrrhiza uralensis* Fisch. [Fabaceae, *Glycyrrhizae Radix et Rhizoma*] 3g | Decoction |
| Ge, R (2021) | Modified Erchen Decoction combined with Gegen Decoction | *Citrus reticulata* Blanco [Rutaceae, *Citri Reticulatae Pericarpium*] 12g, *Poria cocos* (Schw.) Wolf [Polyporaceae, *Poria*] 15g, *Pinellia ternata* (Thunb.) Makino [Araceae, *Pinelliae Rhizoma Praeparatum cum Alumine*] 12g, *Rehmannia glutinosa* (Gaertn.) Libosch. ex DC. [Orobanchaceae, *Rehmanniae Radix*] 10g, *Cornus officinalis* Siebold & Zucc. [Cornaceae, *Corni Fructus*] 10g, *Cuscuta australis* R.Br. [Convolvulaceae, *Cuscutae Semen*] 12g, *Angelica sinensis* (Oliv.) Diels [Apiaceae, *Angelicae Sinensis Radix*] 15g, *Ephedra sinica* Stapf [Ephedraceae, *Ephedrae Herba*] 5g, *Neolitsea cassia* (L.) Kosterm. [Lauraceae, *Cinnamomi Cortex*] 12g, *Pueraria montana* var. *lobata* (Willd.) Maesen & S.M.Almeida ex Sanjappa & Predeep [Fabaceae, *Puerariae Radix*] 15g, *Hansenia weberbaueriana* (Fedde ex H.Wolff) Pimenov & Kljuykov [Apiaceae, *Hanseniae Radix*] 10g, *Zingiber officinale* Roscoe [Zingiberaceae, *Zingiberis Rhizoma Recens*] 5g, *Glycyrrhiza uralensis* Fisch. [Fabaceae, *Glycyrrhizae Radix et Rhizoma*] 6g | Decoction |
| Tang, Y (2021) | Yinang Zhuyun Decoction | *Atractylodes lancea* (Thunb.) DC. [Asteraceae, *Atractylodis Rhizoma*] 15g, *Citrus × aurantium* L. [Rutaceae, *Aurantii Fructus*] 10g, *Cyperus rotundus* L. [Cyperaceae, *Cyperi Rhizoma*] 15g, *Prunus persica* (L.) Batsch [Rosaceae, *Persicae Semen*] 10g, *Citrus reticulata* Blanco [Rutaceae, *Citri Reticulatae Pericarpium*] 10g, *Magnolia officinalis* Rehder & E.H.Wilson [Magnoliaceae, *Magnoliae Officinalis Cortex*] 10g, *Pinellia ternata* (Thunb.) Makino [Araceae, *Pinelliae Rhizoma Praeparatum*] 15g, *Arisaema erubescens* (Wall.) Schott [Araceae, *Arisaema cum Bile*] 12g, *Crataegus pinnatifida* Bunge [Rosaceae, *Crataegi Fructus*] 10g, *Monascus purpureus* Went [Monascaceae, *Monascus Fermentum*] 6g, Fluoritum (Calcium Fluoride, CaF₂) 10g, *Cervus nippon* Temminck, 1838 [Cervidae, *Cervi Cornu Praeparatum*] 15g | Decoction |
| Fang, S (2021) | Shenling Baizhu Powder | *Panax ginseng* C.A.Mey. [Araliaceae, *Ginseng Radix et Rhizoma*] 10g, *Atractylodes macrocephala* Koidz. [Asteraceae, *Atractylodis Macrocephalae Rhizoma*] 15g, *Poria cocos* (Schw.) Wolf [Polyporaceae, *Poria*] 20g, *Platycodon grandiflorus* (Jacq.) A.DC. [Campanulaceae, *Platycodonis Radix*] 15g, *Nelumbo nucifera* Gaertn. [Nelumbonaceae, *Nelumbinis Semen*] 15g, *Coix lacryma-jobi* var. *ma-yuen* (Rom.Caill.) Stapf [Poaceae, *Coicis Semen*] 20g, *Dioscorea oppositifolia* L. [Dioscoreaceae, *Dioscoreae Rhizoma*] 30g, *Lablab purpureus* subsp. *purpureus* [Fabaceae, *Lablab Semen Album*] 15g, *Glycyrrhiza uralensis* Fisch. [Fabaceae, *Glycyrrhizae Radix et Rhizoma*] 5g | Granula |
| Ben, Q (2021) | Yishen Xiaotan Decoction | *Cuscuta australis* R.Br. [Convolvulaceae, *Cuscutae Semen*] 20g, *Rehmannia glutinosa* (Gaertn.) Libosch. ex DC. [Orobanchaceae, *Rehmanniae Radix*] 20g, *Taxillus chinensis* (DC.) Danser [Loranthaceae, *Taxilli Herba*] 15g, *Atractylodes macrocephala* Koidz. [Asteraceae, *Atractylodis Macrocephalae Rhizoma*] 12g, *Lablab purpureus* subsp. *purpureus* [Fabaceae, *Lablab Semen Album*] 15g, *Poria cocos* (Schw.) Wolf [Polyporaceae, *Poria*] 15g, *Citrus reticulata* Blanco [Rutaceae, *Citri Reticulatae Pericarpium*] 10g, *Salvia miltiorrhiza* Bunge [Lamiaceae, *Salviae Miltiorrhizae Radix et Rhizoma*] 20g, *Angelica sinensis* (Oliv.) Diels [Apiaceae, *Angelicae Sinensis Radix*] 12g, *Spatholobus suberectus* Dunn [Fabaceae, *Spatholobi Caulis*] 15g, *Cyperus rotundus* L. [Cyperaceae, *Cyperi Rhizoma*] 12g, *Lycopus lucidus* Turcz. ex Benth. [Lamiaceae, *Lycopi Herba*] 12g, *Plantago asiatica* L. [Plantaginaceae, *Plantago Semen*] 15g, *Benincasa hispida* (Thunb.) Cogn. [Cucurbitaceae, *Benincasae Exocarpium*] 15g, *Phragmites australis* subsp. *australis* [Poaceae, *Phragmitis Rhizoma*] 12g, *Pueraria montana* var. *lobata* (Willd.) Maesen & S.M.Almeida ex Sanjappa & Predeep [Fabaceae, *Puerariae Radix*] 12g | Decoction |
| Fan, J (2021) | Modified Cangfu Daotan Decoction | *Atractylodes lancea* (Thunb.) DC. [Asteraceae, *Atractylodis Rhizoma*], *Cyperus rotundus* L. [Cyperaceae, *Cyperi Rhizoma*], *Citrus × aurantium* L. [Rutaceae, *Aurantii Fructus*], *Citrus reticulata* Blanco [Rutaceae, *Citri Reticulatae Pericarpium*], *Poria cocos* (Schw.) Wolf [Polyporaceae, *Poria*], *Glycyrrhiza uralensis* Fisch. [Fabaceae, *Glycyrrhizae Radix et Rhizoma*], *Zingiber officinale* Roscoe [Zingiberaceae, *Zingiberis Rhizoma Recens*], Massa Medicata Fermentata, *Pinellia ternata* (Thunb.) Makino [Araceae, *Pinelliae Rhizoma Praeparatum*], *Angelica sinensis* (Oliv.) Diels [Apiaceae, *Angelicae Sinensis Radix*], *Gynochthodes officinalis* (F.C.How) Razafim. & B.Bremer [Rubiaceae, *Morindae Officinalis Radix*], *Lycium barbarum* L. [Solanaceae, *Lycii Fructus*], *Cuscuta australis* R.Br. [Convolvulaceae, *Cuscutae Semen*], *Epimedium brevicornu* Maxim. [Berberidaceae, *Epimedii Folium*], *Eucommia ulmoides* Oliv. [Eucommiaceae, *Eucommiae Cortex*] | Other |
| Wang, Z (2021) | Self-formulated Prescription | *Arisaema erubescens* (Wall.) Schott [Araceae, *Arisaema cum Bile*] 15g, *Atractylodes lancea* (Thunb.) DC. [Asteraceae, *Atractylodis Rhizoma*] 20g, *Citrus × aurantium* L. [Rutaceae, *Aurantii Fructus*] 15g, *Cyperus rotundus* L. [Cyperaceae, *Cyperi Rhizoma*] 10g, *Pinellia ternata* (Thunb.) Makino [Araceae, *Pinelliae Rhizoma*] 15g, *Citrus reticulata* Blanco [Rutaceae, *Citri Reticulatae Pericarpium*] 10g, *Poria cocos* (Schw.) Wolf [Polyporaceae, *Poria*] 15g, *Zingiber officinale* Roscoe [Zingiberaceae, *Zingiberis Rhizoma Recens*] 10g, *Glycyrrhiza uralensis* Fisch. [Fabaceae, *Glycyrrhizae Radix et Rhizoma*] 10g | Decoction |
| He, X (2021) | Jianpi Bushen Huoxue Decoction | *Codonopsis pilosula* (Franch.) Nannf. [Campanulaceae, *Codonopsis Radix*] 15g, *Angelica sinensis* (Oliv.) Diels [Apiaceae, *Angelicae Sinensis Radix*] 15g, *Salvia miltiorrhiza* Bunge [Lamiaceae, *Salviae Miltiorrhizae Radix et Rhizoma*] 15g, *Psoralea corylifolia* L. [Fabaceae, *Psoraleae Fructus*] 12g, *Dioscorea oppositifolia* L. [Dioscoreaceae, *Dioscoreae Rhizoma*] 12g, *Dipsacus asper*Wall. ex DC. [Caprifoliaceae, *Dipsaci Radix*] 12g, *Atractylodes macrocephala* Koidz. [Asteraceae, *Atractylodis Macrocephalae Rhizoma*] 10g, *Cynomorium coccineum* subsp. *songaricum* (Rupr.) J.Léonard [Cynomoriaceae, *Cynomorii Herba*] 10g, *Acorus verus* (L.) Raf. [Acoraceae, *Acori Tatarinowii Rhizoma*] 10g, *Epimedium brevicornu* Maxim. [Berberidaceae, *Epimedii Folium*] 10g, *Cuscuta australis* R.Br. [Convolvulaceae, *Cuscutae Semen*] 10g, *Conioselinum anthriscoides* (H.Boissieu) Pimenov & Kljuykov [Apiaceae, *Ligustici Rhizoma et Radix*] 10g, *Prunus persica* (L.) Batsch [Rosaceae, *Persicae Semen*] 10g, *Curcuma phaeocaulis* Valeton [Zingiberaceae, *Curcumae Rhizoma*] 10g, *Gleditsia sinensis* Lam. [Fabaceae, *Gleditsiae Spina*] 10g, *Pinellia ternata* (Thunb.) Makino [Araceae, *Pinelliae Rhizoma Praeparatum*] 10g, *Citrus reticulata* Blanco [Rutaceae, *Citri Reticulatae Pericarpium*] 10g | Decoction |
| Duan, X (2020) | Shiying Yulin Decoction | Fluoritum (Calcium Fluoride, CaF₂) 30g, *Epimedium brevicornu* Maxim. [Berberidaceae, *Epimedii Folium*] 30g, *Cuscuta australis* R.Br. [Convolvulaceae, *Cuscutae Semen*] 9g, *Dipsacus asper* Wall. ex DC. [Caprifoliaceae, *Dipsaci Radix*] 15g, *Neolitsea cassia* (L.) Kosterm. [Lauraceae, *Cinnamomi Cortex*] 9g, *Lycium barbarum* L. [Solanaceae, *Lycii Fructus*] 9g, *Angelica sinensis* (Oliv.) Diels [Apiaceae, *Angelicae Sinensis Radix*] 15g, *Paeonia lactiflora* Pall. [Ranunculaceae, *Paeoniae Radix Alba*] 9g, *Paeonia lactiflora* Pall. [Ranunculaceae, *Paeoniae Radix Rubra*] 9g, *Conioselinum anthriscoides* (H.Boissieu) Pimenov & Kljuykov [Apiaceae, *Ligustici Rhizoma et Radix*] 12g, *Cyperus rotundus* L. [Cyperaceae, *Cyperi Rhizoma*] 9g, *Cyathula officinalis* K.C.Kuan [Amaranthaceae, *Cyathulae Radix*] 15g, *Eucommia ulmoides* Oliv. [Eucommiaceae, *Eucommiae Cortex*] 15g, *Glycyrrhiza uralensis* Fisch. [Fabaceae, *Glycyrrhizae Radix et Rhizoma*] 9g | Decoction |
| Fu, R (2020) | Jianpi Huatan Decoction | *Atractylodes lancea* (Thunb.) DC. [Asteraceae, *Atractylodis Rhizoma*] 10g, *Cyperus rotundus* L. [Cyperaceae, *Cyperi Rhizoma*] 10g, *Citrus reticulata* Blanco [Rutaceae, *Citri Reticulatae Pericarpium*] 10g, *Pinellia ternata* (Thunb.) Makino [Araceae, *Pinelliae Rhizoma*] 10g, *Poria cocos* (Schw.) Wolf [Polyporaceae, *Poria*] 10g, *Codonopsis pilosula* (Franch.) Nannf. [Campanulaceae, *Codonopsis Radix*] 15g, *Salvia miltiorrhiza* Bunge [Lamiaceae, *Salviae Miltiorrhizae Radix et Rhizoma*] 15g, *Angelica sinensis* (Oliv.) Diels [Apiaceae, *Angelicae Sinensis Radix*] 10g, *Paeonia lactiflora* Pall. [Ranunculaceae, *Paeoniae Radix Rubra*] 15g, *Conioselinum anthriscoides* (H.Boissieu) Pimenov & Kljuykov [Apiaceae, *Ligustici Rhizoma et Radix*] 6g, *Rehmannia glutinosa* (Gaertn.) Libosch. ex DC. [Orobanchaceae, *Rehmanniae Radix*] 10g, *Curculigo orchioides* Gaertn. [Hypoxidaceae, *Curculiginis Rhizoma*] 10g, *Epimedium brevicornu* Maxim. [Berberidaceae, *Epimedii Folium*] 10g, *Crataegus pinnatifida* Bunge [Rosaceae, *Crataegi Fructus*] 10g, *Gallus gallus* subsp. *domesticus* (Linnaeus, 1758) [Phasianidae, *Endothelium Corneum Gigeriae Galli*] 10g | Granula |
| He, J (2020) | Jianpi Bushen Decoction | *Astragalus membranaceus* (Fisch.) Bge. [Fabaceae, *Astragali Radix*] 20g, *Cistanche deserticola* Ma [Orobanchaceae, *Cistanches Herba*] 30g, *Paeonia lactiflora* Pall. [Ranunculaceae, *Paeoniae Radix Rubra*] 20g, *Poria cocos* (Schw.) Wolf [Polyporaceae, *Poria*] 15g, *Angelica sinensis* (Oliv.) Diels [Apiaceae, *Angelicae Sinensis Radix*] 20g, *Cuscuta australis* R.Br. [Convolvulaceae, *Cuscutae Semen*] 15g, *Epimedium brevicornu* Maxim. [Berberidaceae, *Epimedii Folium*] 15g | Decoction |
| Wang, S (2020) | Bushen Quyu Huatan Decoction | *Epimedium brevicornu* Maxim. [Berberidaceae, *Epimedii Folium*] 10g, *Cuscuta australis*R.Br. [Convolvulaceae, *Cuscutae Semen*] 10g, *Cornus officinalis* Siebold & Zucc. [Cornaceae, *Corni Fructus*] 10g, *Lycium barbarum* L. [Solanaceae, *Lycii Fructus*] 10g, *Dioscorea oppositifolia* L. [Dioscoreaceae, *Dioscoreae Rhizoma*] 10g, *Paeonia lactiflora* Pall. [Ranunculaceae, *Paeoniae Radix Rubra*] 10g, *Paeonia lactiflora* Pall. [Ranunculaceae, *Paeoniae Radix Alba*] 10g, *Rehmannia glutinosa*(Gaertn.) Libosch. ex DC. [Orobanchaceae, *Rehmanniae Radix*] 15g, *Prunus persica* (L.) Batsch [Rosaceae, *Persicae Semen*] 10g, *Carthamus tinctorius* L. [Asteraceae, *Carthami Flos*] 10g, *Angelica sinensis* (Oliv.) Diels [Apiaceae, *Angelicae Sinensis Radix*] 10g, *Poria cocos* (Schw.) Wolf [Polyporaceae, *Poria*] 10g, *Paeonia × suffruticosa* Andrews [Paeoniaceae, *Moutan Cortex*] 10g, *Gleditsia sinensis* Lam. [Fabaceae, *Gleditsiae Spina*] 10g, *Atractylodes lancea* (Thunb.) DC. [Asteraceae, *Atractylodis Rhizoma*] 12g, *Citrus reticulata* Blanco [Rutaceae, *Citri Reticulatae Pericarpium*] 6g | Other |
| Lin, H (2020) | Cangfu Daotan Decoction | *Prunella vulgaris* L. [Lamiaceae, *Prunellae Spica*] 6g, *Citrus × aurantium* L. [Rutaceae, *Aurantii Fructus*] 6g, *Arisaema erubescens* (Wall.) Schott [Araceae, *Arisaematis Rhizoma*] 6g, Massa Medicata Fermentata 6g, *Angelica sinensis* (Oliv.) Diels [Apiaceae, *Angelicae Sinensis Radix*] 6g, *Pinellia ternata* (Thunb.) Makino [Araceae, *Pinelliae Rhizoma*] 6g, *Glycyrrhiza uralensis* Fisch. [Fabaceae, *Glycyrrhizae Radix et Rhizoma*] 6g, *Conioselinum anthriscoides* (H.Boissieu) Pimenov & Kljuykov [Apiaceae, *Ligustici Rhizoma et Radix*] 6g, *Cyperus rotundus* L. [Cyperaceae, *Cyperi Rhizoma*] 15g, *Atractylodes lancea*(Thunb.) DC. [Asteraceae, *Atractylodis Rhizoma*] 15g, *Poria cocos* (Schw.) Wolf [Polyporaceae, *Poria*] 10g, *Citrus reticulata* Blanco [Rutaceae, *Citri Reticulatae Pericarpium*] 10g, *Prunus persica* (L.) Batsch [Rosaceae, *Persicae Semen*] 10g, *Zingiber officinale* Roscoe [Zingiberaceae, *Zingiberis Rhizoma Recens*] 3g | Decoction |
| Hou, X (2020) | Bushen Qutan Decoction | *Epimedium brevicornu* Maxim. [Berberidaceae, *Epimedii Folium*] 15g, Fluoritum (Calcium Fluoride, CaF₂) 20g, *Gynochthodes officinalis* (F.C.How) Razafim. & B.Bremer [Rubiaceae, *Morindae Officinalis Radix*] 15g, *Cervus nippon* Temminck, 1838 [Cervidae, *Cervi Cornu Praeparatum*] 12g, *Cuscuta australis* R.Br. [Convolvulaceae, *Cuscutae Semen*] 15g, *Atractylodes lancea* (Thunb.) DC. [Asteraceae, *Atractylodis Rhizoma*] 15g, *Poria cocos* (Schw.) Wolf [Polyporaceae, *Poria*] 15g, *Citrus reticulata* Blanco [Rutaceae, *Citri Reticulatae Pericarpium*] 12g, *Pinellia ternata* (Thunb.) Makino [Araceae, *Pinelliae Rhizoma Praeparatum cum Alumine*] 12g, *Gleditsia sinensis* Lam. [Fabaceae, *Gleditsiae Spina*] 12g, *Cyathula officinalis* K.C.Kuan [Amaranthaceae, *Cyathulae Radix*] 15g, *Angelica sinensis* (Oliv.) Diels [Apiaceae, *Angelicae Sinensis Radix*] 12g, *Cyperus rotundus* L. [Cyperaceae, *Cyperi Rhizoma*] 15g, *Crataegus pinnatifida* Bunge [Rosaceae, *Crataegi Fructus*] 20g, *Zanthoxylum bungeanum* Maxim. [Rutaceae, *Zanthoxyli Pericarpium*] 3g, *Glycyrrhiza uralensis* Fisch. [Fabaceae, *Glycyrrhizae Radix et Rhizoma*] 6g | Decoction |
| Shi, Q (2020) | Bushen Huatan Decoction | *Rehmannia glutinosa* (Gaertn.) Libosch. ex DC. [Orobanchaceae, *Rehmanniae Radix*] 15g, *Conioselinum anthriscoides* (H.Boissieu) Pimenov & Kljuykov [Apiaceae, *Ligustici Rhizoma et Radix*] 15g, *Epimedium brevicornu* Maxim. [Berberidaceae, *Epimedii Folium*] 15g, *Angelica sinensis* (Oliv.) Diels [Apiaceae, *Angelicae Sinensis Radix*] 15g, *Cyperus rotundus* L. [Cyperaceae, *Cyperi Rhizoma*] 10g, *Poria cocos* (Schw.) Wolf [Polyporaceae, *Poria*] 10g, *Cuscuta australis* R.Br. [Convolvulaceae, *Cuscutae Semen*] 10g, *Pinellia ternata* (Thunb.) Makino [Araceae, *Pinelliae Rhizoma*] 8g, *Lycium barbarum* L. [Solanaceae, *Lycii Fructus*] 8g, *Atractylodes macrocephala* Koidz. [Asteraceae, *Atractylodis Macrocephalae Rhizoma*] 8g, *Salvia miltiorrhiza* Bunge [Lamiaceae, *Salviae Miltiorrhizae Radix et Rhizoma*] 6g, *Dioscorea oppositifolia* L. [Dioscoreaceae, *Dioscoreae Rhizoma*] 6g | Decoction |
| Li, Y (2020) | Self-formulated Prescription | *Rehmannia glutinosa* (Gaertn.) Libosch. ex DC. [Orobanchaceae, *Rehmanniae Radix*] 20g, *Codonopsis pilosula* (Franch.) Nannf. [Campanulaceae, *Codonopsis Radix*] 20g, *Lycium barbarum* L. [Solanaceae, *Lycii Fructus*] 15g, *Epimedium brevicornu* Maxim. [Berberidaceae, *Epimedii Folium*] 15g, *Cistanche deserticola* Ma [Orobanchaceae, *Cistanches Herba*] 15g, *Angelica sinensis* (Oliv.) Diels [Apiaceae, *Angelicae Sinensis Radix*] 15g, *Atractylodes macrocephala* Koidz. [Asteraceae, *Atractylodis Macrocephalae Rhizoma*] 15g, *Poria cocos* (Schw.) Wolf [Polyporaceae, *Poria*] 15g, *Sinapis alba* L. [Brassicaceae, *Sinapis Semen*] 10g, *Citrus reticulata* Blanco [Rutaceae, *Citri Reticulatae Pericarpium*] 10g, *Cervus nippon* Temminck, 1838 [Cervidae, *Cervi Cornu Praeparatum*] 10g, *Citrus reticulata* Blanco [Rutaceae, *Citri Reticulatae Pericarpium Viride*] 5g, *Glycyrrhiza uralensis* Fisch. [Fabaceae, *Glycyrrhizae Radix et Rhizoma*] 6g | Decoction |
| Deng, X (2019) | Danggui Dihuang Decoction combined with Taoren Siwu Decoction | *Angelica sinensis* (Oliv.) Diels [Apiaceae, *Angelicae Sinensis Radix*] 15g, *Rehmannia glutinosa* (Gaertn.) Libosch. ex DC. [Orobanchaceae, *Rehmanniae Radix*] 15g, *Cornus officinalis* Siebold & Zucc. [Cornaceae, *Corni Fructus*] 10g, *Dioscorea oppositifolia* L. [Dioscoreaceae, *Dioscoreae Rhizoma*] 15g, *Eucommia ulmoides* Oliv. [Eucommiaceae, *Eucommiae Cortex*] 15g, *Achyranthes bidentata* Blume [Amaranthaceae, *Achyranthis Bidentatae Radix*] 10g, *Cuscuta australis* R.Br. [Convolvulaceae, *Cuscutae Semen*] 15g, P*runus persica* (L.) Batsch [Rosaceae, *Persicae Semen*] 15g, *Carthamus tinctorius* L. [Asteraceae, *Carthami Flos*] 5g, *Conioselinum anthriscoides* (H.Boissieu) Pimenov & Kljuykov [Apiaceae, *Ligustici Rhizoma et Radix*] 5g, *Paeonia lactiflora* Pall. [Ranunculaceae, *Paeoniae Radix Alba*] 10g, *Citrus reticulata* Blanco [Rutaceae, *Citri Reticulatae Pericarpium*] 5g, *Pinellia ternata* (Thunb.) Makino [Araceae, *Pinelliae Rhizoma Praeparatum cum Zingibere et Alumine*] 5g, *Glycyrrhiza uralensis* Fisch. [Fabaceae, *Glycyrrhizae Radix et Rhizoma*] 5g | Granula |
| Li, H (2019a) | Yishen Huatan Decoction | *Cuscuta australis* R.Br. [Convolvulaceae, *Cuscutae Semen*] 20g, *Epimedium brevicornu*Maxim. [Berberidaceae, *Epimedii Folium*] 15g, *Astragalus membranaceus* (Fisch.) Bge. [Fabaceae, *Astragali Radix*] 15g, *Rehmannia glutinosa* (Gaertn.) Libosch. ex DC. [Scrophulariaceae, *Rehmanniae Radix*] 12g, *Rehmannia glutinosa* (Gaertn.) Libosch. ex DC. [Orobanchaceae, *Rehmanniae Radix*] 12g, *Angelica sinensis* (Oliv.) Diels [Apiaceae, *Angelicae Sinensis Radix*] 12g, *Atractylodes lancea* (Thunb.) DC. [Asteraceae, *Atractylodis Rhizoma*] 12g, *Atractylodes macrocephala* Koidz. [Asteraceae, *Atractylodis Macrocephalae Rhizoma*] 12g, *Citrus reticulata* Blanco [Rutaceae, *Citri Reticulatae Pericarpium*] 12g, *Acorus verus* (L.) Raf. [Acoraceae, *Acori Tatarinowii Rhizoma*] 12g, *Pinellia ternata* (Thunb.) Makino [Araceae, *Pinelliae Rhizoma*] 12g | Decoction |
| Chen, Y (2019b) | Self-formulated Prescription | Fluoritum (Calcium Fluoride, CaF₂) 30g, *Epimedium brevicornu* Maxim. [Berberidaceae, *Epimedii Folium*] 20g, *Cervus nippon* Temminck, 1838 [Cervidae, *Cervi Cornu Praeparatum*] 20g, *Rehmannia glutinosa* (Gaertn.) Libosch. ex DC. [Orobanchaceae, *Rehmanniae Radix*] 12g, *Pinellia ternata* (Thunb.) Makino [Araceae, *Pinelliae Rhizoma Praeparatum cum Alumine*] 9g, *Citrus reticulata* Blanco [Rutaceae, *Citri Exocarpium Rubrum*] 12g, *Gleditsia sinensis* Lam. [Fabaceae, *Gleditsiae Spina*] 10g, *Atractylodes macrocephala* Koidz. [Asteraceae, *Atractylodis Macrocephalae Rhizoma*] 20g, *Poria cocos* (Schw.) Wolf [Polyporaceae, *Poria*] 20g, *Angelica sinensis* (Oliv.) Diels [Apiaceae, *Angelicae Sinensis Radix*] 12g, *Conioselinum anthriscoides* (H.Boissieu) Pimenov & Kljuykov [Apiaceae, *Ligustici Rhizoma et Radix*] 15g, *Paeonia lactiflora* Pall. [Ranunculaceae, *Paeoniae Radix Rubra*] 20g, *Cyperus rotundus* L. [Cyperaceae, *Cyperi Rhizoma*] 12g, *Crataegus pinnatifida* Bunge [Rosaceae, *Crataegi Fructus*] 10g, *Zanthoxylum bungeanum* Maxim. [Rutaceae, *Zanthoxyli Pericarpium*] 1.5g | Granula |
| Yang, Y (2019) | Xiaozhi Decoction | *Epimedium brevicornu* Maxim. [Berberidaceae, *Epimedii Folium*] 15g, *Pinellia ternata* (Thunb.) Makino [Araceae, *Pinelliae Rhizoma Praeparatum cum Zingibere et Alumine*] 9g, *Bupleurum chinense* DC. [Apiaceae, *Bupleuri Radix*] 9g, *Scutellaria baicalensis* Georgi [Lamiaceae, *Scutellariae Radix*] 9g, *Paeonia lactiflora* Pall. [Ranunculaceae, *Paeoniae Radix Alba*] 9g, *Rheum palmatum* L. [Polygonaceae, *Rhei Radix et Rhizoma Praeparata*] 6g, *Citrus × aurantium* L. [Rutaceae, *Aurantii Fructus*] 15g, *Conioselinum anthriscoides* (H.Boissieu) Pimenov & Kljuykov [Apiaceae, *Ligustici Rhizoma et Radix*] 9g, *Sargassum fusiforme* (Harvey) Setchell [Sargassaceae, *Sargassi Thallus*] 15g, *Senna obtusifolia* (L.) H.S.Irwin & Barneby [Fabaceae, *Cassiae Semen*] 15g, *Taxillus chinensis* (DC.) Danser [Loranthaceae, *Taxilli Herba*] 15g, *Gynostemma pentaphyllum* (Thunb.) Makino [Cucurbitaceae, *Gynostemmatis Herba*] 15g | Decoction |
| Di, X (2019) | Cangfu Daotan Decoction | *Atractylodes lancea* (Thunb.) DC. [Asteraceae, *Atractylodis Rhizoma*] 12g, *Cyperus rotundus* L. [Cyperaceae, *Cyperi Rhizoma*] 10g, *Citrus reticulata* Blanco [Rutaceae, *Citri Reticulatae Pericarpium*] 12g, *Pinellia ternata* (Thunb.) Makino [Araceae, *Pinelliae Rhizoma*] 12g, *Arisaema erubescens* (Wall.) Schott [Araceae, *Arisaema cum Bile*] 6g, *Poria cocos* (Schw.) Wolf [Polyporaceae, *Poria*] 15g, *Citrus × aurantium* L. [Rutaceae, *Aurantii Fructus*] 10g, Massa Medicata Fermentata 10g, *Zingiber officinale* Roscoe [Zingiberaceae, *Zingiberis Rhizoma Recens*] 6g, *Glycyrrhiza uralensis* Fisch. [Fabaceae, *Glycyrrhizae Radix et Rhizoma*] 6g | Decoction |
| Huang, C (2019) | Modified Cangfu Daotan Decoction | *Atractylodes lancea* (Thunb.) DC. [Asteraceae, *Atractylodis Rhizoma*] 10g, *Arisaema erubescens* (Wall.) Schott [Araceae, *Arisaema cum Bile*] 5g, *Pinellia ternata* (Thunb.) Makino [Araceae, *Pinelliae Rhizoma Praeparatum*] 10g, *Citrus × aurantium* L. [Rutaceae, *Aurantii Fructus*] 6g, *Cyperus rotundus*L. [Cyperaceae, *Cyperi Rhizoma*] 10g, *Citrus reticulata* Blanco [Rutaceae, *Citri Reticulatae Pericarpium*] 10g, *Poria cocos* (Schw.) Wolf [Polyporaceae, *Poria*] 15g, *Glycyrrhiza uralensis* Fisch. [Fabaceae, *Glycyrrhizae Radix et Rhizoma*] 10g, *Zingiber officinale* Roscoe [Zingiberaceae, *Zingiberis Rhizoma Recens*] 6g, *Gleditsia sinensis* Lam. [Fabaceae, *Gleditsiae Spina*] 10g, *Taxillus chinensis* (DC.) Danser [Loranthaceae, *Taxilli Herba*] 10g, *Fritillaria thunbergii* Miq. [Liliaceae, *Fritillariae Thunbergii Bulbus*] 10g, *Cornus officinalis* Siebold & Zucc. [Cornaceae, *Corni Fructus*] 10g, *Epimedium brevicornu* Maxim. [Berberidaceae, *Epimedii Folium*] 10g, *Cuscuta australis* R.Br. [Convolvulaceae, *Cuscutae Semen*] 10g | Decoction |
| Chen, Y (2019a) | Cangfu Daotan Decoction | *Atractylodes lancea* (Thunb.) DC. [Asteraceae, *Atractylodis Rhizoma*] 20g, *Cyperus rotundus* L. [Cyperaceae, *Cyperi Rhizoma*] 20g, *Poria cocos* (Schw.) Wolf [Polyporaceae, *Poria*] 20g, *Rehmannia glutinosa* (Gaertn.) Libosch. ex DC. [Orobanchaceae, *Rehmanniae Radix*] 20g, *Cervus nippon* Temminck, 1838 [Cervidae, *Colla Cornus Cervi*] 20g, *Citrus × aurantium* L. [Rutaceae, *Aurantii Fructus*] 10g, *Citrus reticulata* Blanco [Rutaceae, *Citri Reticulatae Pericarpium*] 10g, *Conioselinum anthriscoides* (H.Boissieu) Pimenov & Kljuykov [Apiaceae, *Ligustici Rhizoma et Radix*] 10g, *Pinellia ternata* (Thunb.) Makino [Araceae, *Pinelliae Rhizoma Praeparatum*] 10g, *Arisaema erubescens* (Wall.) Schott [Araceae, *Arisaema cum Bile*] 10g, *Glycyrrhiza uralensis* Fisch. [Fabaceae, *Glycyrrhizae Radix et Rhizoma*] 3g | Decoction |
| Zhang, Q (2019) | Modified Cangfu Daotan Decoction | *Atractylodes lancea* (Thunb.) DC. [Asteraceae, *Atractylodis Rhizoma*] 6g, *Cyperus rotundus* L. [Cyperaceae, *Cyperi Rhizoma*] 10g, *Citrus reticulata* Blanco [Rutaceae, *Citri Reticulatae Pericarpium*] 10g, *Pinellia ternata* (Thunb.) Makino [Araceae, *Pinelliae Rhizoma Praeparatum cum Zingibere et Alumine*] 12g, *Poria cocos* (Schw.) Wolf [Polyporaceae, *Poria*] 12g, *Citrus × aurantium* L. [Rutaceae, *Aurantii Fructus*] 15g, *Bambusa tuldoides* Munro [Poaceae, *Bambusae Caulis in Taenias*] 15g, *Angelica sinensis* (Oliv.) Diels [Apiaceae, A*ngelicae Sinensis Radix*] 10g, *Salvia miltiorrhiza* Bunge [Lamiaceae, *Salviae Miltiorrhizae Radix et Rhizoma*] 15g, *Cuscuta australis*R.Br. [Convolvulaceae, *Cuscutae Semen*] 15g, *Cistanche deserticola* Ma [Orobanchaceae, *Cistanches Herba*] 15g, *Epimedium brevicornu* Maxim. [Berberidaceae, *Epimedii Folium*] 15g, *Reynoutria japonica* Houtt. [Polygonaceae, *Polygoni Cuspidati Rhizoma et Radix*] 20g, *Coptis chinensis* Franch. [Ranunculaceae, *Coptidis Rhizoma*] 3g | Decoction |
| Bai, R (2018) | Qihuang Zengmin Decoction | *Astragalus membranaceus* (Fisch.) Bge. [Fabaceae, *Astragali Radix*] 30g, *Rehmannia glutinosa* (Gaertn.) Libosch. ex DC. [Orobanchaceae, *Rehmanniae Radix*] 30g, *Polygonatum cyrtonema* Hua [Asparagaceae, *Polygonati Rhizoma*] 30g, *Coptis chinensis* Franch. [Ranunculaceae, *Coptidis Rhizoma*] 6g, P*anax notoginseng* (Burkill) F.H.Chen [Araliaceae, *Notoginseng Radix et Rhizoma*] 12g, *Atractylodes lancea*(Thunb.) DC. [Asteraceae, *Atractylodis Rhizoma*] 15g, *Gynostemma pentaphyllum* (Thunb.) Makino [Cucurbitaceae, *Gynostemmatis Herba*] 15g | Granula |
| Zhang, H (2018) | Modified Cangfu Daotan Decoction | *Atractylodes lancea* (Thunb.) DC. [Asteraceae, *Atractylodis Rhizoma*] 10g, *Cyperus rotundus* L. [Cyperaceae, *Cyperi Rhizoma*] 15g, *Arisaema erubescens* (Wall.) Schott [Araceae, *Arisaema cum Bile*] 10g, *Acorus verus* (L.) Raf. [Acoraceae, *Acori Tatarinowii Rhizoma*] 10g, *Citrus reticulata* Blanco [Rutaceae, *Citri Reticulatae Pericarpium*] 6g, *Pinellia ternata* (Thunb.) Makino [Araceae, *Pinelliae Rhizoma Praeparatum*] 10g, *Salvia miltiorrhiza* Bunge [Lamiaceae, *Salviae Miltiorrhizae Radix et Rhizoma*] 15g, *Cuscuta australis* R.Br. [Convolvulaceae, *Cuscutae Semen*] 20g, *Poria cocos* (Schw.) Wolf [Polyporaceae, *Poria*] 15g, *Rehmannia glutinosa* (Gaertn.) Libosch. ex DC. [Orobanchaceae, *Rehmanniae Radix*] 15g, *Taxillus chinensis* (DC.) Danser [Loranthaceae, *Taxilli Herba*] 15g, *Crataegus pinnatifida* Bunge [Rosaceae, *Crataegi Fructus*] 15g, *Zingiber officinale* Roscoe [Zingiberaceae, *Zingiberis Rhizoma Recens*] 6g, *Glycyrrhiza uralensis* Fisch. [Fabaceae, *Glycyrrhizae Radix et Rhizoma*] 6g | Decoction |
| Yang, Z (2018) | Modified Guizhi Fuling Pill Combined with Danggui Shaoyao Decoction | *Neolitsea cassia* (L.) Kosterm. [Lauraceae, *Cinnamomi Cortex*] 30g, *Poria cocos* (Schw.) Wolf [Polyporaceae, *Poria*] 60g, *Paeonia lactiflora* Pall. [Ranunculaceae, *Paeoniae Radix Rubra*] 15g, *Prunus persica* (L.) Batsch [Rosaceae, *Persicae Semen*] 10g, *Paeonia × suffruticosa* Andrews [Paeoniaceae, *Moutan Cortex*] 10g, *Angelica sinensis* (Oliv.) Diels [Apiaceae, *Angelicae Sinensis Radix*] 15g, *Conioselinum anthriscoides* (H.Boissieu) Pimenov & Kljuykov [Apiaceae, *Ligustici Rhizoma et Radix*] 10g, *Atractylodes macrocephala* Koidz. [Asteraceae, *Atractylodis Macrocephalae Rhizoma*] 30g, *Alisma plantago-aquatica* subsp. *orientale* (Sam.) Sam. [Alismataceae, *Alismatis Rhizoma*] 15g | Granula |
| Xie, P (2018) | Bushen Shugan Huayu Qutan Decoction | *Cistanche deserticola* Ma [Orobanchaceae, *Cistanches Herba*] 20g, *Ligustrum lucidum* W.T.Aiton [Oleaceae, *Ligustri Lucidi Fructus*] 15g, *Cuscuta australis* R.Br. [Convolvulaceae, *Cuscutae Semen*] 15g, *Lycium barbarum* L. [Solanaceae, *Lycii Fructus*] 10g, *Rehmannia glutinosa* (Gaertn.) Libosch. ex DC. [Orobanchaceae, *Rehmanniae Radix*] 10g, *Bupleurum chinense* DC. [Apiaceae, *Bupleuri Radix*] 10g, *Paeonia lactiflora* Pall. [Ranunculaceae, *Paeoniae Radix Alba*] 10g, *Salvia miltiorrhiza* Bunge [Lamiaceae, *Salviae Miltiorrhizae Radix et Rhizoma*] 10g, *Conioselinum anthriscoides* (H.Boissieu) Pimenov & Kljuykov [Apiaceae, *Ligustici Rhizoma et Radix*] 10g, *Lycopus lucidus* Turcz. ex Benth. [Lamiaceae, *Lycopi Herba*] 15g, *Poria cocos* (Schw.) Wolf [Polyporaceae, *Poria*] 15g, *Pinellia ternata* (Thunb.) Makino [Araceae, *Pinelliae Rhizoma*] 15g, *Acorus verus* (L.) Raf. [Acoraceae, *Acori Tatarinowii Rhizoma*] 10g, *Crataegus pinnatifida* Bunge [Rosaceae, *Crataegi Fructus*] 15g | Decoction |
| Liu, Y (2018) | Self-formulated Prescription | *Astragalus membranaceus* (Fisch.) Bge. [Fabaceae, *Astragali Radix*] 20g, *Paeonia lactiflora* Pall. [Ranunculaceae, *Paeoniae Radix Rubra*] 20g, *Angelica sinensis* (Oliv.) Diels [Apiaceae, *Angelicae Sinensis Radix*] 20g, *Poria cocos* (Schw.) Wolf [Polyporaceae, *Poria*] 15g, *Cuscuta australis* R.Br. [Convolvulaceae, *Cuscutae Semen*] 15g, *Epimedium brevicornu* Maxim. [Berberidaceae, *Epimedii Folium*] 15g, *Cistanche deserticola* Ma [Orobanchaceae, *Cistanches Herba*] 30g | Decoction |
| Xu, J (2017) | Tanzhixiao Granula | *Atractylodes lancea* (Thunb.) DC. [Asteraceae, *Atractylodis Rhizoma*], *Poria cocos* (Schw.) Wolf [Polyporaceae, *Poria*], *Alisma plantago-aquatica* subsp. *orientale* (Sam.) Sam. [Alismataceae, *Alismatis Rhizoma*], *Cuscuta australis* R.Br. [Convolvulaceae, *Cuscutae Semen*], *Epimedium brevicornu*Maxim. [Berberidaceae, *Epimedii Folium*] | Granula |
| Fu, Y (2017) | Modified Cangfu Daotan Decoction | *Atractylodes lancea* (Thunb.) DC. [Asteraceae, *Atractylodis Rhizoma*] 10g, *Cyperus rotundus* L. [Cyperaceae, *Cyperi Rhizoma*] 10g, *Pinellia ternata* (Thunb.) Makino [Araceae, *Pinelliae Rhizoma Praeparatum*] 10g, *Citrus reticulata* Blanco [Rutaceae, *Citri Reticulatae Pericarpium*] 6g, *Acorus verus* (L.) Raf. [Acoraceae, *Acori Tatarinowii Rhizoma*] 10g, *Poria cocos* (Schw.) Wolf [Polyporaceae, *Poria*] 30g, *Astragalus membranaceus* (Fisch.) Bge. [Fabaceae, *Astragali Radix*] 30g, *Gleditsia sinensis* Lam. [Fabaceae, *Gleditsiae Spina*] 10g, *Epimedium brevicornu* Maxim. [Berberidaceae, *Epimedii Folium*] 15g, *Angelica sinensis* (Oliv.) Diels [Apiaceae, *Angelicae Sinensis Radix*] 10g, *Salvia miltiorrhiza* Bunge [Lamiaceae, *Salviae Miltiorrhizae Radix et Rhizoma*] 15g | Decoction |
| Zhou, D (2017) | Heqi Powder | *Taxillus chinensis* (DC.) Danser [Loranthaceae, *Taxilli Herba*], *Astragalus membranaceus* (Fisch.) Bge. [Fabaceae, *Astragali Radix*], *Dioscorea oppositifolia* L. [Dioscoreaceae, *Dioscoreae Rhizoma*], *Prunus persica* (L.) Batsch [Rosaceae, *Persicae Semen*], *Senna obtusifolia* (L.) H.S.Irwin & Barneby [Fabaceae, *Cassiae Semen*], *Acorus verus* (L.) Raf. [Acoraceae, *Acori Tatarinowii Rhizoma*], *Polygonum multiflorum* Thunb. [Polygonaceae, *Polygoni Multiflori Radix Praeparata*], *Pinellia ternata* (Thunb.) Makino [Araceae, *Pinelliae Rhizoma*], *Carthamus tinctorius* L. [Asteraceae, *Carthami Flos*], *Angelica sinensis* (Oliv.) Diels [Apiaceae, *Angelicae Sinensis Radix*], *Benincasa hispida* (Thunb.) Cogn. [Cucurbitaceae, *Benincasae Exocarpium*], *Curculigo orchioides* Gaertn. [Hypoxidaceae, *Curculiginis Rhizoma*], *Epimedium brevicornu* Maxim. [Berberidaceae, *Epimedii Folium*], *Cuscuta australis* R.Br. [Convolvulaceae, *Cuscutae Semen*] | Granula |
| Liu, M (2017) | Bushen Huatan Decoction | Fluoritum (Calcium Fluoride, CaF₂) 15g, *Epimedium brevicornu* Maxim. [Berberidaceae, Epimedii Folium] 10g, *Citrus reticulata* Blanco [Rutaceae, *Citri Reticulatae Pericarpium*] 10g, *Pinellia ternata* (Thunb.) Makino [Araceae, *Pinelliae Rhizoma Praeparatum*] 10g, *Poria cocos* (Schw.) Wolf [Polyporaceae, *Poria*] 10g, *Atractylodes lancea* (Thunb.) DC. [Asteraceae, *Atractylodis Rhizoma*] 10g, *Cyathula officinalis* K.C.Kuan [Amaranthaceae, *Cyathulae Radix*] 10g, *Paeonia lactiflora* Pall. [Ranunculaceae, P*aeoniae Radix Rubra*] 10g, *Angelica sinensis* (Oliv.) Diels [Apiaceae, *Angelicae Sinensis Radix*] 10g, *Salvia miltiorrhiza* Bunge [Lamiaceae, *Salviae Miltiorrhizae Radix et Rhizoma*] 10g, *Conioselinum anthriscoides* (H.Boissieu) Pimenov & Kljuykov [Apiaceae, *Ligustici Rhizoma et Radix*] 10g, *Cyperus rotundus*L. [Cyperaceae, *Cyperi Rhizoma*] 10g, *Crataegus pinnatifida* Bunge [Rosaceae, *Crataegi Fructus*] 10g | Decoction |
| Ye, L (2017) | Sanhuang Decoction | *Coptis chinensis* Franch. [Ranunculaceae, *Coptidis Rhizoma*] 3g, *Scutellaria baicalensis* Georgi [Lamiaceae, *Scutellariae Radix*] 10g, *Rheum palmatum* L. [Polygonaceae, *Rhei Radix et Rhizoma Praeparata*] 6g | Granula |
| Guo, R (2017) | Cangfu Daotan Decoction | *Atractylodes lancea* (Thunb.) DC. [Asteraceae, *Atractylodis Rhizoma*] 12g, *Pinellia ternata* (Thunb.) Makino [Araceae, *Pinelliae Rhizoma*] 10g, *Citrus reticulata* Blanco [Rutaceae, *Citri Reticulatae Pericarpium*] 9g, *Cyperus rotundus* L. [Cyperaceae, *Cyperi Rhizoma*] 12g, *Astragalus membranaceus* (Fisch.) Bge. [Fabaceae, *Astragali Radix*] 30g, *Poria cocos* (Schw.) Wolf [Polyporaceae, *Poria*] 30g, *Epimedium brevicornu* Maxim. [Berberidaceae, *Epimedii Folium*] 15g, *Acorus verus* (L.) Raf. [Acoraceae, *Acori Tatarinowii Rhizoma*] 12g, *Angelica sinensis* (Oliv.) Diels [Apiaceae, *Angelicae Sinensis Radix*] 15g, *Gleditsia sinensis* Lam. [Fabaceae, *Gleditsiae Spina*] 12g, *Salvia miltiorrhiza* Bunge [Lamiaceae, *Salviae Miltiorrhizae Radix et Rhizoma*] 12g | Decoction |
| Lin, H (2017) | Huatan Tongmai Decoction | *Pinellia ternata* (Thunb.) Makino [Araceae, *Pinelliae Rhizoma Praeparatum*] 15g、*Atractylodes lancea* (Thunb.) DC. [Asteraceae, *Atractylodis Rhizoma*] 15g、*Atractylodes macrocephala* Koidz. [Asteraceae, *Atractylodis Macrocephalae Rhizoma*] 15g、*Poria cocos* (Schw.) Wolf [Polyporaceae, *Poria*] 20g、*Spatholobus suberectus* Dunn [Fabaceae, *Spatholobi Caulis*] 20g、*Angelica sinensis* (Oliv.) Diels [Apiaceae, *Angelicae Sinensis Radix*] 10g、*Salvia miltiorrhiza* Bunge [Lamiaceae, *Salviae Miltiorrhizae Radix et Rhizoma*] 15g、*Prunus persica* (L.) Batsch [Rosaceae, *Persicae Semen*] 10g、*Lycopus lucidus* Turcz. ex Benth. [Lamiaceae, *Lycopi Herba*] 15g、*Arca subcrenata* Lischke, 1869 [Arcidae, *Arcae Concha*] 10g、*Citrus reticulata* Blanco [Rutaceae, *Citri Reticulatae Pericarpium*] 6g、*Conioselinum anthriscoides* (H.Boissieu) Pimenov & Kljuykov [Apiaceae, *Ligustici Rhizoma et Radix*] 10g、*Cyperus rotundus* L. [Cyperaceae, *Cyperi Rhizoma*] 15g | Decoction |
| Song, C (2016) | Jianpi Lishi Yiqi Yangyin Decoction | *Atractylodes macrocephala* Koidz. [Asteraceae, *Atractylodis Macrocephalae Rhizoma*], P*inellia ternata* (Thunb.) Makino [Araceae, *Pinelliae Rhizoma*], *Citrus reticulata* Blanco [Rutaceae, *Citri Reticulatae Pericarpium*], *Poria cocos* (Schw.) Wolf [Polyporaceae, *Poria*], *Dioscorea oppositifolia* L. [Dioscoreaceae, *Dioscoreae Rhizoma*], *Cornus officinalis* Siebold & Zucc. [Cornaceae, *Corni Fructus*], *Coix lacryma-jobi* var. *ma-yuen* (Rom.Caill.) Stapf [Poaceae, *Coicis Semen*], *Senna obtusifolia* (L.) H.S.Irwin & Barneby [Fabaceae, *Cassiae Semen*], *Rehmannia glutinosa* (Gaertn.) Libosch. ex DC. [Orobanchaceae, *Rehmanniae Radix*], *Angelica sinensis* (Oliv.) Diels [Apiaceae, *Angelicae Sinensis Radix*], *Paeonia lactiflora* Pall. [Ranunculaceae, *Paeoniae Radix Alba*], *Astragalus membranaceus* (Fisch.) Bge. [Fabaceae, *Astragali Radix*], *Leonurus japonicus* Houtt. [Lamiaceae, *Leonuri Herba*], *Dendrobium nobile* Lindl. [Orchidaceae, *Dendrobii Caulis*], *Crataegus pinnatifida* Bunge [Rosaceae, *Crataegi Fructus*], *Pelodiscus sinensis* (Wiegmann, 1835) [Trionychidae, *Trionycis Carapax*], *Cuscuta australis* R.Br. [Convolvulaceae, *Cuscutae Semen*], *Glycyrrhiza uralensis* Fisch. [Fabaceae, *Glycyrrhizae Radix et Rhizoma*] | Granula |
| Huang, C (2016) | Buqi Huatan Xingqi Decoction | *Rehmannia glutinosa* (Gaertn.) Libosch. ex DC. [Orobanchaceae, *Rehmanniae Radix*] 15g, *Cornus officinalis* Siebold & Zucc. [Cornaceae, *Corni Fructus*] 15g, *Dioscorea oppositifolia* L. [Dioscoreaceae, *Dioscoreae Rhizoma*] 15g, *Cuscuta australis* R.Br. [Convolvulaceae, *Cuscutae Semen*], *Lycium barbarum* L. [Solanaceae, *Lycii Fructus*] 15g, *Eucommia ulmoides* Oliv. [Eucommiaceae, *Eucommiae Cortex*] 15g, *Atractylodes lancea* (Thunb.) DC. [Asteraceae, *Atractylodis Rhizoma*] 10g, *Pinellia ternata* (Thunb.) Makino [Araceae, *Pinelliae Rhizoma Praeparatum*] 10g, *Acorus verus* (L.) Raf. [Acoraceae, *Acori Tatarinowii Rhizoma*] 10g, *Poria cocos* (Schw.) Wolf [Polyporaceae, *Poria*] 15g, *Citrus reticulata* Blanco [Rutaceae, *Citri Reticulatae Pericarpium*] 10g, *Curcuma aromatica* Salisb. [Zingiberaceae, *Curcumae Rhizoma*] 15g, *Cyperus rotundus* L. [Cyperaceae, *Cyperi Rhizoma*] 10g, *Citrus × aurantium* L. [Rutaceae, *Aurantii Fructus*] 15g, *Salvia miltiorrhiza* Bunge [Lamiaceae, *Salviae Miltiorrhizae Radix et Rhizoma*] 10g, *Spatholobus suberectus* Dunn [Fabaceae, *Spatholobi Caulis*] 30g, *Glycyrrhiza uralensis* Fisch. [Fabaceae, *Glycyrrhizae Radix et Rhizoma*] 5g | Decoction |
| Huang, J (2016) | Self-formulated Prescription | *Taxillus chinensis* (DC.) Danser [Loranthaceae, *Taxilli Herba*] 10g, *Equus asinus* Linnaeus, 1758 [Equidae, *Colla Corii Asini*] 6g, *Cuscuta australis* R.Br. [Convolvulaceae, *Cuscutae Semen*] 10g, *Dipsacus asper* Wall. ex DC. [Caprifoliaceae, *Dipsaci Radix*] 10g, *Eucommia ulmoides* Oliv. [Eucommiaceae, *Eucommiae Cortex*] 10g, *Codonopsis pilosula*(Franch.) Nannf. [Campanulaceae, *Codonopsis Radix*] 10g, *Poria cocos* (Schw.) Wolf [Polyporaceae, *Poria*] 10g, *Dioscorea oppositifolia* L. [Dioscoreaceae, *Dioscoreae Rhizoma*] 10g, *Perilla frutescens* (L.) Britton [Lamiaceae, *Perillae Folium*] 10g, *Atractylodes macrocephala* Koidz. [Asteraceae, *Atractylodis Macrocephalae Rhizoma*] 10g, *Glycyrrhiza uralensis* Fisch. [Fabaceae, *Glycyrrhizae Radix et Rhizoma*] 6g | Granula |
| Song, Y (2015) | Yishen Huatan Decoction | *Astragalus membranaceus* (Fisch.) Bge. [Fabaceae, *Astragali Radix*] 15g、*Rehmannia glutinosa* (Gaertn.) Libosch. ex DC. [Scrophulariaceae, *Rehmanniae Radix*] 12g、*Rehmannia glutinosa* (Gaertn.) Libosch. ex DC. [Orobanchaceae, *Rehmanniae Radix*] 12g、*Angelica sinensis* (Oliv.) Diels [Apiaceae, *Angelicae Sinensis Radix*] 12g、*Atractylodes lancea* (Thunb.) DC. [Asteraceae, *Atractylodis Rhizoma*] 12g、*Atractylodes macrocephala* Koidz. [Asteraceae, *Atractylodis Macrocephalae Rhizoma*] 20g、*Citrus reticulata* Blanco [Rutaceae, *Citri Reticulatae Pericarpium*] 12g、*Pinellia ternata* (Thunb.) Makino [Araceae, *Pinelliae Rhizoma*] 12g、*Acorus verus* (L.) Raf. [Acoraceae, *Acori Tatarinowii Rhizoma*] 12g、*Cuscuta australis* R.Br. [Convolvulaceae, *Cuscutae Semen*] 20g、*Epimedium brevicornu* Maxim. [Berberidaceae, *Epimedii Folium*] 15g | Decoction |
| Wang, Q (2015) | Self-formulated Prescription | *Astragalus membranaceus* (Fisch.) Bge. [Fabaceae, *Astragali Radix*] 30g, *Atractylodes macrocephala* Koidz. [Asteraceae, *Atractylodis Macrocephalae Rhizoma*] 20g, *Atractylodes lancea* (Thunb.) DC. [Asteraceae, *Atractylodis Rhizoma*] 15g, *Taxillus chinensis*(DC.) Danser [Loranthaceae, *Taxilli Herba*] 20g, *Benincasa hispida* (Thunb.) Cogn. [Cucurbitaceae, *Benincasae Exocarpium*] 15g, *Crataegus pinnatifida* Bunge [Rosaceae, *Crataegi Fructus*] 30g, *Lycium barbarum* L. [Solanaceae, *Lycii Fructus*] 15g, *Dioscorea oppositifolia* L. [Dioscoreaceae, *Dioscoreae Rhizoma*] 15g, *Coix lacryma-jobi* var. *ma-yuen* (Rom.Caill.) Stapf [Poaceae, *Coicis Semen*] 15g, *Eupolyphaga sinensis* (Walker, 1868) [Corydiidae, *Eupolyphaga Corpus*] 12g, *Pinellia ternata* (Thunb.) Makino [Araceae, *Pinelliae Rhizoma Praeparatum*] 12g, *Acorus verus* (L.) Raf. [Acoraceae, *Acori Tatarinowii Rhizoma*] 12g | Decoction |
| Yin, Q (2015) | Bushen Huatan Decoction | *Salvia miltiorrhiza* Bunge [Lamiaceae, *Salviae Miltiorrhizae Radix et Rhizoma*] 30g、*Epimedium brevicornu* Maxim. [Berberidaceae, *Epimedii Folium*] 30g、*Astragalus membranaceus* (Fisch.) Bge. [Fabaceae, *Astragali Radix*] 50g、*Poria cocos* (Schw.) Wolf [Polyporaceae, *Poria*] 30g、*Atractylodes lancea* (Thunb.) DC. [Asteraceae, *Atractylodis Rhizoma*] 30g | Decoction |
| Lu, L (2013) | Chushi Huatan Decoction | *Poria cocos* (Schw.) Wolf [Polyporaceae, *Poria*], *Pinellia ternata* (Thunb.) Makino [Araceae, *Pinelliae Rhizoma*], *Citrus reticulata* Blanco [Rutaceae, *Citri Reticulatae Pericarpium*], *Atractylodes lancea* (Thunb.) DC. [Asteraceae, *Atractylodis Rhizoma*], *Cyperus rotundus* L. [Cyperaceae, *Cyperi Rhizoma*], *Arisaema erubescens* (Wall.) Schott [Araceae, *Arisaematis Rhizoma*], *Citrus × aurantium* L. [Rutaceae, *Aurantii Fructus*], *Zingiber officinale* Roscoe [Zingiberaceae, *Zingiberis Rhizoma Recens*], Massa Medicata Fermentata, *Prunus persica* (L.) Batsch [Rosaceae, *Persicae Semen*], *Angelica sinensis* (Oliv.) Diels [Apiaceae, *Angelicae Sinensis Radix*], *Conioselinum anthriscoides* (H.Boissieu) Pimenov & Kljuykov [Apiaceae, *Ligustici Rhizoma et Radix*], *Carthamus tinctorius* L. [Asteraceae, *Carthami Flos*], *Prunella vulgaris* L. [Lamiaceae, *Prunellae Spica*], *Glycyrrhiza uralensis* Fisch. [Fabaceae, *Glycyrrhizae Radix et Rhizoma*] | Granula |
| Jiao, N (2013) | Zaoshi Huatan Bushen Decoction | *Atractylodes lancea* (Thunb.) DC. [Asteraceae, *Atractylodis Rhizoma*] 12g, *Atractylodes macrocephala* Koidz. [Asteraceae, *Atractylodis Macrocephalae Rhizoma*] 20g, *Taxillus chinensis* (DC.) Danser [Loranthaceae, *Taxilli Herba*] 20g, *Crataegus pinnatifida* Bunge [Rosaceae, *Crataegi Fructus*] 30g, *Senna obtusifolia* (L.) H.S.Irwin & Barneby [Fabaceae, *Cassiae Semen*] 12g, *Acorus verus* (L.) Raf. [Acoraceae, *Acori Tatarinowii Rhizoma*] 12g, *Arisaema erubescens* (Wall.) Schott [Araceae, *Arisaema cum Bile*] 12g, *Sinapis alba* L. [Brassicaceae, *Sinapis Semen*] 12g, Fluoritum (Calcium Fluoride, CaF₂) 30g, *Homo sapiens* Linnaeus, 1758 [Hominidae, *Placentae Hominis Colla*] 12g, *Pinellia ternata* (Thunb.) Makino [Araceae, *Pinelliae Rhizoma Praeparatum*] 12g, *Alisma plantago-aquatica* subsp. *orientale* (Sam.) Sam. [Alismataceae, *Alismatis Rhizoma*] 12g | Granula |
| Feng, C (2009) | Modified Erchen Decoction | *Citrus reticulata* Blanco [Rutaceae, *Citri Reticulatae Pericarpium*] 10g、*Pinellia ternata* (Thunb.) Makino [Araceae, *Pinelliae Rhizoma*] 10g、*Poria cocos* (Schw.) Wolf [Polyporaceae, *Poria*] 15g、*Glycyrrhiza uralensis* Fisch. [Fabaceae, *Glycyrrhizae Radix et Rhizoma*] 5g、*Alisma plantago-aquatica* subsp. *orientale* (Sam.) Sam. [Alismataceae, *Alismatis Rhizoma*] 12g、*Arisaema erubescens* (Wall.) Schott [Araceae, *Arisaema cum Bile*] 6g、*Citrus × aurantium* L. [Rutaceae, *Aurantii Fructus*] 12g、*Rheum palmatum* L. [Polygonaceae, *Rhei Radix et Rhizoma Praeparata*] 10g、*Fritillaria cirrhosa* D.Don [Liliaceae, *Fritillariae Cirrhosae Bulbus*] 10g、*Epimedium brevicornu* Maxim. [Berberidaceae, *Epimedii Folium*] 15g | Decoction |
