# Supplementary material for: Traditional herbal medicine for obesity-related polycystic ovary syndrome: a meta-analysis and data mining study
Source: Front Pharmacol. 2026 Jan 20;16:1738172. doi: 10.3389/fphar.2025.1738172 (PMC12864513; doi:10.3389/fphar.2025.1738172)
Supplement: Supplementary file 1 [file DataSheet1.zip › Data Sheet/~WRL0696.tmp]

**Supplementary Material**

Contents

[**Supplementary Figure S1.** Subgroup Analysis for HOMA-IR 1](#_Toc212890073)

[**Supplementary Figure S2.** Subgroup Analysis for BMI 2](#_Toc212890074)

[**Supplementary Figure S3.** Subgroup Analysis for TT 3](#_Toc212890075)

[**Supplementary Figure S4.** Subgroup Analysis for LH/FSH Ratio 4](#_Toc212890076)

[**Supplementary Figure S5.** Results of Sensitivity Analysis 5](#_Toc212890077)

[**Supplementary Figure S6.** Funnel Plots of Primary Outcomes 6](#_Toc212890078)

[**Supplementary Figure S7.** Egger's Test for Primary Outcomes 7](#_Toc212890079)

[**Supplementary Figure S8.** Trim-and-Fill Analysis for Primary Outcomes 8](#_Toc212890080)

[**Supplementary Table S1.** Search Strategy 9](#_Toc212890081)

[**Supplementary Table S2.** Methodological quality of the included studies 10](#_Toc212890082)

[**Supplementary Table S3.** GRADE evidence profile 12](#_Toc212890083)

[**Supplementary Table S4.** Herbal Formulations Used in the Included Studies 13](#_Toc212890084)

Supplementary Figure S1. Subgroup Analysis for HOMA-IR


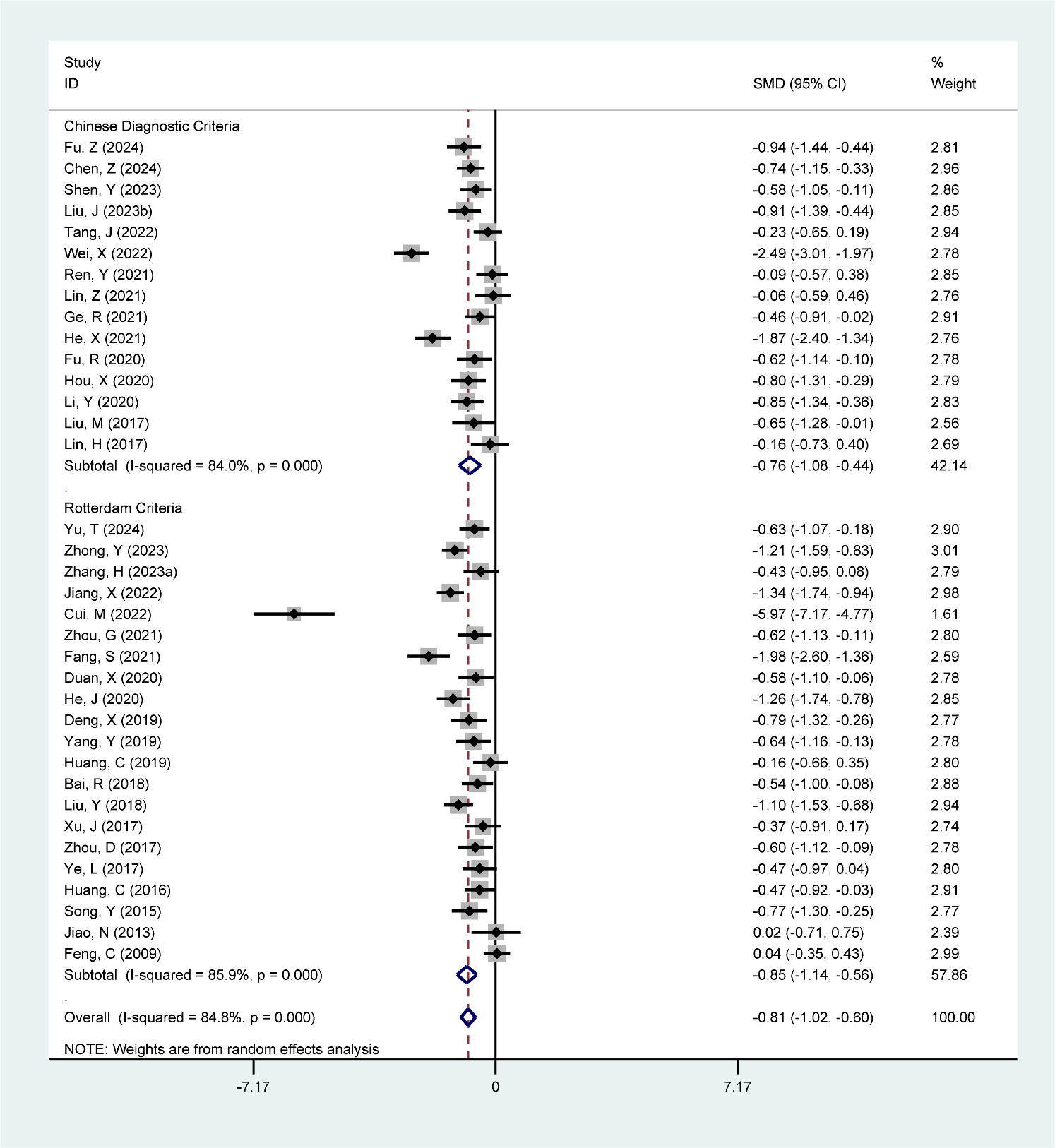


Supplementary Figure S2. Subgroup Analysis for BMI


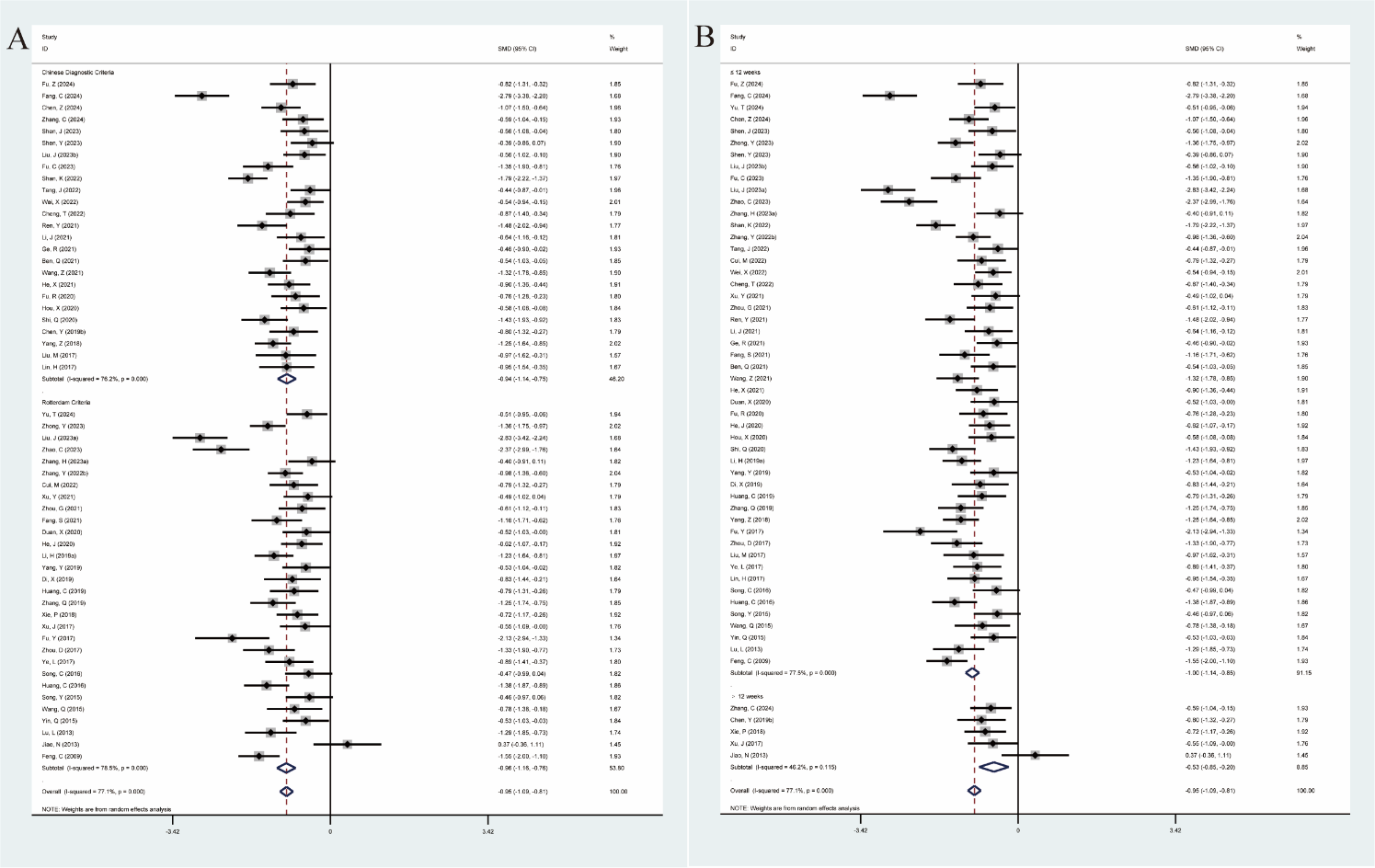


Note：(A) Subgroup analysis by diagnostic criteria. (B) Subgroup analysis by treatment duration.

Supplementary Figure S3. Subgroup Analysis for TT


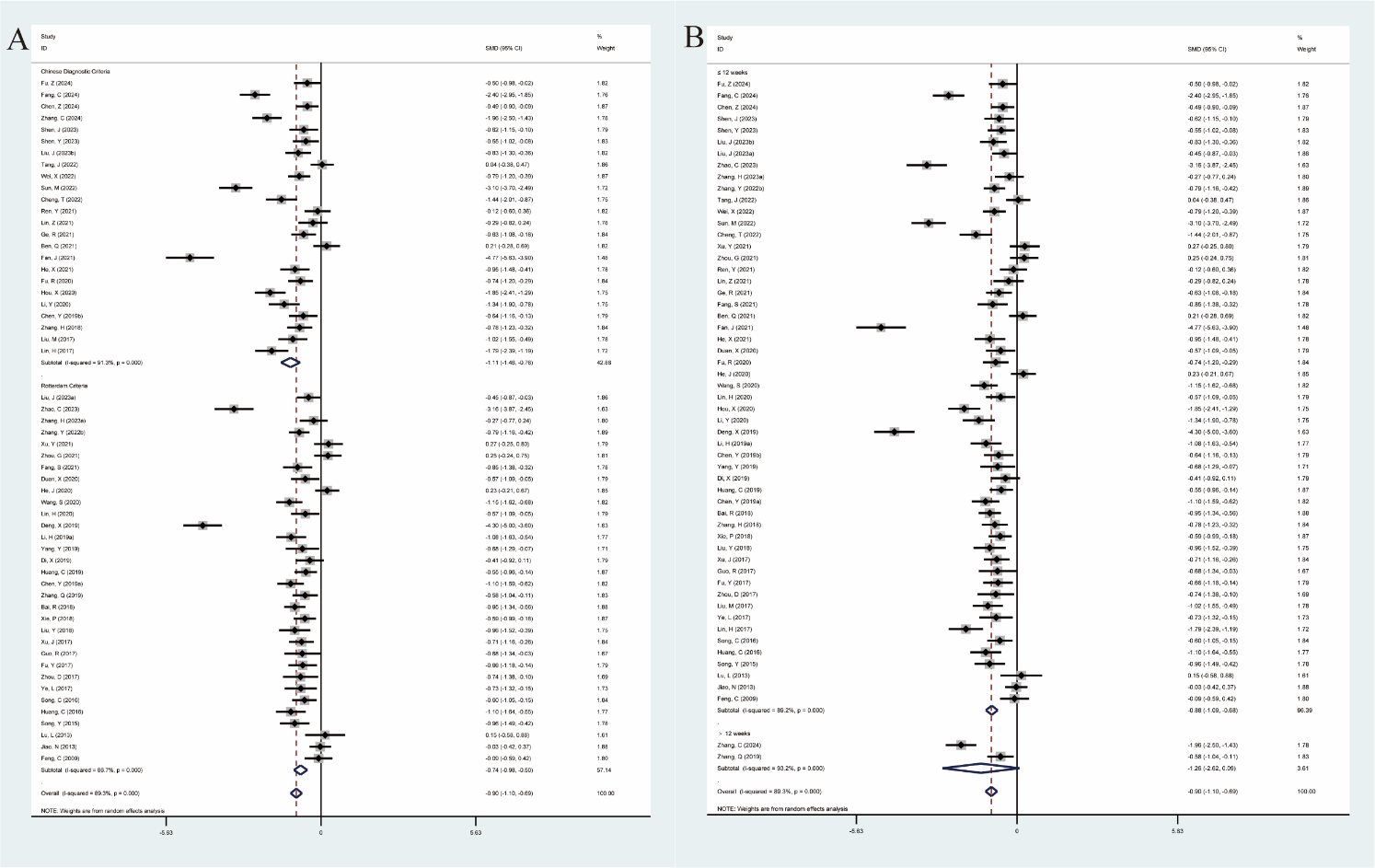


Note：(A) Subgroup analysis by diagnostic criteria. (B) Subgroup analysis by treatment duration.

Supplementary Figure S4. Subgroup Analysis for LH/FSH Ratio


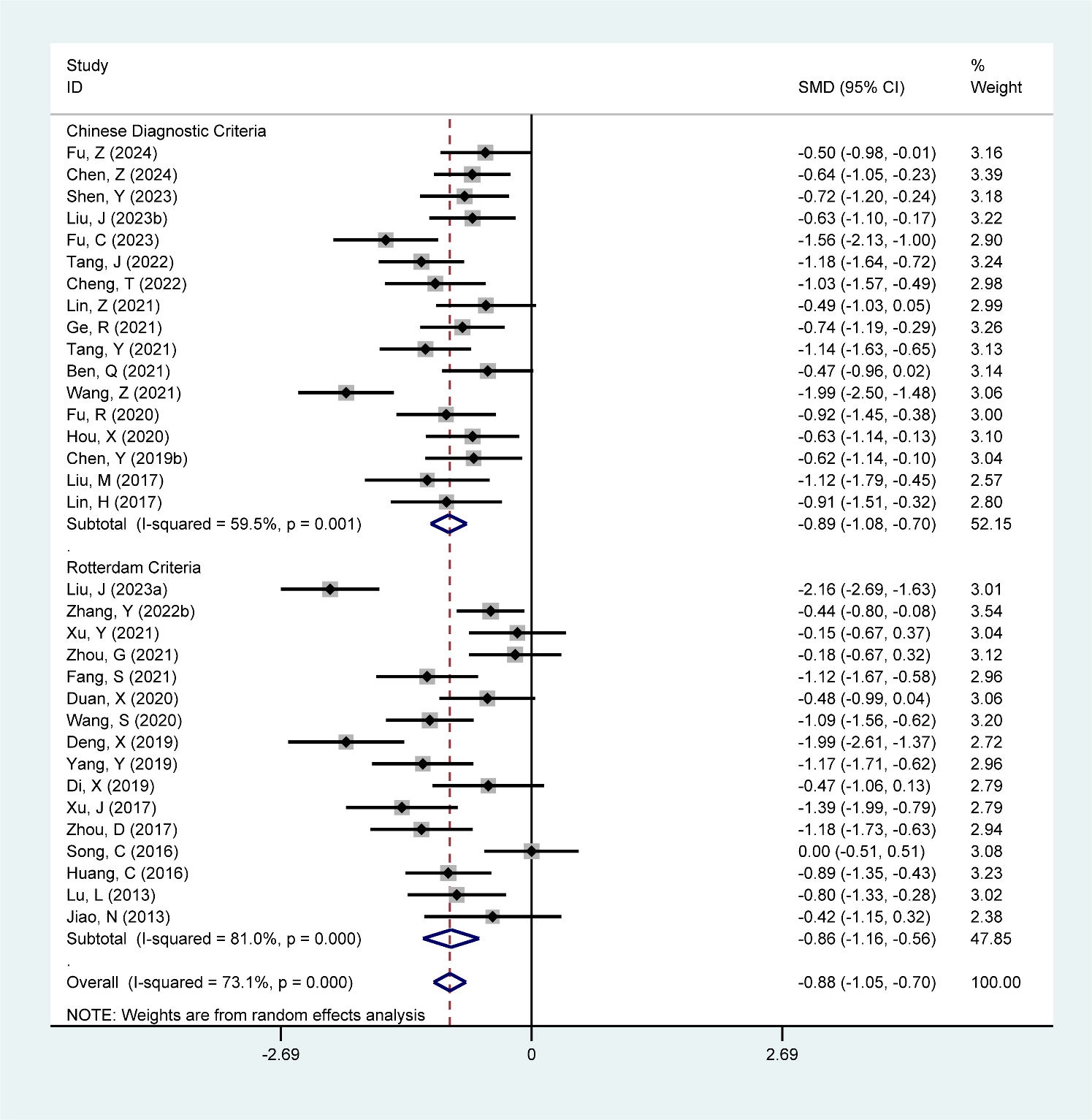


Supplementary Figure S5. Results of Sensitivity Analysis


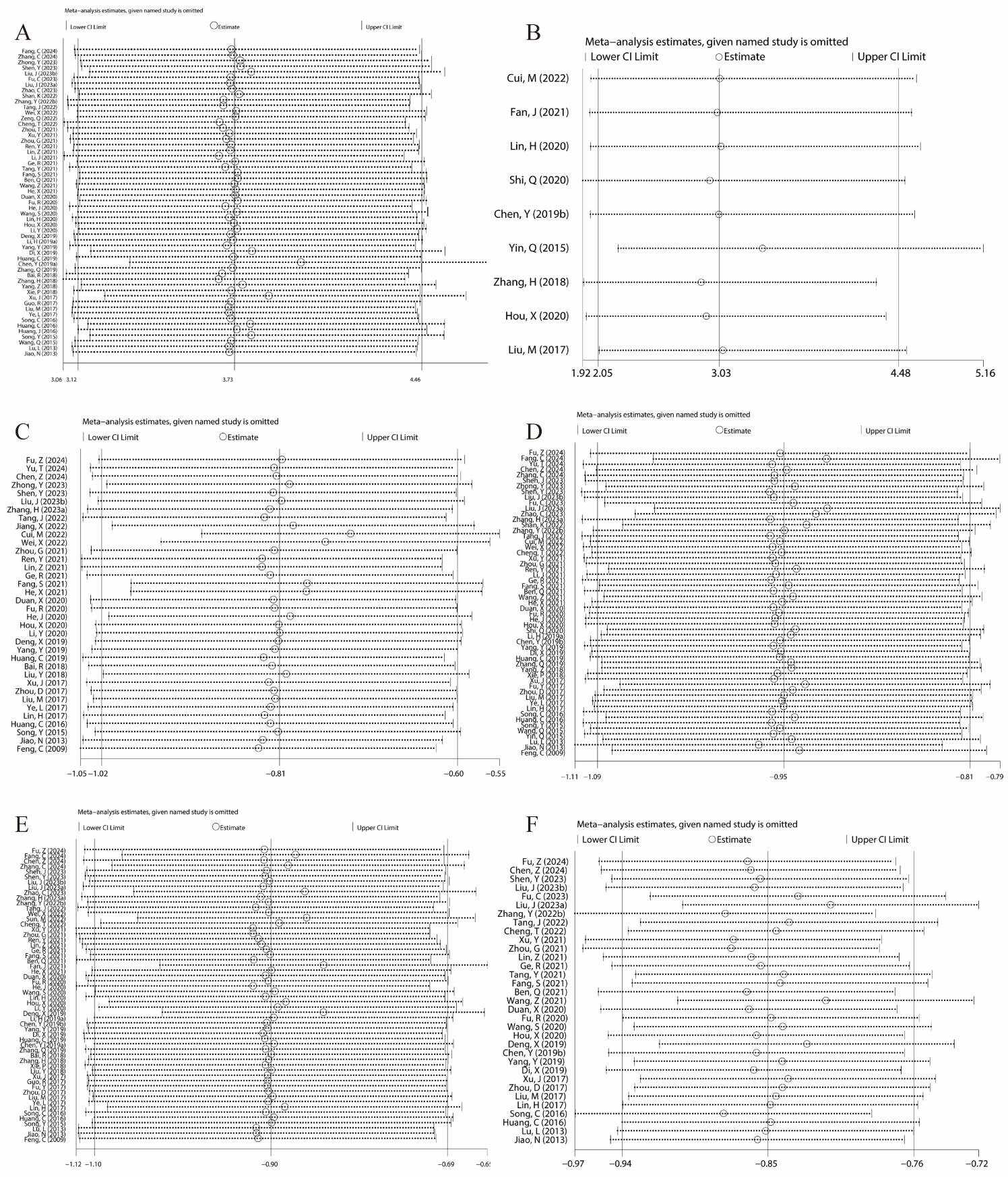


Note**:** (A) Clinical efficiency rate; (B) Clinical pregnancy rate; (C) HOMA-IR; (D) BMI; (E) Total testosterone TT; (F) LH/FSH ratio.

Supplementary Figure S6. Funnel Plots of Primary Outcomes


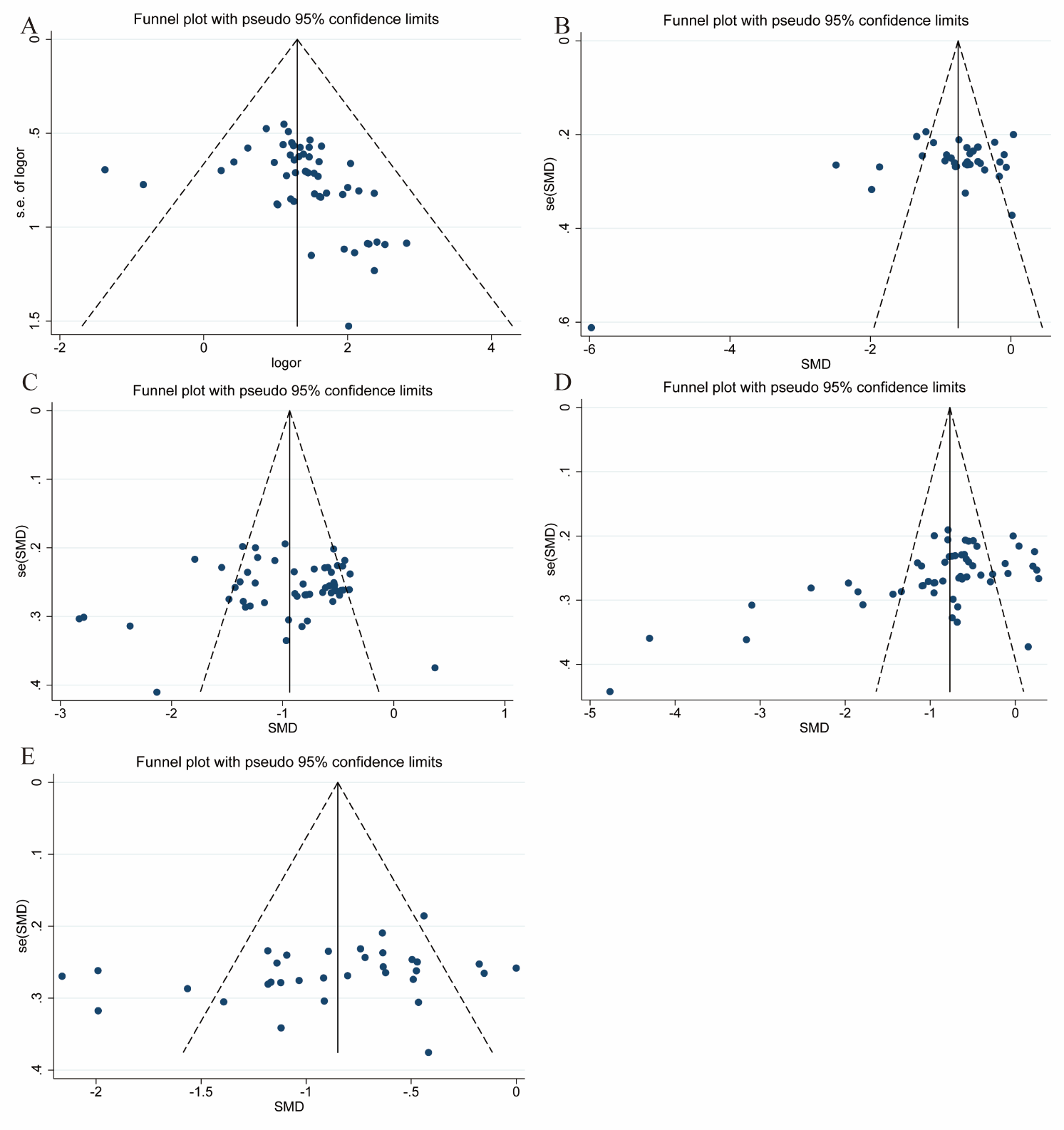


Note: (A) Clinical efficiency rate; (B) HOMA-IR; (C) BMI; (D) Total testosterone TT; (E) LH/FSH ratio.

Supplementary Figure S7. Egger's Test for Primary Outcomes


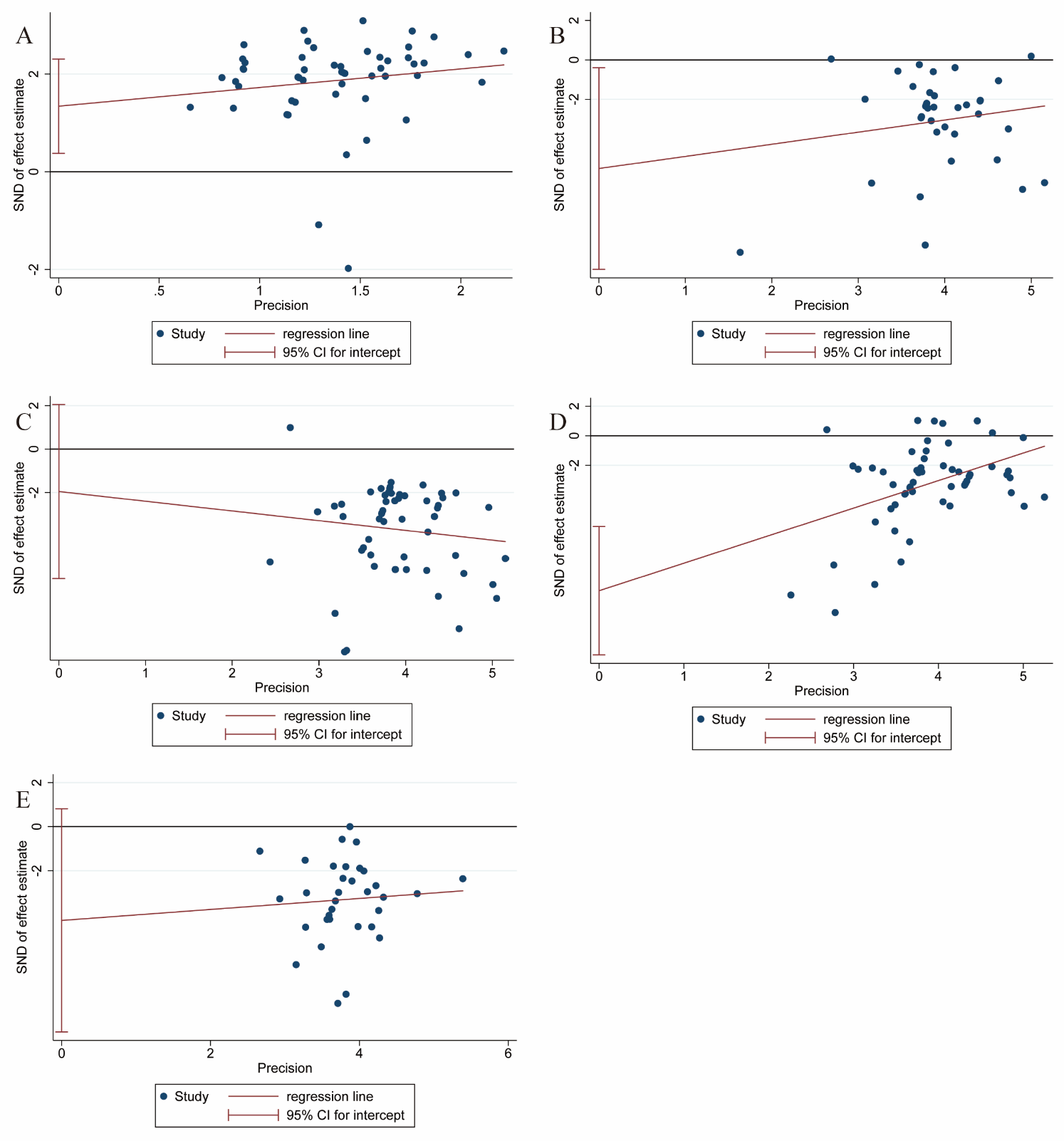


Note: (A) Clinical efficiency rate; (B) HOMA-IR; (C) BMI; (D) Total testosterone TT; (E) LH/FSH ratio.

Supplementary Figure S8. Trim-and-Fill Analysis for Primary Outcomes


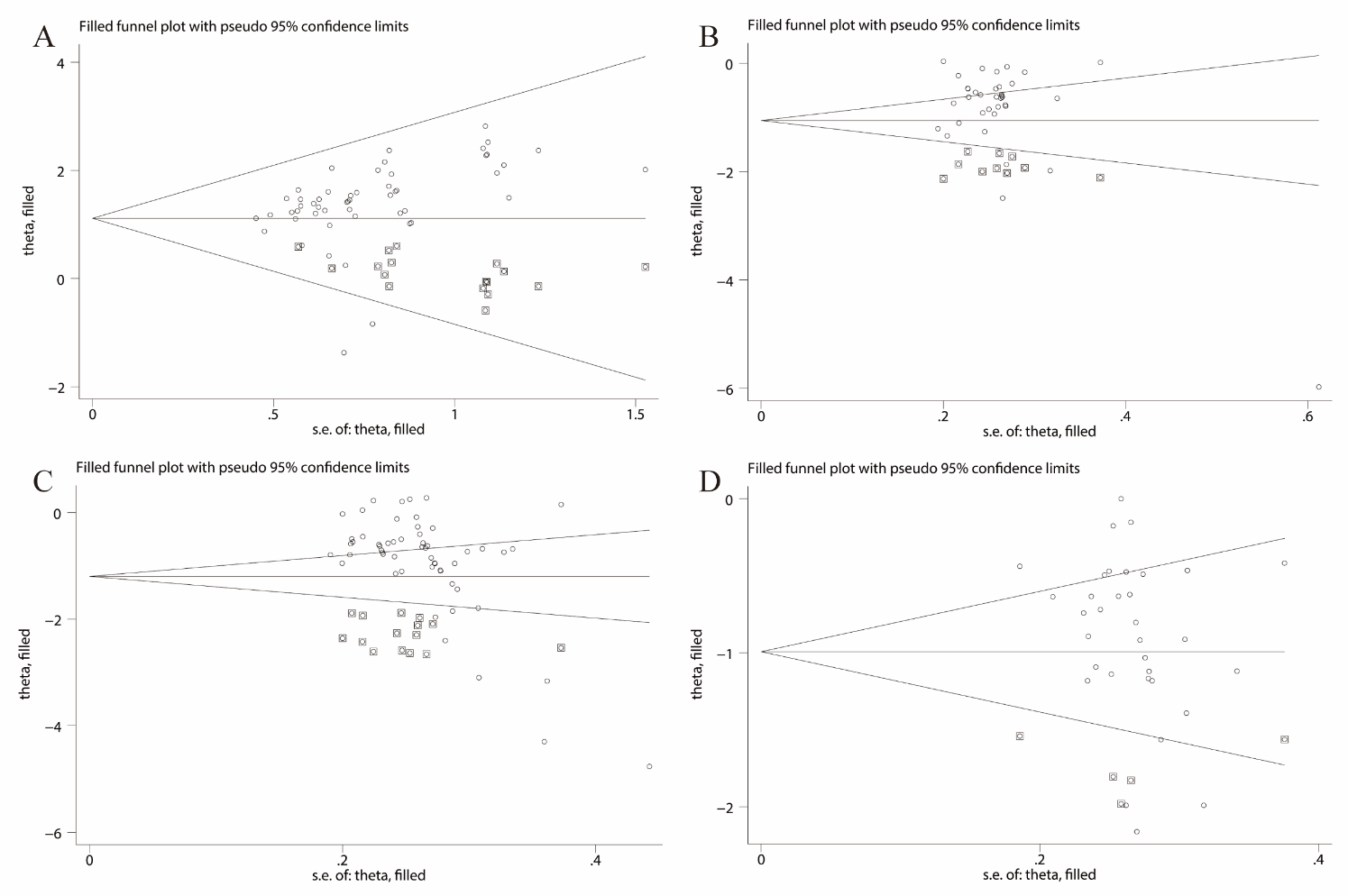


Note: (A) Clinical efficiency rate; (B) HOMA-IR; (C) Total testosterone TT; (D) LH/FSH ratio.

Supplementary Table S1. Search Strategy

| **Database** | **Search Strategies** |
| --- | --- |
| Pubmed | ("traditional chinese medicine"[Title/Abstract] OR "chinese herb"[Title/Abstract] OR "traditional chinese medicine compound"[Title/Abstract] OR "chinese patent medicine"[Title/Abstract] OR "Decoction"[Title/Abstract] OR "capsule"[Title/Abstract] OR "powder"[Title/Abstract] OR "pill"[Title/Abstract] OR "granula"[Title/Abstract]) AND ("polycystic ovary syndrome"[Title/Abstract] OR "PCOS"[Title/Abstract]) AND ("obesity"[Title/Abstract] OR "weight loss"[Title/Abstract]) |
| Web of science | Query #1=((((((((TS=(traditional chinese medicine)) OR TS=(chinese herb)) OR TS=(traditional chinese medicine compound)) OR TS=(chinese patent medicine)) OR TS=(Decoction)) OR TS=(capsule)) OR TS=(powder)) OR TS=(pill)) OR TS=(granula)  Query #2=(TS=(polycystic ovary syndrome)) OR TS=(PCOS)  Query #3=(TS=（obesity）) OR TS=(weight loss)  Query #4=#1 AND #2 AND #3 |
| Sinomed | ( "中医药"[常用字段:智能] OR "中草药"[常用字段:智能] OR "中药"[常用字段:智能] OR "中药复方"[常用字段:智能] OR "中成药"[常用字段:智能] OR "方"[常用字段:智能] OR "汤"[常用字段:智能] OR "散"[常用字段:智能] OR "颗粒"[常用字段:智能] OR "膏"[常用字段:智能]) AND "多囊卵巢综合征"[常用字段:智能] AND( "肥胖"[常用字段:智能] OR "减重"[常用字段:智能]) AND( "临床研究"[常用字段:智能] OR "临床观察"[常用字段:智能]) |
| CNKI | #1 SU=中医药 + 中草药 + 中药 + 中药复方 + 方 + 汤 + 散 + 颗粒 + 膏  #2 SU=多囊卵巢综合征  #3 SU=肥胖 + 减重  #4 SU=临床研究 + 临床观察  #5 #1 AND #2 AND #3 AND #4 |
| Wanfang | 主题:(中医药 OR 中草药 OR 中药 OR 中药复方 OR 中成药 OR 方 OR 汤 OR 散 OR 颗粒 OR 胶囊 OR 膏) and 主题:(肥胖) and 主题:(多囊卵巢综合征) and 主题:(临床研究 OR 临床观察) |
| VIP | (((((((((((((题名或关键词=中医药 OR 题名或关键词=中草药) OR 题名或关键词=中药) OR 题名或关键词=中药复方) OR 题名或关键词=中成药) OR 题名或关键词=方) OR 题名或关键词=汤) OR 题名或关键词=散) OR 题名或关键词=颗粒) OR 题名或关键词=胶囊) OR 题名或关键词=膏) AND 题名或关键词=肥胖) AND 题名或关键词=多囊卵巢综合征) AND (题名或关键词=临床研究 OR 题名或关键词=临床观察)) |

Supplementary Table S2. Methodological quality of the included studies

| Study ID | D1 | D2 | D3 | D4 | D5 | Overall |
| --- | --- | --- | --- | --- | --- | --- |
| Fu, Z (2024) | Low | Low | Low | Low | Some concerns | Some concerns |
| Fang, C (2024) | Low | Low | Low | Low | Some concerns | Some concerns |
| Yu, T (2024) | Low | Low | Low | Low | Some concerns | Some concerns |
| Chen, Z (2024) | Low | Low | Low | Low | Some concerns | Some concerns |
| Zhang, C (2024) | High | Low | Low | Low | Some concerns | High |
| Shen, J (2023) | Low | Low | Low | Low | Some concerns | Some concerns |
| Zhong, Y (2023) | Low | Low | Low | Low | Some concerns | Some concerns |
| Shen, Y (2023) | Low | Low | Low | Low | Some concerns | Some concerns |
| Liu, J (2023b) | Some concerns | Low | Low | Low | Some concerns | Some concerns |
| Fu, C (2023) | Some concerns | Low | Low | Low | Some concerns | Some concerns |
| Liu, J (2023a) | Low | Low | Low | Low | Some concerns | Some concerns |
| Zhao, C (2023) | Low | Low | Low | Low | Some concerns | Some concerns |
| Zhang, H (2023a) | Low | Low | Low | Low | Some concerns | Some concerns |
| Shan, K (2022) | Low | Low | Low | Low | Some concerns | Some concerns |
| Zhang, Y (2022b) | Low | Low | Low | Low | Some concerns | Some concerns |
| Tang, J (2022) | Low | Low | Low | Low | Some concerns | Some concerns |
| Jiang, X (2022) | Some concerns | Low | Low | Low | Some concerns | Some concerns |
| Cui, M (2022) | Some concerns | Low | Low | Low | Some concerns | Some concerns |
| Wei, X (2022) | Low | Low | Low | Low | Some concerns | Some concerns |
| Sun, M (2022) | Low | Low | Low | Low | Some concerns | Some concerns |
| Zeng, Q (2022) | Some concerns | Low | Low | Low | Some concerns | Some concerns |
| Cheng, T (2022) | Some concerns | Low | Low | Low | Some concerns | Some concerns |
| Zhou, T (2021) | Low | Low | Low | Low | Some concerns | Some concerns |
| Xu, Y (2021) | Low | Low | Low | Low | Some concerns | Some concerns |
| Zhou, G (2021) | Low | Low | Low | Low | Some concerns | Some concerns |
| Ren, Y (2021) | Some concerns | Low | Low | Low | Some concerns | Some concerns |
| Lin, Z (2021) | Low | Low | Low | Low | Some concerns | Some concerns |
| Li, J (2021) | Low | Low | Low | Low | Some concerns | Some concerns |
| Ge, R (2021) | Low | Low | Low | Low | Some concerns | Some concerns |
| Tang, Y (2021) | Low | Low | Low | Low | Some concerns | Some concerns |
| Fang, S (2021) | Low | Low | Low | Low | Some concerns | Some concerns |
| Ben, Q (2021) | Some concerns | Low | Low | Low | Some concerns | Some concerns |
| Fan, J (2021) | Some concerns | Low | Low | Low | Some concerns | Some concerns |
| Wang, Z (2021) | High | Low | Low | Low | Some concerns | High |
| He, X (2021) | Low | Low | Low | Low | Some concerns | Some concerns |
| Duan, X (2020) | Some concerns | Low | Low | Low | Some concerns | Some concerns |
| Fu, R (2020) | Low | Low | Low | Low | Some concerns | Some concerns |
| He, J (2020) | Some concerns | Low | Low | Low | Some concerns | Some concerns |
| Wang, S (2020) | Some concerns | Low | Low | Low | Some concerns | Some concerns |
| Lin, H (2020) | Some concerns | Low | Low | Low | Some concerns | Some concerns |
| Hou, X (2020) | Some concerns | Low | Low | Low | Some concerns | Some concerns |
| Shi, Q (2020) | Low | Low | Low | Low | Some concerns | Some concerns |
| Li, Y (2020) | Low | Low | Low | Low | Some concerns | Some concerns |
| Deng, X (2019) | Some concerns | Low | Low | Low | Some concerns | Some concerns |
| Li, H (2019a) | Low | Low | Low | Low | Some concerns | Some concerns |
| Chen, Y (2019b) | Some concerns | Low | Low | Low | Some concerns | Some concerns |
| Yang, Y (2019) | Low | Low | Low | Low | Some concerns | Some concerns |
| Di, X (2019) | High | Low | Low | Low | Some concerns | High |
| Huang, C (2019) | Some concerns | Low | Low | Low | Some concerns | Some concerns |
| Chen, Y (2019a) | Low | Low | Low | Low | Some concerns | Some concerns |
| Zhang, Q (2019) | Some concerns | Low | Low | Low | Some concerns | Some concerns |
| Bai, R (2018) | Low | Low | Low | Low | Some concerns | Some concerns |
| Zhang, H (2018) | Low | Low | Low | Low | Some concerns | Some concerns |
| Yang, Z (2018) | High | Low | Low | Low | Some concerns | High |
| Xie, P (2018) | Low | Low | Low | Low | Some concerns | Some concerns |
| Liu, Y (2018) | Low | Low | Low | Low | Some concerns | Some concerns |
| Xu, J (2017) | Low | Low | Low | Low | Some concerns | Some concerns |
| Guo, R (2017) | Some concerns | Low | Low | Low | Some concerns | Some concerns |
| Fu, Y (2017) | Some concerns | Low | Low | Low | Some concerns | Some concerns |
| Zhou, D (2017) | Low | Low | Low | Low | Some concerns | Some concerns |
| Liu, M (2017) | Low | Low | Low | Low | Some concerns | Some concerns |
| Ye, L (2017) | Low | Low | Low | Low | Some concerns | Some concerns |
| Lin, H (2017) | Low | Low | Low | Low | Some concerns | Some concerns |
| Song, C (2016) | Low | Low | Low | Low | Some concerns | Some concerns |
| Huang, C (2016) | Low | Low | Low | Low | Some concerns | Some concerns |
| Huang, J (2016) | Some concerns | Low | Low | Low | Some concerns | Some concerns |
| Song, Y (2015) | Low | Low | Low | Low | Some concerns | Some concerns |
| Wang, Q (2015) | Some concerns | Low | Low | Low | Some concerns | Some concerns |
| Yin, Q (2015) | Some concerns | Low | Low | Low | Some concerns | Some concerns |
| Lu, L (2013) | Some concerns | Low | Low | Low | Some concerns | Some concerns |
| Jiao, N (2013) | Some concerns | Low | Low | Low | Some concerns | Some concerns |
| Feng, C (2009) | Low | Low | Low | Low | Some concerns | Some concerns |

Note: Domains of bias assessment: D1, Randomization process; D2, Deviations from intended interventions; D3, Missing outcome data; D4, Measurement of the outcome; D5, Selection of the reported result.

Supplementary Table S3. GRADE evidence profile

| **Certainty assessment** | | | | | | | **№ of patients** | | **Effect** | | **Certainty** | **Importance** |
| --- | --- | --- | --- | --- | --- | --- | --- | --- | --- | --- | --- | --- |
| **№ of studies** | **Study design** | **Risk of bias** | **Inconsistency** | **Indirectness** | **Imprecision** | **Other considerations** | **[Chinese herbal formula+conventional pharmacotherapy]** | **[conventional pharmacotherapy]** | **Relative (95% CI)** | **Absolute (95% CI)** |  |  |
| **Clinical Efficacy Rate** | | | | | | | | | | | | |
| 55 | randomised trials | serious^a^ | not serious | not serious | not serious | publication bias strongly suspected^b^ | 1853/2047 (90.5%) | 1469/2027 (72.5%) | **OR 3.73** (3.12 to 4.46) | **183 more per 1,000** (from 167 more to 197 more) | ⨁⨁◯◯ Low^a,b^ | CRITICAL |
| **Clinical Pregnancy Rate** | | | | | | | | | | | | |
| 9 | randomised trials | serious^a^ | not serious | not serious | not serious | none | 118/288 (41.0%) | 58/288 (20.1%) | **OR 3.03** (2.05 to 4.48) | **232 more per 1,000** (from 139 more to 329 more) | ⨁⨁⨁◯ Moderate^a^ | CRITICAL |
| **BMI** | | | | | | | | | | | | |
| 55 | randomised trials | serious^a^ | serious^c^ | not serious | not serious | none | 1984 | 1965 | - | SMD **0.95 SD lower** (1.09 lower to 0.81 lower) | ⨁⨁◯◯ Low^a,c^ | IMPORTANT |
| **TT** | | | | | | | | | | | | |
| 56 | randomised trials | serious^a^ | serious^c^ | not serious | not serious | publication bias strongly suspected^b^ | 2012 | 1988 | - | SMD **0.9 SD lower** (1.1 lower to 0.69 lower) | ⨁◯◯◯ Very low^a,b,c^ | NOT IMPORTANT |
| **LH/FSH ratio** | | | | | | | | | | | | |
| 33 | randomised trials | serious^a^ | serious^c^ | not serious | not serious | none | 1109 | 1089 | - | SMD **0.88 SD lower** (1.05 lower to 0.7 lower) | ⨁⨁◯◯ Low^a,c^ | NOT IMPORTANT |
| **HOMA-IR** | | | | | | | | | | | | |
| 36 | randomised trials | serious^a^ | serious^c^ | not serious | not serious | publication bias strongly suspected^b^ | 1300 | 1278 | - | SMD **0.81 SD lower** (1.02 lower to 0.6 lower) | ⨁◯◯◯ Very low^a,b,c^ | IMPORTANT |

Note: Reasons for downgrading: a, The included trials were at some risk of bias due to inadequacies in randomization or blinding; b, Considerable heterogeneity was observed, and confidence intervals showed poor overlap; C, Publication bias was suspected based on observable funnel plot asymmetry, which was confirmed by a significant Egger's test.

Supplementary Table S4. Herbal Formulations Used in the Included Studies

| Study | Formulation Name | Herbal Composition | Dosage Form |
| --- | --- | --- | --- |
| Fu, Z (2024) | Erxian Qiling Decoction | *Epimedium brevicornu* Maxim. 12g, *Curculigo orchioides* Gaertn. 9g, *Astragalus membranaceus* (Fisch.) Bge. var. *mongholicus* (Bge.) Hsiao 30g, Poriae Cutis 30g, *Angelica sinensis* (Oliv.) Diels 12g, *Morinda officinalis* How. 12g, *Phellodendron chinense* Schneid. 30g, *Anemarrhena asphodeloides* Bge. 30g, Rehmanniae Radix Praeparata 12g, *Ligustrum lucidum* Ait. 15g, *Dipsacus asper* Wall. ex Henry 15g, *Citrus aurantium* L. 12g, *Cyperus rotundus* L. 12g, *Sparganium stoloniferum* Buch.-Ham. 12g, Curcuma phaeocaulis Val. 12g, *Ephedra sinica* Stapf 12g, *Paeonia lactiflora* Pall. 30g, *Glycyrrhiza uralensis* Fisch. 12g | Decoction |
| Fang, C (2024) | Modified Cangfu Daotan Decoction | *Atractylodes lancea* (Thunb.) DC. 15g, *Cyperus rotundus* L. 15g, *Poria cocos* (Schw.) Wolf 10g, *Citrus reticulata* Blanco 10g, *Prunus persica* (L.) Batsch 10g, *Pinellia ternata* (Thunb.) Breit. 6g, *Citrus aurantium* L. 6g, *Arisaema erubescens* (Wall.) Schott 6g, Massa Medicata Fermentata 6g, *Angelica sinensis* (Oliv.) Diels 6g, *Prunella vulgaris* L. 6g, *Ligusticum chuanxiong* Hort. 6g, *Glycyrrhiza uralensis* Fisch. 6g, *Zingiber officinale* Rosc. 3g | Decoction |
| Yu, T (2024) | Gexia Zhuyu Decoction | *Prunus persica* (L.) Batsch 9g, *Carthamus tinctorius* L. 9g, *Angelica sinensis* (Oliv.) Diels 9g, *Ligusticum chuanxiong* Hort. 6g, Paeoniae Radix Rubra 6g, *Cyperus rotundus* L. 4.5g, *Citrus aurantium* L. 4.5g, *Lindera aggregata* (Sims) Kos-term. 9g, Trogopterori Faeces 6g, *Paeonia suffruticosa* Andr. 6g, *Corydalis yanhusuo* W.T.Wang 3g, *Glycyrrhiza uralensis* Fisch. 9g | Decoction |
| Chen, Z (2024) | Huazhuo Jiedu Decoction | *Eupatorium fortunei* Turcz. 15g, *Salvia miltiorrhiza* Bunge 15g, Paeoniae Radix Rubra 15g, *Coix lacryma-jobi* L. var. ma-yuen (Roman.) Stapf 15g, *Atractylodes lancea* (Thunb.) DC. 12g, *Smilax glabra* Roxb. 20g, *Astragalus membranaceus* (Fisch.) Bge. var. *mongholicus* (Bge.) Hsiao 30g, Silkworm Feces 10g | Decoction |
| Zhang, C (2024) | Self-formulated Prescription | *Codonopsis pilosula* (Franch.) Nannf. 12g, *Atractylodes macrocephala* Koidz. 15g, *Poria cocos* (Schw.) Wolf 12g, *Psoralea corylifolia* L. 10g, Rehmanniae Radix Praeparata 18g, *Dioscorea opposita* Thunb. 18g, *Dipsacus asper* Wall. ex Henry 10g, *Epimedium brevicornu* Maxim. 10g, *Cuscuta australis* R.Br. 10g, *Leonurus japonicus* Houtt. 15g, Paeoniae Radix Rubra 10g, Pinelliae Rhizoma Praeparatum 8g, *Citrus reticulata* Blanco 12g, *Monascus purpureus* Went 8g, *Acorus tatarinowii* Schott 10g, *Glycyrrhiza uralensis* Fisch. 6g | Decoction |
| Shen, J (2023) | Dachaihu Decoction combined with Fangji Huangqi Decoction | *Bupleurum chinense* DC. 18g, *Scutellaria baicalensis* Georgi 10g, *Pinellia ternata* (Thunb.) Breit. 12g, *Citrus aurantium* L. 12g, *Paeonia lactiflora* Pall. 15g, *Rheum palmatum* L. 10g, *Zingiber officinale* Roscoe 5g, *Ziziphus jujuba* Mill. 15g, *Stephania tetrandra* S. Moore 12g, *Astragalus membranaceus* (Fisch.) Bge. var. *mongholicus* (Bge.) Hsiao 30g, *Atractylodes macrocephala* Koidz. 15g, *Glycyrrhiza uralensis* Fisch. 5g | Decoction |
| Zhong, Y (2023) | Fangfeng Tongsheng Decoction | *Platycodon grandiflorum* (Jacq.) A.DC. 20g, Talcum 20g, *Scutellaria baicalensis* Georgi 20g, Gypsum Fibrosum 20g, *Ligusticum chuanxiong* Hort. 15g, *Angelica sinensis* (Oliv.) Diels 15g, *Mentha haplocalyx* Briq. 15g, Natrii Sulfas 15g, *Paeonia lactiflora* Pall. 15g, Forsythia suspensa (Thunb.) Vahl 15g, Saposhnikovia divaricata (Turcz.) Schischk. 15g, *Rheum palmatum* L. 10g, *Glycyrrhiza uralensis* Fisch. 10g, *Atractylodes macrocephala* Koidz. 6g, *Gardenia jasminoides* Ellis 6g, *Cimicifuga heracleifolia* Kom. 6g, *Schizonepeta tenuisfolia* Briq. 6g | Decoction |
| Shen, Y (2023) | Qutan Lishi Decoction | *Atractylodes lancea* (Thunb.) DC. 9g, *Cyperus rotundus* L. 9g, *Citrus aurantium* L. 9g, *Citrus reticulata* Blanco 9g, *Poria cocos* (Schw.) Wolf 9g, *Pinellia ternata* (Thunb.) Breit. 9g, *Atractylodes macrocephala* Koidz. 9g, *Zingiber officinale* Rosc. 3g, *Taxillus chinensis* (DC.) Danser 9g, *Crataegus pinnatifida* Bge. 15g, *Prunus persica* (L.) Batsch 9g, *Carthamus tinctorius* L. 9g, *Glycyrrhiza uralensis* Fisch. 9g | Decoction |
| Liu, J (2023b) | Self-formulated Prescription | *Atractylodes lancea* (Thunb.) DC. 20g, Pinelliae Rhizoma Praeparatum cum Alumine 15g, *Citrus reticulata* Blanco 15g, *Poria cocos* (Schw.) Wolf 15g, *Coix lacryma-jobi* L. var. ma-yuen (Roman.) Stapf 20g, *Atractylodes macrocephala* Koidz. 15g, *Fritillaria thunbergii* Miq. 20g, *Crataegus pinnatifida* Bge. 15g, *Cyathula officinalis* Kuan 20g, *Salvia miltiorrhiza* Bunge 20g, *Angelica sinensis* (Oliv.) Diels 15g, *Ligusticum chuanxiong* Hort. 15g | Decoction |
| Fu, C (2023) | Yinang Jianzhi Decoction | *Atractylodes lancea* (Thunb.) DC. 15g, *Cyperus rotundus* L. 15g, *Prunus persica* (L.) Batsch 10g, *Citrus reticulata* Blanco 10g, *Citrus aurantium* L. 10g, *Magnolia officinalis* Rehd. et Wils. 10g, *Dioscorea opposita* Thunb. 10g, Fluoritum 10g, Cervi Cornu Praeparatum 15g, Pinelliae Rhizoma Praeparatum 15g, Arisaema cum Bile 12g, *Crataegus pinnatifida* Bge. 10g, *Monascus purpureus* Went 6g | Decoction |
| Liu, J (2023a) | Modified Danxi Zhishitan Decoction | *Atractylodes lancea* (Thunb.) DC. 15g, Pinelliae Rhizoma Praeparatum 15g, *Atractylodes macrocephala* Koidz. 10g, *Poria cocos* (Schw.) Wolf 10g, Talcum 6g, *Cyperus rotundus* L. 6g, *Ligusticum chuanxiong* Hort. 6g, *Angelica sinensis* (Oliv.) Diels 6g | Decoction |
| Zhao, C (2023) | Bushen Huatan Decoction | Rehmanniae Radix Praeparata 25g, *Poria cocos* (Schw.) Wolf 15g, *Dioscorea opposita* Thunb. 15g, *Coix lacryma-jobi* L. var. ma-yuen (Roman.) Stapf 25g, *Euryale ferox* Salisb. 25g, *Cornus officinalis* Sieb. et Zucc. 15g, *Schisandra chinensis* (Turcz.) Baill. 5g, *Ophiopogon japonicus* (L.f) Ker-Gawl. 15g, *Plantago asiatica* L. 5g, *Alpinia oxyphylla* Miq. 5g | Decoction |
| Zhang, H (2023a) | Qigong Decoction | *Citrus reticulata* Blanco, *Pinellia ternata* (Thunb.) Breit., *Poria cocos* (Schw.) Wolf, *Atractylodes macrocephala* Koidz., *Cyperus rotundus* L., *Ligusticum chuanxiong* Hort., Massa Medicata Fermentata, *Glycyrrhiza uralensis* Fisch. | Decoction |
| Shan, K (2022) | Cangfu Daotan Decoction | *Astragalus membranaceus* (Fisch.) Bge. var. *mongholicus* (Bge.) Hsiao 30g, *Dioscorea opposita* Thunb. 30g, *Poria cocos* (Schw.) Wolf 30g, *Salvia miltiorrhiza* Bunge 15g, *Epimedium brevicornu* Maxim. 15g, *Gleditsia sinensis* Lam. 10g, *Cyperus rotundus* L. 10g, Pinelliae Rhizoma Praeparatum 10g, *Acorus tatarinowii* Schott 10g, *Atractylodes lancea* (Thunb.) DC. 10g, *Angelica sinensis* (Oliv.) Diels 10g, *Citrus reticulata* Blanco 6g | Decoction |
| Zhang, Y (2022b) | Cangfu Daotan Decoction | *Atractylodes lancea* (Thunb.) DC. 20g, *Cyperus rotundus* L. 20g, *Citrus reticulata* Blanco 15g, *Poria cocos* (Schw.) Wolf 15g, *Arisaema erubescens* (Wall.) Schott 10g, *Citrus aurantium* L. 10g, *Pinellia ternata* (Thunb.) Breit. 10g, *Ligusticum chuanxiong* Hort. 10g, Massa Medicata Fermentata 10g | Decoction |
| Tang, J (2022) | Ditan Zhuyu Decoction | *Citrus reticulata* Blanco, *Poria cocos* (Schw.) Wolf, Pinelliae Rhizoma Praeparatum, *Crataegus pinnatifida* Bge., *Acorus tatarinowii* Schott, Arisaema cum Bile, *Sparganium stoloniferum* Buch.-Ham., Curcuma phaeocaulis Val., *Epimedium brevicornu* Maxim., *Morinda officinalis* How., *Spatholobus suberectus* Dunn, *Cinnamomum cassia* Presl | Decoction |
| Jiang, X (2022) | Gexia Zhuyu Decoction | *Prunus persica* (L.) Batsch 9g, *Carthamus tinctorius* L. 9g, *Angelica sinensis* (Oliv.) Diels 9g, *Ligusticum chuanxiong* Hort. 6g, Paeoniae Radix Rubra 6g, *Cyperus rotundus* L. 4.5g, *Citrus aurantium* L. 4.5g, *Lindera aggregata* (Sims) Kos-term. 9g, Trogopterori Faeces 6g, *Corydalis yanhusuo* W.T.Wang 3g, *Paeonia suffruticosa* Andr. 6g, *Glycyrrhiza uralensis* Fisch. 9g | Decoction |
| Cui, M (2022) | Bushen Huatan Decoction | *Epimedium brevicornu* Maxim. 10g, *Curculigo orchioides* Gaertn. 10g, *Atractylodes lancea* (Thunb.) DC. 10g, *Pinellia ternata* (Thunb.) Breit. 6g, *Citrus reticulata* Blanco 6g, *Anemone altaica* Fisch. 10g, *Cyperus rotundus* L. 10g, *Ligusticum chuanxiong* Hort. 6g, *Alisma orientale* (Sam.) Juzep. 10g, Cervi Cornu Praeparatum 10g, Arisaema cum Bile 6g, *Amomum villosum* Lour. 3g, *Atractylodes macrocephala* Koidz. 6g, *Dioscorea opposita* Thunb. 6g | Decoction |
| Wei, X (2022) | Bushen Huoxue Decoction | Rehmanniae Radix Praeparata 20g, Fluoritum 20g, *Codonopsis pilosula* (Franch.) Nannf. 20g, *Cuscuta australis* R.Br. 15g, *Lycium barbarum* L. 15g, *Epimedium brevicornu* Maxim. 15g, *Morus alba* L. 15g, *Angelica sinensis* (Oliv.) Diels 10g, *Ligustrum lucidum* Ait. 10g, Cervi Cornu 10g, *Oldenlandia diffusa* (Willd.) Roxb. 10g, *Ligusticum chuanxiong* Hort. 10g, *Cyperus rotundus* L. 10g, *Glycyrrhiza uralensis* Fisch. 10g, *Carthamus tinctorius* L. 6g | Decoction |
| Sun, M (2022) | Huoxue Qushi Bushen Decoction | Fluoritum 20g, *Crataegus pinnatifida* Bge. 20g, *Epimedium brevicornu* Maxim. 15g, *Morinda officinalis* How. 15g, *Cuscuta australis* R.Br. 15g, *Atractylodes lancea* (Thunb.) DC. 15g, *Cyperus rotundus* L. 15g, *Poria cocos* (Schw.) Wolf 15g, *Cyathula officinalis* Kuan 15g, *Citrus reticulata* Blanco 12g, Cervi Cornu Praeparatum 12g, Pinelliae Rhizoma Praeparatum cum Alumine 12g, *Gleditsia sinensis* Lam. 12g, *Angelica sinensis* (Oliv.) Diels 12g, *Zanthoxylum bungeanum* Maxim. 3g, *Glycyrrhiza uralensis* Fisch. 6g | Decoction |
| Zeng, Q (2022) | Cupailuan Decoction | *Angelica sinensis* (Oliv.) Diels 10g, *Cuscuta australis* R.Br. 10g, *Atractylodes macrocephala* Koidz. 10g, *Ligusticum chuanxiong* Hort. 10g, *Paeonia suffruticosa* Andr. 10g, *Salvia miltiorrhiza* Bunge 10g, *Coix lacryma-jobi* L. var. ma-yuen (Roman.) Stapf 10g, *Dipsacus asper* Wall. ex Henry 15g, Paeoniae Radix Rubra 15g, *Atractylodes lancea* (Thunb.) DC. 15g, *Poria cocos* (Schw.) Wolf 15g | Decoction |
| Cheng, T (2022) | Fenxiao Huoxue Decoction | *Prunus armeniaca* L. var. ansu Maxim. 15g, *Amomum kravanh* Pierre ex Gagnep. 15g, *Citrus aurantium* L. 15g, Pinelliae Rhizoma Praeparatum 10g, *Plantago asiatica* L. 10g, *Coix lacryma-jobi* L. var. ma-yuen (Roman.) Stapf 10g, *Citrus reticulata* Blanco 10g, *Magnolia officinalis* Rehd. et Wils. 10g, *Coptis chinensis* Franch. 10g, *Poria cocos* (Schw.) Wolf 10g, *Scutellaria baicalensis* Georgi 15g, *Tetrapanax papyrifer* (Hook.) K. Koch 10g, *Cyperus rotundus* L. 15g, *Ligusticum chuanxiong* Hort. 15g, *Achyranthes bidentata* Bl. 15g, Paeoniae Radix Rubra 15g | Decoction |
| Zhou, T (2021) | Jianpi Huatan Decoction | *Poria cocos* (Schw.) Wolf 15g, *Fritillaria thunbergii* Miq. 15g, *Astragalus membranaceus* (Fisch.) Bge. var. *mongholicus* (Bge.) Hsiao 10g, *Bupleurum chinense* DC. 10g, *Angelica sinensis* (Oliv.) Diels 10g, *Atractylodes lancea* (Thunb.) DC. 10g, *Citrus reticulata* Blanco 10g, Pinelliae Rhizoma Praeparatum 10g, *Acorus tatarinowii* Schott 10g, *Citrus aurantium* L. 10g, *Coptis chinensis* Franch. 5g, *Ligusticum chuanxiong* Hort. 5g, *Glycyrrhiza uralensis* Fisch. 3g | Decoction |
| Xu, Y (2021) | Modified Huanglian Wendan Decoction | *Coptis chinensis* Franch. 6g, *Poria cocos* (Schw.) Wolf 12g, Pinelliae Rhizoma Praeparatum cum Zingibere et Alumine 9g, Bambusa tuldoides Munro 12g, *Citrus aurantium* L. 12g, *Citrus reticulata* Blanco 12g, *Cuscuta australis* R.Br. 12g, Arisaema cum Bile 9g, *Trichosanthes kirilowii* Maxim. 12g, *Cyperus rotundus* L. 12g, *Taxillus chinensis* (DC.) Danser 9g, *Glycyrrhiza uralensis* Fisch. 6g | Decoction |
| Zhou, G (2021) | Qigong Pills Modified Decotion | *Citrus reticulata* Blanco 12g, *Pinellia ternata* (Thunb.) Breit. 9g, *Poria cocos* (Schw.) Wolf 20g, *Glycyrrhiza uralensis* Fisch. 6g, *Atractylodes macrocephala* Koidz. 15g, *Cyperus rotundus* L. 12g, Massa Medicata Fermentata 15g, *Atractylodes lancea* (Thunb.) DC. 15g, *Gallus gallus domesticus* Brisson 30g, *Spatholobus suberectus* Dunn 30g, *Ligusticum chuanxiong* Hort. 9g, *Angelica sinensis* (Oliv.) Diels 12g, *Leonurus japonicus* Houtt. 15g, *Lycopus lucidus* Turcz. var. hirtus Regel 15g, *Coix lacryma-jobi* L. var. ma-yuen (Roman.) Stapf 30g, *Crataegus pinnatifida* Bge. 15g, *Gallus gallus domesticus* Brisson 15g | Granula |
| Ren, Y (2021) | Shoushen Tiaojing Decoction | *Atractylodes lancea* (Thunb.) DC. 10g, *Cyperus rotundus* L. 10g, *Poria cocos* (Schw.) Wolf 10g, *Citrus reticulata* Blanco 6g, *Pinellia ternata* (Thunb.) Breit. 6g, *Citrus aurantium* L. 6g, Leonuri Fructus 10g, *Taxillus chinensis* (DC.) Danser 10g, *Crataegus pinnatifida* Bge. 10g, *Salvia miltiorrhiza* Bunge 10g, *Plantago asiatica* L. 15g, *Achyranthes bidentata* Bl. 10g, *Glycyrrhiza uralensis* Fisch. 3g | Granula |
| Lin, Z (2021) | Modified Pingwei San | *Atractylodes lancea* (Thunb.) DC. 16g, *Magnolia officinalis* Rehd. et Wils. 12g, *Citrus reticulata* Blanco 10g, *Zingiber officinale* Rosc. 10g, *Bupleurum chinense* DC. 10g, *Paeonia lactiflora* Pall. 15g, *Citrus aurantium* L. 10g, *Glycyrrhiza uralensis* Fisch. 6g, *Ziziphus jujuba* Mill. 15g, *Eupatorium fortunei* Turcz. 15g, *Sinapis alba* L. 15g, *Cuscuta australis* R.Br. 30g, *Euonymus alatus* (Thunb.) Siebold 15g | Granula |
| Li, J (2021) | Jianpi Huatan Decoction | *Poria cocos* (Schw.) Wolf 15g, *Fritillaria thunbergii* Miq. 15g, *Astragalus membranaceus* (Fisch.) Bge. var. *mongholicus* (Bge.) Hsiao 10g, *Bupleurum chinense* DC. 10g, *Angelica sinensis* (Oliv.) Diels 10g, *Atractylodes lancea* (Thunb.) DC. 10g, *Citrus reticulata* Blanco 10g, Pinelliae Rhizoma Praeparatum 10g, *Acorus tatarinowii* Schott 10g, *Citrus aurantium* L. 10g, *Coptis chinensis* Franch. 5g, *Ligusticum chuanxiong* Hort. 5g, *Glycyrrhiza uralensis* Fisch. 3g | Decoction |
| Ge, R (2021) | Modified Erchen Decoction combined with Gegen Decoction | *Citrus reticulata* Blanco 12g, *Poria cocos* (Schw.) Wolf 15g, Pinelliae Rhizoma Praeparatum cum Alumine 12g, Rehmanniae Radix Praeparata 10g, *Cornus officinalis* Sieb. et Zucc. 10g, *Cuscuta australis* R.Br. 12g, *Angelica sinensis* (Oliv.) Diels 15g, *Ephedra sinica* Stapf 5g, *Cinnamomum cassia* Presl 12g, *Pueraria lobata* (Willd.) Ohwi 15g, *Notopterygium incisum* Ting ex H. T. Chang 10g, *Zingiber officinale* Rosc. 5g, *Glycyrrhiza uralensis* Fisch. 6g | Decoction |
| Tang, Y (2021) | Yinang Zhuyun Decoction | *Atractylodes lancea* (Thunb.) DC. 15g, *Citrus aurantium* L. 10g, *Cyperus rotundus* L. 15g, *Prunus persica* (L.) Batsch 10g, *Citrus reticulata* Blanco 10g, *Magnolia officinalis* Rehd. et Wils. 10g, Pinelliae Rhizoma Praeparatum 15g, Arisaema cum Bile 12g, *Crataegus pinnatifida* Bge. 10g, *Monascus purpureus* Went 6g, Fluoritum 10g, Cervi Cornu Praeparatum 15g | Decoction |
| Fang, S (2021) | Shenling Baizhu Powder | *Panax ginseng* C. A. Mey. 10g, *Atractylodes macrocephala* Koidz. 15g, *Poria cocos* (Schw.) Wolf 20g, *Platycodon grandiflorum* (Jacq.) A.DC. 15g, *Nelumbo nucifera* Gaertn. 15g, *Coix lacryma-jobi* L. var. ma-yuen (Roman.) Stapf 20g, *Dioscorea opposita* Thunb. 30g, *Dolichos lablab* L. 15g, *Glycyrrhiza uralensis* Fisch. 5g | Granula |
| Ben, Q (2021) | Yishen Xiaotan Decoction | *Cuscuta australis* R.Br. 20g, Rehmanniae Radix Praeparata 20g, *Taxillus chinensis* (DC.) Danser 15g, *Atractylodes macrocephala* Koidz. 12g, *Dolichos lablab* L. 15g, *Poria cocos* (Schw.) Wolf 15g, *Citrus reticulata* Blanco 10g, *Salvia miltiorrhiza* Bunge 20g, *Angelica sinensis* (Oliv.) Diels 12g, *Spatholobus suberectus* Dunn 15g, *Cyperus rotundus* L. 12g, *Lycopus lucidus* Turcz. var. hirtus Regel 12g, *Plantago asiatica* L. 15g, *Benincasa hispida* (Thunb.) Cogn. 15g, *Phragmites communis* Trin. 12g, *Pueraria lobata* (Willd.) Ohwi 12g | Decoction |
| Fan, J (2021) | Modified Cangfu Daotan Decoction | *Atractylodes lancea* (Thunb.) DC., *Cyperus rotundus* L., *Citrus aurantium* L., *Citrus reticulata* Blanco, *Poria cocos* (Schw.) Wolf, *Glycyrrhiza uralensis* Fisch., *Zingiber officinale* Rosc., Massa Medicata Fermentata, Pinelliae Rhizoma Praeparatum, *Angelica sinensis* (Oliv.) Diels, *Morinda officinalis* How., *Lycium barbarum* L., *Cuscuta australis* R.Br., *Epimedium brevicornu* Maxim., *Eucommia ulmoides* Oliv. | Decoction |
| Wang, Z (2021) | Self-formulated Prescription | Arisaema cum Bile 15g, *Atractylodes lancea* (Thunb.) DC. 20g, *Citrus aurantium* L. 15g, *Cyperus rotundus* L. 10g, *Pinellia ternata* (Thunb.) Breit. 15g, *Citrus reticulata* Blanco 10g, *Poria cocos* (Schw.) Wolf 15g, *Zingiber officinale* Rosc. 10g, *Glycyrrhiza uralensis* Fisch. 10g | Decoction |
| He, X (2021) | Jianpi Bushen Huoxue Decoction | *Codonopsis pilosula* (Franch.) Nannf. 15g, *Angelica sinensis* (Oliv.) Diels 15g, *Salvia miltiorrhiza* Bunge 15g, *Psoralea corylifolia* L. 12g, *Dioscorea opposita* Thunb. 12g, *Dipsacus asper* Wall. ex Henry 12g, *Atractylodes macrocephala* Koidz. 10g, *Cynomorium songaricum* Rupr. 10g, *Acorus tatarinowii* Schott 10g, *Epimedium brevicornu* Maxim. 10g, *Cuscuta australis* R.Br. 10g, *Ligusticum chuanxiong* Hort. 10g, *Prunus persica* (L.) Batsch 10g, Curcuma phaeocaulis Val. 10g, *Gleditsia sinensis* Lam. 10g, Pinelliae Rhizoma Praeparatum 10g, *Citrus reticulata* Blanco 10g | Decoction |
| Duan, X (2020) | Shiying Yulin Decoction | Fluoritum 30g, *Epimedium brevicornu* Maxim. 30g, *Cuscuta australis* R.Br. 9g, *Dipsacus asper* Wall. ex Henry 15g, *Cinnamomum cassia* Presl 9g, *Lycium barbarum* L. 9g, *Angelica sinensis* (Oliv.) Diels 15g, *Paeonia lactiflora* Pall. 9g, Paeoniae Radix Rubra 9g, *Ligusticum chuanxiong* Hort. 12g, *Cyperus rotundus* L. 9g, *Cyathula officinalis* Kuan 15g, *Eucommia ulmoides* Oliv. 15g, *Glycyrrhiza uralensis* Fisch. 9g | Decoction |
| Fu, R (2020) | Jianpi Huatan Decoction | *Atractylodes lancea* (Thunb.) DC. 10g, *Cyperus rotundus* L. 10g, *Citrus reticulata* Blanco 10g, *Pinellia ternata* (Thunb.) Breit. 10g, *Poria cocos* (Schw.) Wolf 10g, *Codonopsis pilosula* (Franch.) Nannf. 15g, *Salvia miltiorrhiza* Bunge 15g, *Angelica sinensis* (Oliv.) Diels 10g, Paeoniae Radix Rubra 15g, *Ligusticum chuanxiong* Hort. 6g, Rehmanniae Radix Praeparata 10g, *Curculigo orchioides* Gaertn. 10g, *Epimedium brevicornu* Maxim. 10g, *Crataegus pinnatifida* Bge. 10g, *Gallus gallus domesticus* Brisson 10g | Granula |
| He, J (2020) | Jianpi Bushen Decoction | *Astragalus membranaceus* (Fisch.) Bge. var. *mongholicus* (Bge.) Hsiao 20g, *Cistanche deserticola* Y.C.Ma 30g, Paeoniae Radix Rubra 20g, *Poria cocos* (Schw.) Wolf 15g, *Angelica sinensis* (Oliv.) Diels 20g, *Cuscuta australis* R.Br. 15g, *Epimedium brevicornu* Maxim. 15g | Decoction? |
| Wang, S (2020) | Bushen Quyu Huatan Decoction | *Epimedium brevicornu* Maxim. 10g, *Cuscuta australis* R.Br. 10g, *Cornus officinalis* Sieb. et Zucc. 10g, *Lycium barbarum* L. 10g, *Dioscorea opposita* Thunb. 10g, Paeoniae Radix Rubra 10g, *Paeonia lactiflora* Pall. 10g, Rehmanniae Radix Praeparata 15g, *Prunus persica* (L.) Batsch 10g, *Carthamus tinctorius* L. 10g, *Angelica sinensis* (Oliv.) Diels 10g, *Poria cocos* (Schw.) Wolf 10g, *Paeonia suffruticosa* Andr. 10g, *Gleditsia sinensis* Lam. 10g, *Atractylodes lancea* (Thunb.) DC. 12g, *Citrus reticulata* Blanco 6g | Decoction |
| Lin, H (2020) | Cangfu Daotan Decoction | *Prunella vulgaris* L. 6g, *Citrus aurantium* L. 6g, *Arisaema erubescens* (Wall.) Schott 6g, Massa Medicata Fermentata 6g, *Angelica sinensis* (Oliv.) Diels 6g, *Pinellia ternata* (Thunb.) Breit. 6g, *Glycyrrhiza uralensis* Fisch. 6g, *Ligusticum chuanxiong* Hort. 6g, *Cyperus rotundus* L. 15g, *Atractylodes lancea* (Thunb.) DC. 15g, *Poria cocos* (Schw.) Wolf 10g, *Citrus reticulata* Blanco 10g, *Prunus persica* (L.) Batsch 10g, *Zingiber officinale* Rosc. 3g | Decoction |
| Hou, X (2020) | Bushen Qutan Decoction | *Epimedium brevicornu* Maxim. 15g, Fluoritum 20g, *Morinda officinalis* How. 15g, Cervi Cornu Praeparatum 12g, *Cuscuta australis* R.Br. 15g, *Atractylodes lancea* (Thunb.) DC. 15g, *Poria cocos* (Schw.) Wolf 15g, *Citrus reticulata* Blanco 12g, Pinelliae Rhizoma Praeparatum cum Alumine 12g, *Gleditsia sinensis* Lam. 12g, *Cyathula officinalis* Kuan 15g, *Angelica sinensis* (Oliv.) Diels 12g, *Cyperus rotundus* L. 15g, *Crataegus pinnatifida* Bge. 20g, *Zanthoxylum bungeanum* Maxim. 3g, *Glycyrrhiza uralensis* Fisch. 6g | Decoction |
| Shi, Q (2020) | Bushen Huatan Decoction | Rehmanniae Radix Praeparata 15g, *Ligusticum chuanxiong* Hort. 15g, *Epimedium brevicornu* Maxim. 15g, *Angelica sinensis* (Oliv.) Diels 15g, *Cyperus rotundus* L. 10g, *Poria cocos* (Schw.) Wolf 10g, *Cuscuta australis* R.Br. 10g, *Pinellia ternata* (Thunb.) Breit. 8g, *Lycium barbarum* L. 8g, *Atractylodes macrocephala* Koidz. 8g, *Salvia miltiorrhiza* Bunge 6g, *Dioscorea opposita* Thunb. 6g | Decoction |
| Li, Y (2020) | Self-formulated Prescription | Rehmanniae Radix Praeparata 20g, *Codonopsis pilosula* (Franch.) Nannf. 20g, *Lycium barbarum* L. 15g, *Epimedium brevicornu* Maxim. 15g, *Cistanche deserticola* Y.C.Ma 15g, *Angelica sinensis* (Oliv.) Diels 15g, *Atractylodes macrocephala* Koidz. 15g, *Poria cocos* (Schw.) Wolf 15g, *Sinapis alba* L. 10g, *Citrus reticulata* Blanco 10g, Cervi Cornu Praeparatum 10g, Citri Reticulatae Pericarpium viride 5g, *Glycyrrhiza uralensis* Fisch. 6g | Decoction |
| Deng, X (2019) | Danggui Dihuang Decoction combined with Taoren Siwu Decoction | *Angelica sinensis* (Oliv.) Diels 15g, Rehmanniae Radix Praeparata 15g, *Cornus officinalis* Sieb. et Zucc. 10g, *Dioscorea opposita* Thunb. 15g, *Eucommia ulmoides* Oliv. 15g, *Achyranthes bidentata* Bl. 10g, *Cuscuta australis* R.Br. 15g, *Prunus persica* (L.) Batsch 15g, *Carthamus tinctorius* L. 5g, *Ligusticum chuanxiong* Hort. 5g, *Paeonia lactiflora* Pall. 10g, *Citrus reticulata* Blanco 5g, Pinelliae Rhizoma Praeparatum cum Zingibere et Alumine 5g, *Glycyrrhiza uralensis* Fisch. 5g | Decoction |
| Li, H (2019a) | Yishen Huatan Decoction | *Cuscuta australis* R.Br. 20g, *Epimedium brevicornu* Maxim. 15g, *Astragalus membranaceus* (Fisch.) Bge. var. *mongholicus* (Bge.) Hsiao 15g, *Rehmannia glutinosa* Libosch. 12g, Rehmanniae Radix Praeparata 12g, *Angelica sinensis* (Oliv.) Diels 12g, *Atractylodes lancea* (Thunb.) DC. 12g, *Atractylodes macrocephala* Koidz. 12g, *Citrus reticulata* Blanco 12g, *Acorus tatarinowii* Schott 12g, *Pinellia ternata* (Thunb.) Breit. 12g | Decoction |
| Chen, Y (2019b) | Self-formulated Prescription | Fluoritum 30g, *Epimedium brevicornu* Maxim. 20g, Cervi Cornu Praeparatum 20g, Rehmanniae Radix Praeparata 12g, Pinelliae Rhizoma Praeparatum cum Alumine 9g, Citri Exocarpium Rubrum 12g, *Gleditsia sinensis* Lam. 10g, *Atractylodes macrocephala* Koidz. 20g, *Poria cocos* (Schw.) Wolf 20g, *Angelica sinensis* (Oliv.) Diels 12g, *Ligusticum chuanxiong* Hort. 15g, Paeoniae Radix Rubra 20g, *Cyperus rotundus* L. 12g, *Crataegus pinnatifida* Bge. 10g, *Zanthoxylum bungeanum* Maxim. 1.5g | Decoction |
| Yang, Y (2019) | Xiaozhi Decoction | *Epimedium brevicornu* Maxim. 15g, Pinelliae Rhizoma Praeparatum cum Zingibere et Alumine 9g, *Bupleurum chinense* DC. 9g, *Scutellaria baicalensis* Georgi 9g, *Paeonia lactiflora* Pall. 9g, *Rheum palmatum* L. 6g, *Citrus aurantium* L. 15g, *Ligusticum chuanxiong* Hort. 9g, *Sargassum fusiforme* (Harv.) Setch. et Gardner 15g, *Cassia obtusifolia* L. 15g, *Taxillus chinensis* (DC.) Danser 15g, *Gynostemma pentaphyllum* (Thunb.) Makino 15g | Decoction |
| Di, X (2019) | Cangfu Daotan Decoction | *Atractylodes lancea* (Thunb.) DC. 12g, *Cyperus rotundus* L. 10g, *Citrus reticulata* Blanco 12g, *Pinellia ternata* (Thunb.) Breit. 12g, Arisaema cum Bile 6g, *Poria cocos* (Schw.) Wolf 15g, *Citrus aurantium* L. 10g, Massa Medicata Fermentata 10g, *Zingiber officinale* Rosc. 6g, *Glycyrrhiza uralensis* Fisch. 6g | Decoction |
| Huang, C (2019) | Modified Cangfu Daotan Decoction | *Atractylodes lancea* (Thunb.) DC. 10g, Arisaema cum Bile 5g, Pinelliae Rhizoma Praeparatum 10g, *Citrus aurantium* L. 6g, *Cyperus rotundus* L. 10g, *Citrus reticulata* Blanco 10g, *Poria cocos* (Schw.) Wolf 15g, *Glycyrrhiza uralensis* Fisch. 10g, *Zingiber officinale* Rosc. 6g, *Gleditsia sinensis* Lam. 10g, *Taxillus chinensis* (DC.) Danser 10g, *Fritillaria thunbergii* Miq. 10g, *Cornus officinalis* Sieb. et Zucc. 10g, *Epimedium brevicornu* Maxim. 10g, *Cuscuta australis* R.Br. 10g | Decoction |
| Chen, Y (2019a) | Cangfu Daotan Decoction | *Atractylodes lancea* (Thunb.) DC. 20g, *Cyperus rotundus* L. 20g, *Poria cocos* (Schw.) Wolf 20g, Rehmanniae Radix Praeparata 20g, Cervi Cornu Colla 20g, *Citrus aurantium* L. 10g, *Citrus reticulata* Blanco 10g, *Ligusticum chuanxiong* Hort. 10g, Pinelliae Rhizoma Praeparatum 10g, Arisaema cum Bile 10g, *Glycyrrhiza uralensis* Fisch. 3g | Decoction |
| Zhang, Q (2019) | Modified Cangfu Daotan Decoction | *Atractylodes lancea* (Thunb.) DC. 6g, *Cyperus rotundus* L. 10g, *Citrus reticulata* Blanco 10g, Pinelliae Rhizoma Praeparatum cum Zingibere et Alumine 12g, *Poria cocos* (Schw.) Wolf 12g, *Citrus aurantium* L. 15g, Bambusa tuldoides Munro 15g, *Angelica sinensis* (Oliv.) Diels 10g, *Salvia miltiorrhiza* Bunge 15g, *Cuscuta australis* R.Br. 15g, *Cistanche deserticola* Y.C.Ma 15g, *Epimedium brevicornu* Maxim. 15g, *Polygonum cuspidatum* Siebold et Zucc. 20g, *Coptis chinensis* Franch. 3g | Decoction |
| Bai, R (2018) | Qihuang Zengmin Decoction | *Astragalus membranaceus* (Fisch.) Bge. var. *mongholicus* (Bge.) Hsiao 30g, Rehmanniae Radix Praeparata 30g, *Polygonatum kingianum* Coll.et Hemsl. 30g, *Coptis chinensis* Franch. 6g, *Panax notoginseng* (Burk.) F. H. Chen 12g, *Atractylodes lancea* (Thunb.) DC. 15g, *Gynostemma pentaphyllum* (Thunb.) Makino 15g | Decoction |
| Zhang, H (2018) | Modified Cangfu Daotan Decoction | *Atractylodes lancea* (Thunb.) DC. 10g, *Cyperus rotundus* L. 15g, Arisaema cum Bile 10g, *Acorus tatarinowii* Schott 10g, *Citrus reticulata* Blanco 6g, Pinelliae Rhizoma Praeparatum 10g, *Salvia miltiorrhiza* Bunge 15g, *Cuscuta australis* R.Br. 20g, *Poria cocos* (Schw.) Wolf 15g, Rehmanniae Radix Praeparata 15g, *Taxillus chinensis* (DC.) Danser 15g, *Crataegus pinnatifida* Bge. 15g, *Zingiber officinale* Rosc. 6g, *Glycyrrhiza uralensis* Fisch. 6g | Decoction |
| Yang, Z (2018) | Modified Guizhi Fuling Pill Combined with Danggui Shaoyao Decoction | *Cinnamomum cassia* Presl 30g, *Poria cocos* (Schw.) Wolf 60g, Paeoniae Radix Rubra 15g, *Prunus persica* (L.) Batsch 10g, *Paeonia suffruticosa* Andr. 10g, *Angelica sinensis* (Oliv.) Diels 15g, *Ligusticum chuanxiong* Hort. 10g, *Atractylodes macrocephala* Koidz. 30g, *Alisma orientale* (Sam.) Juzep. 15g | Decoction |
| Xie, P (2018) | Bushen Shugan Huayu Qutan Decoction | *Cistanche deserticola* Y.C.Ma 20g, *Ligustrum lucidum* Ait. 15g, *Cuscuta australis* R.Br. 15g, *Lycium barbarum* L. 10g, Rehmanniae Radix Praeparata 10g, *Bupleurum chinense* DC. 10g, *Paeonia lactiflora* Pall. 10g, *Salvia miltiorrhiza* Bunge 10g, *Ligusticum chuanxiong* Hort. 10g, *Lycopus lucidus* Turcz. var. hirtus Regel 15g, *Poria cocos* (Schw.) Wolf 15g, *Pinellia ternata* (Thunb.) Breit. 15g, *Acorus tatarinowii* Schott 10g, *Crataegus pinnatifida* Bge. 15g | Decoction |
| Liu, Y (2018) | Self-formulated Prescription | *Astragalus membranaceus* (Fisch.) Bge. var. *mongholicus* (Bge.) Hsiao 20g, Paeoniae Radix Rubra 20g, *Angelica sinensis* (Oliv.) Diels 20g, *Poria cocos* (Schw.) Wolf 15g, *Cuscuta australis* R.Br. 15g, *Epimedium brevicornu* Maxim. 15g, *Cistanche deserticola* Y.C.Ma 30g | Decoction |
| Xu, J (2017) | Tanzhixiao Granula | *Atractylodes lancea* (Thunb.) DC., *Poria cocos* (Schw.) Wolf, *Alisma orientale* (Sam.) Juzep., *Cuscuta australis* R.Br., *Epimedium brevicornu* Maxim. | Decoction |
| Fu, Y (2017) | Modified Cangfu Daotan Decoction | *Atractylodes lancea* (Thunb.) DC. 10g, *Cyperus rotundus* L. 10g, Pinelliae Rhizoma Praeparatum 10g, *Citrus reticulata* Blanco 6g, *Acorus tatarinowii* Schott 10g, *Poria cocos* (Schw.) Wolf 30g, *Astragalus membranaceus* (Fisch.) Bge. var. *mongholicus* (Bge.) Hsiao 30g, *Gleditsia sinensis* Lam. 10g, *Epimedium brevicornu* Maxim. 15g, *Angelica sinensis* (Oliv.) Diels 10g, *Salvia miltiorrhiza* Bunge 15g | Decoction |
| Zhou, D (2017) | Heqi Powder | *Taxillus chinensis* (DC.) Danser, *Astragalus membranaceus* (Fisch.) Bge. var. *mongholicus* (Bge.) Hsiao, *Dioscorea opposita* Thunb., *Prunus persica* (L.) Batsch, *Cassia obtusifolia* L., *Acorus tatarinowii* Schott, *Polygonum multiflorum* Thunb., *Pinellia ternata* (Thunb.) Breit., *Carthamus tinctorius* L., *Angelica sinensis* (Oliv.) Diels, *Benincasa hispida* (Thunb.) Cogn., *Curculigo orchioides* Gaertn., *Epimedium brevicornu* Maxim., *Cuscuta australis* R.Br. | Decoction |
| Liu, M (2017) | Bushen Huatan Decoction | Fluoritum 15g, *Epimedium brevicornu* Maxim. 10g, *Citrus reticulata* Blanco 10g, Pinelliae Rhizoma Praeparatum 10g, *Poria cocos* (Schw.) Wolf 10g, *Atractylodes lancea* (Thunb.) DC. 10g, *Cyathula officinalis* Kuan 10g, Paeoniae Radix Rubra 10g, *Angelica sinensis* (Oliv.) Diels 10g, *Salvia miltiorrhiza* Bunge 10g, *Ligusticum chuanxiong* Hort. 10g, *Cyperus rotundus* L. 10g, *Crataegus pinnatifida* Bge. 10g | Decoction |
| Ye, L (2017) | Sanhuang Decoction | *Coptis chinensis* Franch. 3g, *Scutellaria baicalensis* Georgi 10g, *Rheum palmatum* L. 6g | Decoction |
| Guo, R (2017) | Cangfu Daotan Decoction | *Atractylodes lancea* (Thunb.) DC. 12g, *Pinellia ternata* (Thunb.) Breit. 10g, *Citrus reticulata* Blanco 9g, *Cyperus rotundus* L. 12g, *Astragalus membranaceus* (Fisch.) Bge. var. *mongholicus* (Bge.) Hsiao 30g, *Poria cocos* (Schw.) Wolf 30g, *Epimedium brevicornu* Maxim. 15g, *Acorus tatarinowii* Schott 12g, *Angelica sinensis* (Oliv.) Diels 15g, *Gleditsia sinensis* Lam. 12g, *Salvia miltiorrhiza* Bunge 12g | Decoction |
| Lin, H (2017) | Huatan Tongmai Decoction | Pinelliae Rhizoma Praeparatum 15g、*Atractylodes lancea* (Thunb.) DC. 15g、*Atractylodes macrocephala* Koidz. 15g、*Poria cocos* (Schw.) Wolf 20g、*Spatholobus suberectus* Dunn 20g、*Angelica sinensis* (Oliv.) Diels 10g、*Salvia miltiorrhiza* Bunge 15g、*Prunus persica* (L.) Batsch 10g、*Lycopus lucidus* Turcz. var. hirtus Regel 15g、*Arca subcrenata* Lischke 10g、*Citrus reticulata* Blanco 6g、*Ligusticum chuanxiong* Hort. 10g、*Cyperus rotundus* L. 15g | Granula |
| Song, C (2016) | Jianpi Lishi Yiqi Yangyin Decoction | *Atractylodes macrocephala* Koidz., *Pinellia ternata* (Thunb.) Breit., *Citrus reticulata* Blanco, *Poria cocos* (Schw.) Wolf, *Dioscorea opposita* Thunb., *Cornus officinalis* Sieb. et Zucc., *Coix lacryma-jobi* L. var. ma-yuen (Roman.) Stapf, *Cassia obtusifolia* L., Rehmanniae Radix Praeparata, *Angelica sinensis* (Oliv.) Diels, *Paeonia lactiflora* Pall., *Astragalus membranaceus* (Fisch.) Bge. var. *mongholicus* (Bge.) Hsiao, *Leonurus japonicus* Houtt., *Dendrobium nobile* Lindl., *Crataegus pinnatifida* Bge., *Trionyx sinensis* Wiegmann, *Cuscuta australis* R.Br., *Glycyrrhiza uralensis* Fisch. | Granula |
| Huang, C (2016) | Buqi Huatan Xingqi Decoction | Rehmanniae Radix Praeparata 15g, *Cornus officinalis* Sieb. et Zucc. 15g, *Dioscorea opposita* Thunb. 15g, *Cuscuta australis* R.Br., *Lycium barbarum* L. 15g, *Eucommia ulmoides* Oliv. 15g, *Atractylodes lancea* (Thunb.) DC. 10g, Pinelliae Rhizoma Praeparatum 10g, *Acorus tatarinowii* Schott 10g, *Poria cocos* (Schw.) Wolf 15g, *Citrus reticulata* Blanco 10g, *Curcuma wenyujin* Y. H. Chen et C. Ling 15g, *Cyperus rotundus* L. 10g, *Citrus aurantium* L. 15g, *Salvia miltiorrhiza* Bunge 10g, *Spatholobus suberectus* Dunn 30g, *Glycyrrhiza uralensis* Fisch. 5g | Granula |
| Huang, J (2016) | Self-formulated Prescription | *Taxillus chinensis* (DC.) Danser 10g, *Equus asinus* L. 6g, *Cuscuta australis* R.Br. 10g, *Dipsacus asper* Wall. ex Henry 10g, *Eucommia ulmoides* Oliv. 10g, *Codonopsis pilosula* (Franch.) Nannf. 10g, *Poria cocos* (Schw.) Wolf 10g, *Dioscorea opposita* Thunb. 10g, *Equus asinus* L. 10g, *Atractylodes macrocephala* Koidz. 10g, *Glycyrrhiza uralensis* Fisch. 6g | Decoction |
| Song, Y (2015) | Yishen Huatan Decoction | *Astragalus membranaceus* (Fisch.) Bge. var. *mongholicus* (Bge.) Hsiao 15g、*Rehmannia glutinosa* Libosch. 12g、Rehmanniae Radix Praeparata 12g、*Angelica sinensis* (Oliv.) Diels 12g、*Atractylodes lancea* (Thunb.) DC. 12g、*Atractylodes macrocephala* Koidz. 20g、*Citrus reticulata* Blanco 12g、*Pinellia ternata* (Thunb.) Breit. 12g、*Acorus tatarinowii* Schott 12g、*Cuscuta australis* R.Br. 20g、*Epimedium brevicornu* Maxim. 15g | Decoction |
| Wang, Q (2015) | Self-formulated Prescription | *Astragalus membranaceus* (Fisch.) Bge. var. *mongholicus* (Bge.) Hsiao 30g, *Atractylodes macrocephala* Koidz. 20g, *Atractylodes lancea* (Thunb.) DC. 15g, *Taxillus chinensis* (DC.) Danser 20g, *Benincasa hispida* (Thunb.) Cogn. 15g, *Crataegus pinnatifida* Bge. 30g, *Lycium barbarum* L. 15g, *Dioscorea opposita* Thunb. 15g, *Coix lacryma-jobi* L. var. ma-yuen (Roman.) Stapf 15g, *Eupolyphaga sinensis* Walker 12g, Pinelliae Rhizoma Praeparatum 12g, *Acorus tatarinowii* Schott 12g | Decoction |
| Yin, Q (2015) | Bushen Huatan Decoction | *Salvia miltiorrhiza* Bunge 30g、*Epimedium brevicornu* Maxim. 30g、*Astragalus membranaceus* (Fisch.) Bge. var. *mongholicus* (Bge.) Hsiao 50g、*Poria cocos* (Schw.) Wolf 30g、*Atractylodes lancea* (Thunb.) DC. 30g | Granula |
| Lu, L (2013) | Chushi Huatan Decoction | *Poria cocos* (Schw.) Wolf, *Pinellia ternata* (Thunb.) Breit., *Citrus reticulata* Blanco, *Atractylodes lancea* (Thunb.) DC., *Cyperus rotundus* L., *Arisaema erubescens* (Wall.) Schott, *Citrus aurantium* L., *Zingiber officinale* Rosc., Massa Medicata Fermentata, *Prunus persica* (L.) Batsch, *Angelica sinensis* (Oliv.) Diels, *Ligusticum chuanxiong* Hort., *Carthamus tinctorius* L., *Prunella vulgaris* L., *Glycyrrhiza uralensis* Fisch. | Decoction |
| Jiao, N (2013) | Zaoshi Huatan Bushen Decoction | *Atractylodes lancea* (Thunb.) DC. 12g, *Atractylodes macrocephala* Koidz. 20g, *Taxillus chinensis* (DC.) Danser 20g, *Crataegus pinnatifida* Bge. 30g, *Cassia obtusifolia* L. 12g, *Acorus tatarinowii* Schott 12g, Arisaema cum Bile 12g, *Sinapis alba* L. 12g, Fluoritum 30g, Human Placenta 12g, Pinelliae Rhizoma Praeparatum 12g, *Alisma orientale* (Sam.) Juzep. 12g | Decoction |
| Feng, C (2009) | Modified Erchen Decoction | *Citrus reticulata* Blanco 10g、*Pinellia ternata* (Thunb.) Breit. 10g、*Poria cocos* (Schw.) Wolf 15g、*Glycyrrhiza uralensis* Fisch. 5g、*Alisma orientale* (Sam.) Juzep. 12g、Arisaema cum Bile 6g、*Citrus aurantium* L. 12g、*Rheum palmatum* L. 10g、*Fritillaria cirrhosa* D.Don 10g、*Epimedium brevicornu* Maxim. 15g | Decoction |
